# Supplementary figures and images for: A causal relationship between sarcopenia and cognitive impairment: A Mendelian randomization study
Source: PLoS One. 2024 Sep 6;19(9):e0309124. doi: 10.1371/journal.pone.0309124 (PMC11379137; doi:10.1371/journal.pone.0309124)

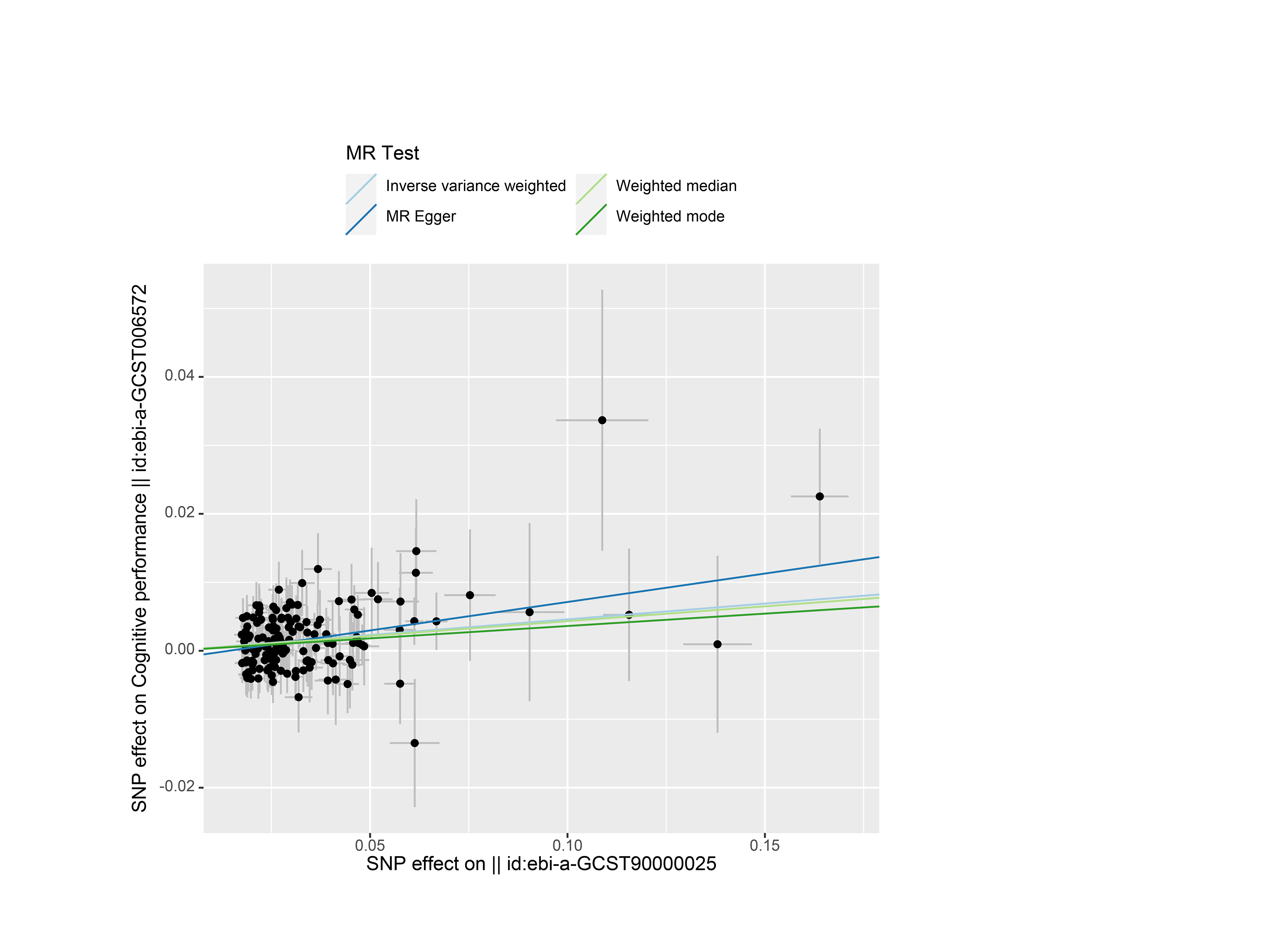

Supplement: S1 Data — (ZIP) [file pone.0309124.s002.zip › Data Sheet/Additional file 1 Scatter plot figure/R1 ALM on cognitive performance.tif]

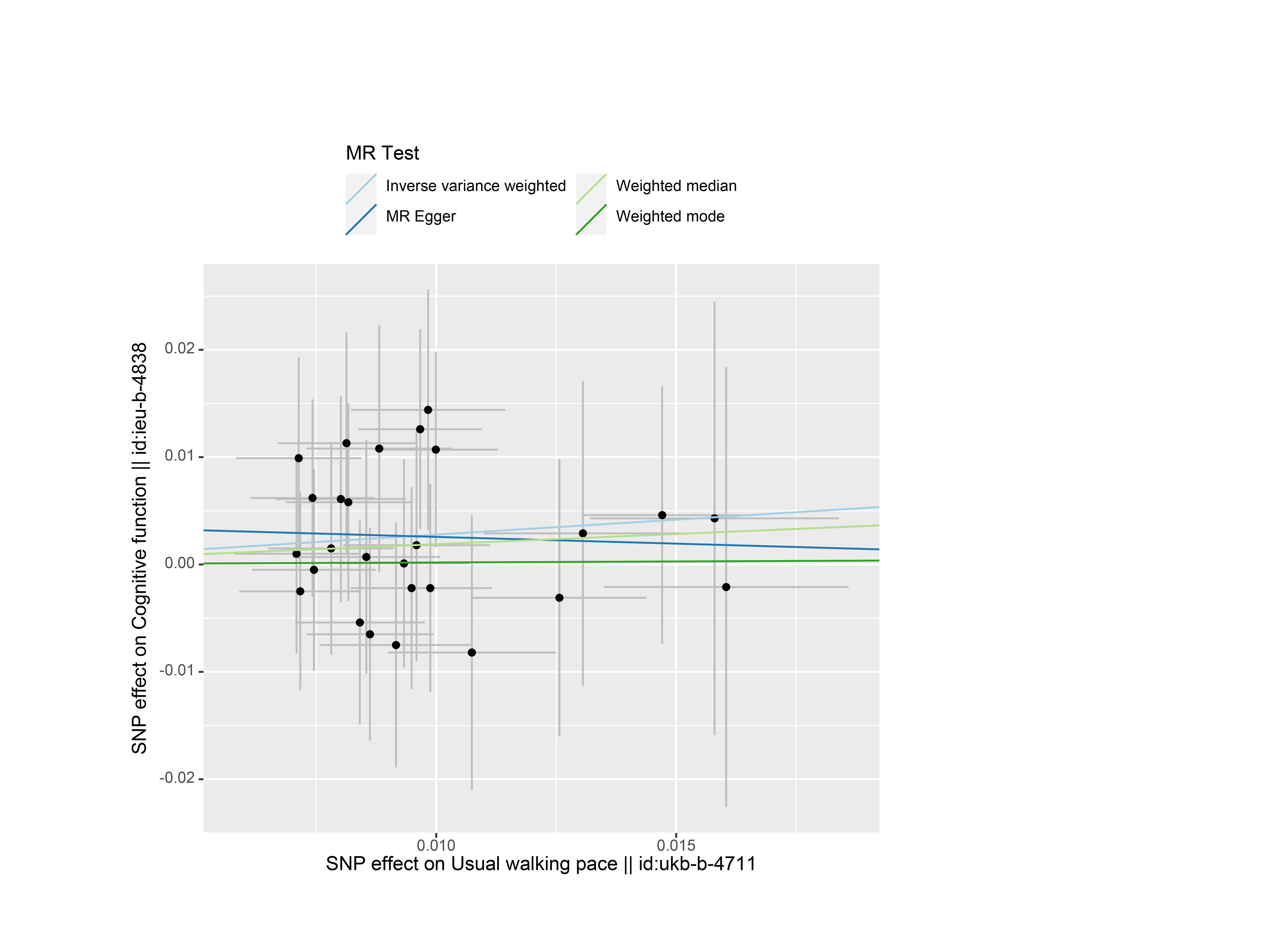

Supplement: S1 Data — (ZIP) [file pone.0309124.s002.zip › Data Sheet/Additional file 1 Scatter plot figure/R10 Walking pace on cognitive function.tif]

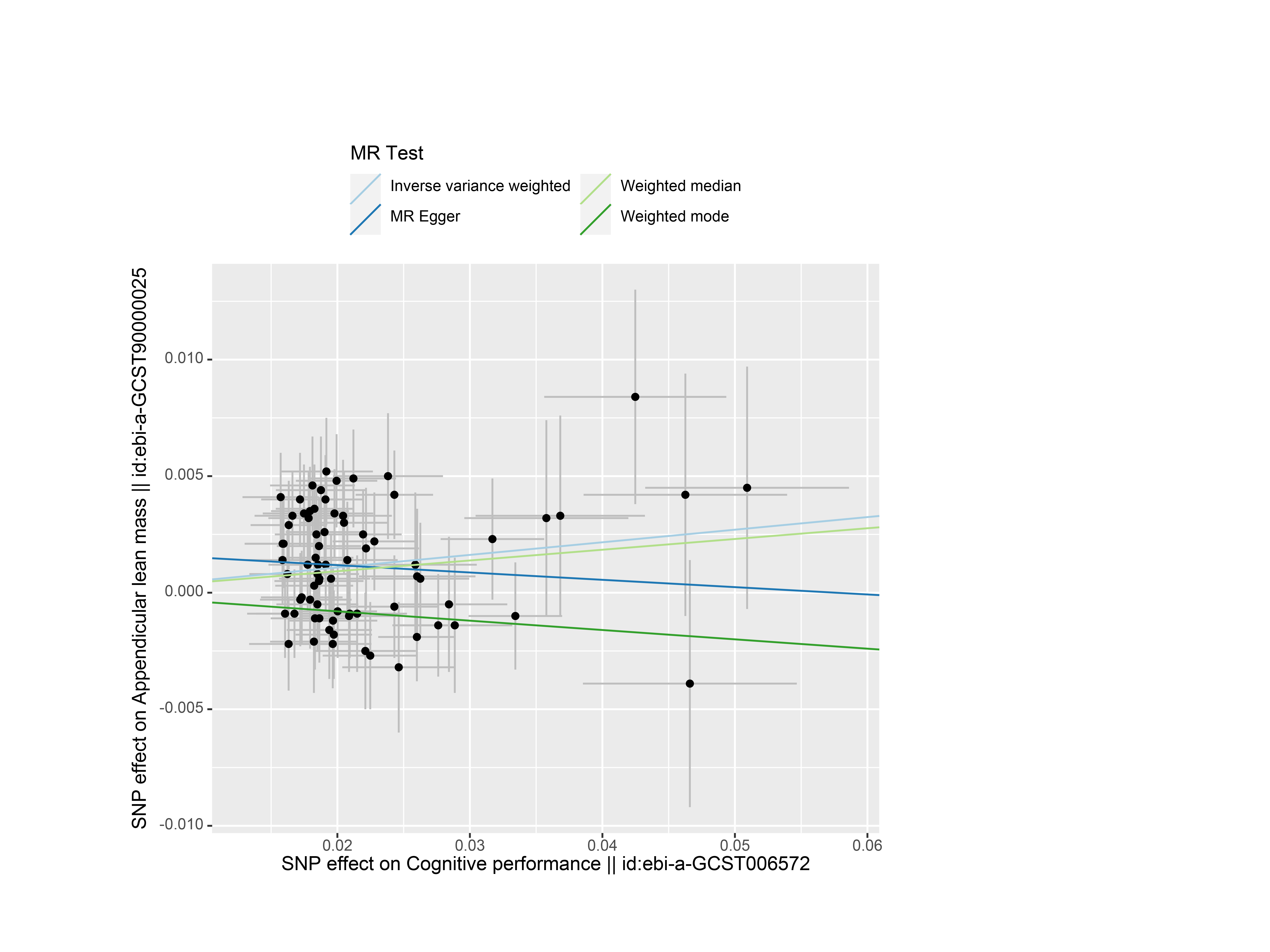

Supplement: S1 Data — (ZIP) [file pone.0309124.s002.zip › Data Sheet/Additional file 1 Scatter plot figure/R11 Cognitive performance on ALM.tif]

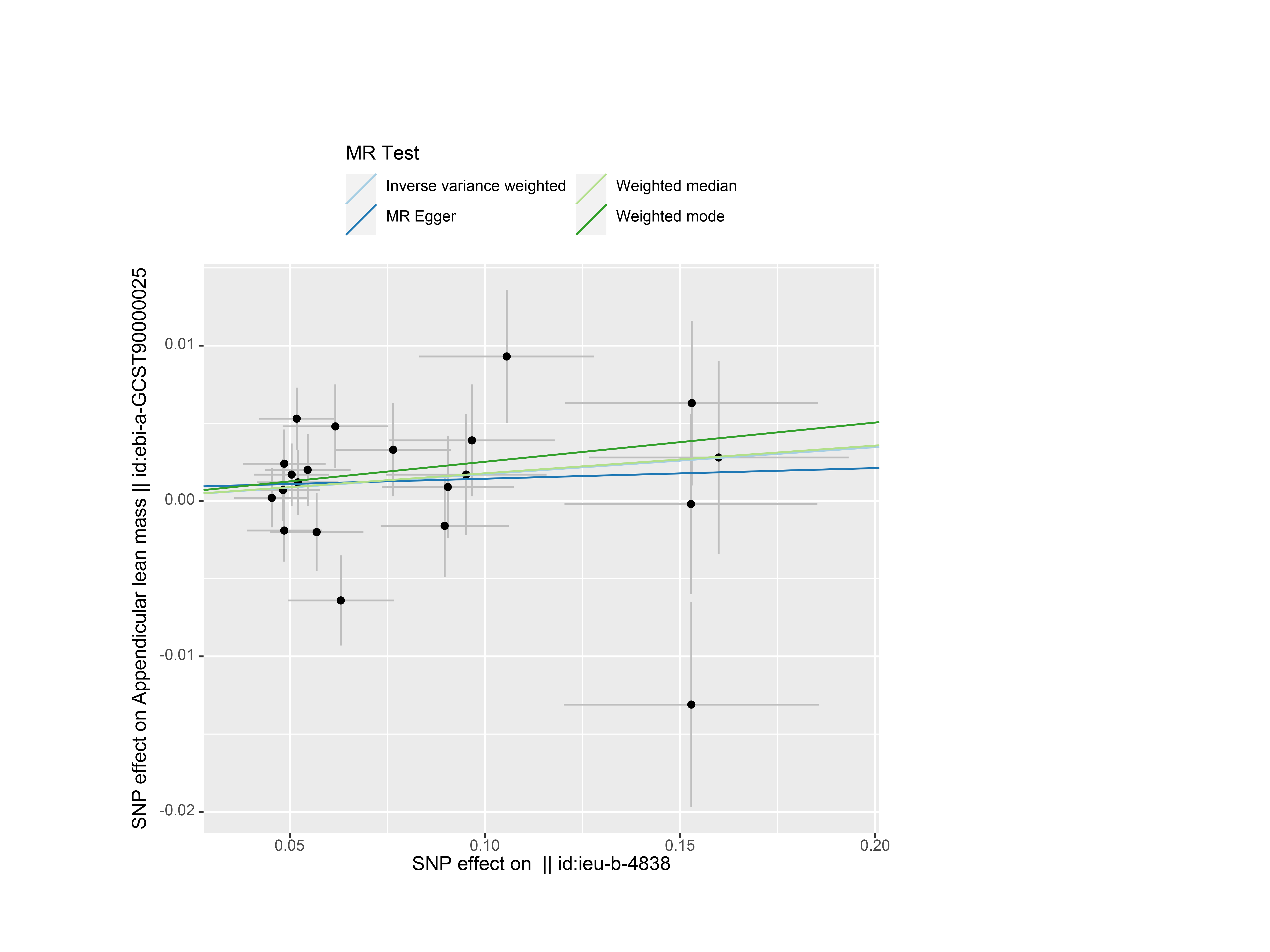

Supplement: S1 Data — (ZIP) [file pone.0309124.s002.zip › Data Sheet/Additional file 1 Scatter plot figure/R12 Cognitive function on ALM.tif]

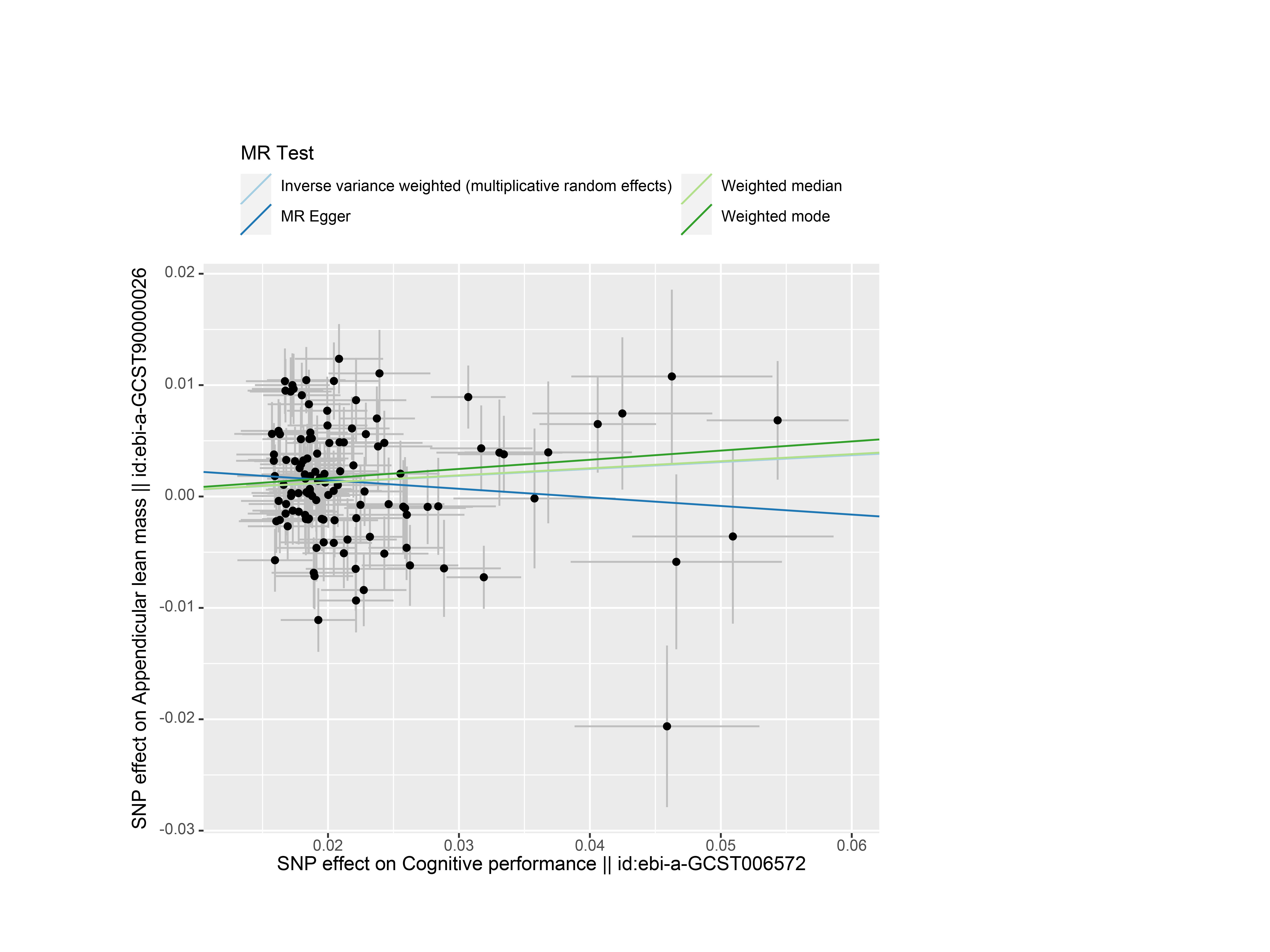

Supplement: S1 Data — (ZIP) [file pone.0309124.s002.zip › Data Sheet/Additional file 1 Scatter plot figure/R13 Cognitive performance on ALM-M.tif]

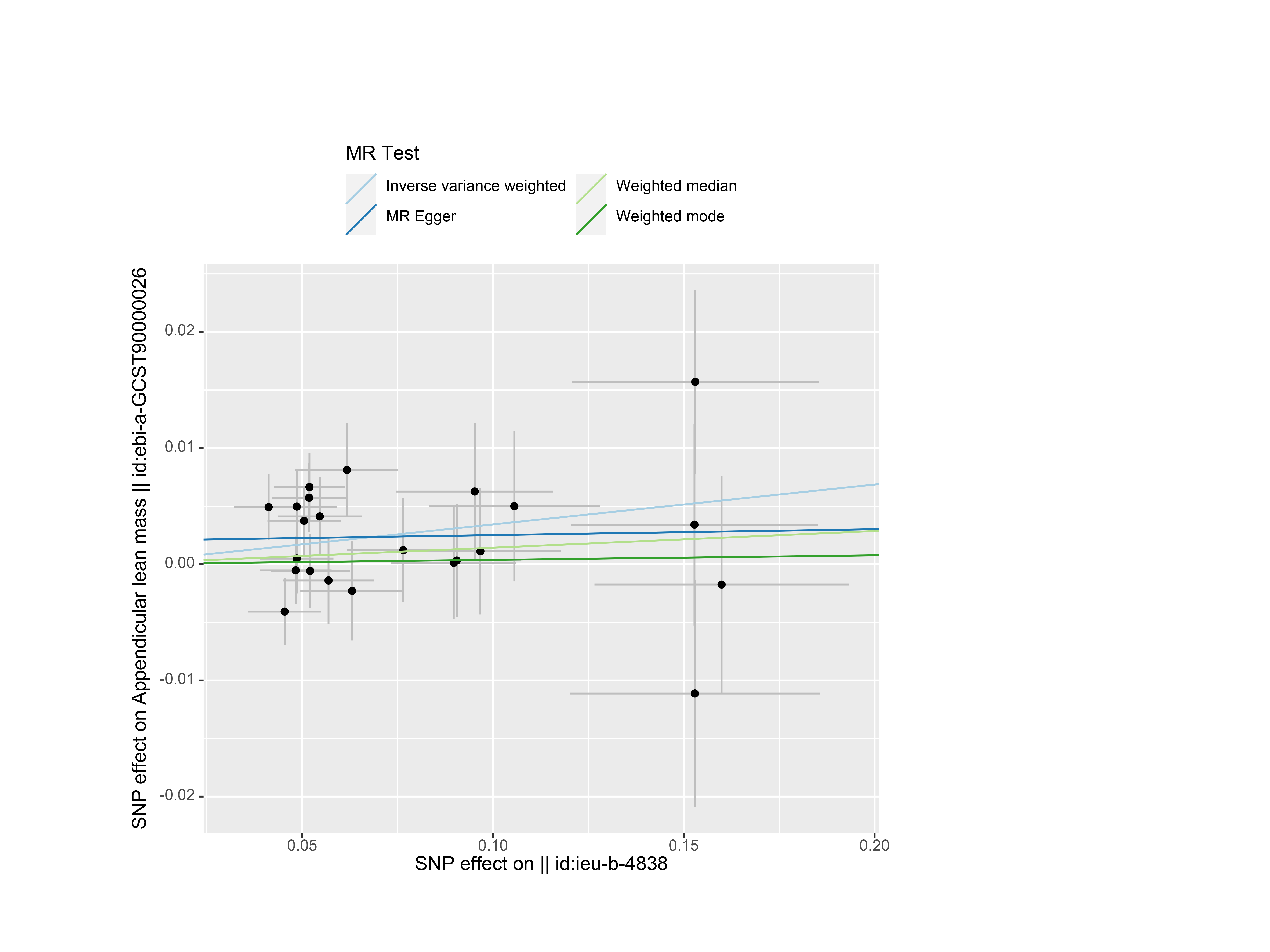

Supplement: S1 Data — (ZIP) [file pone.0309124.s002.zip › Data Sheet/Additional file 1 Scatter plot figure/R14 Cognitive function on ALM-M.tif]

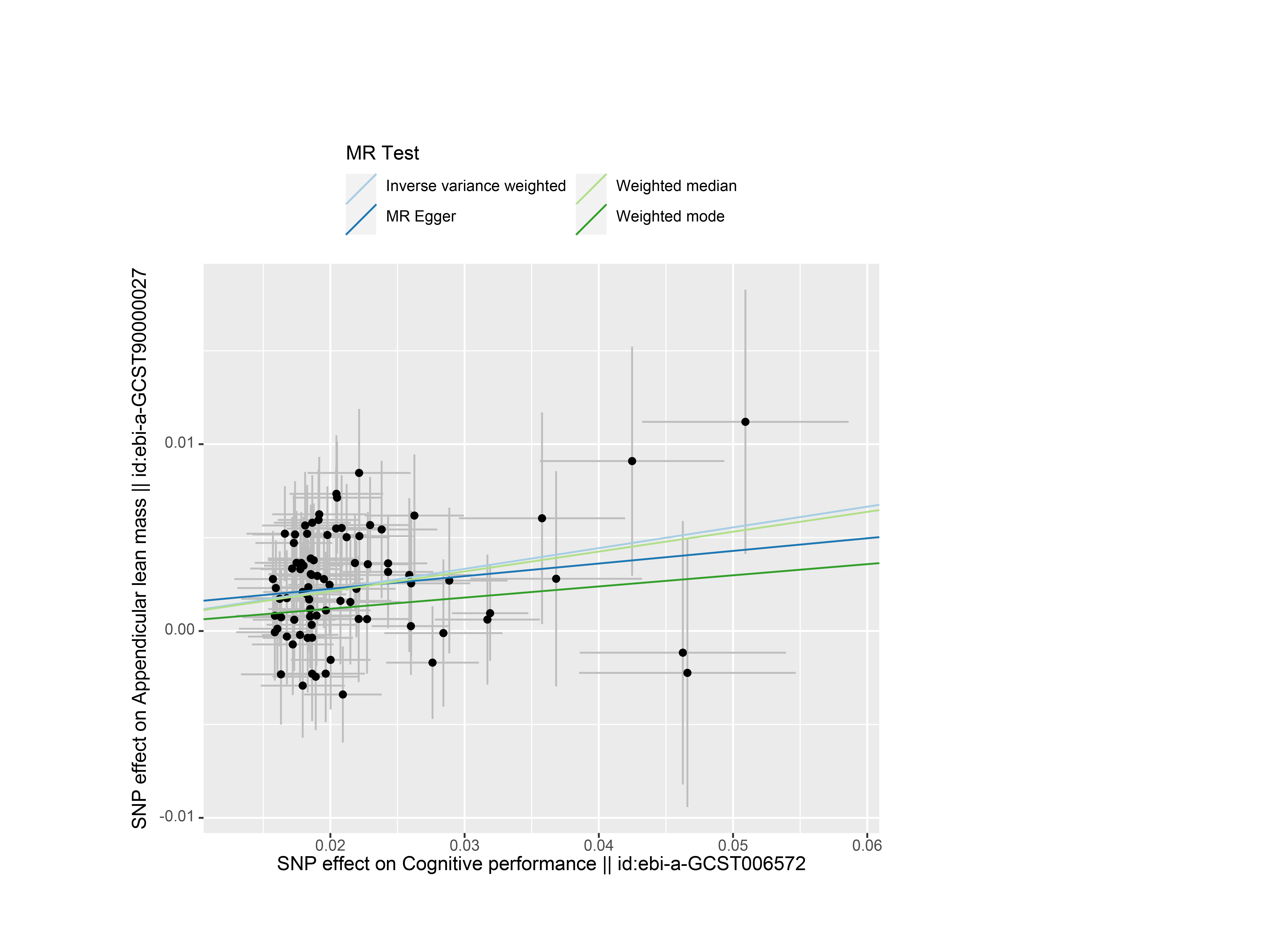

Supplement: S1 Data — (ZIP) [file pone.0309124.s002.zip › Data Sheet/Additional file 1 Scatter plot figure/R15 Cognitive performance on ALM-F.tif]

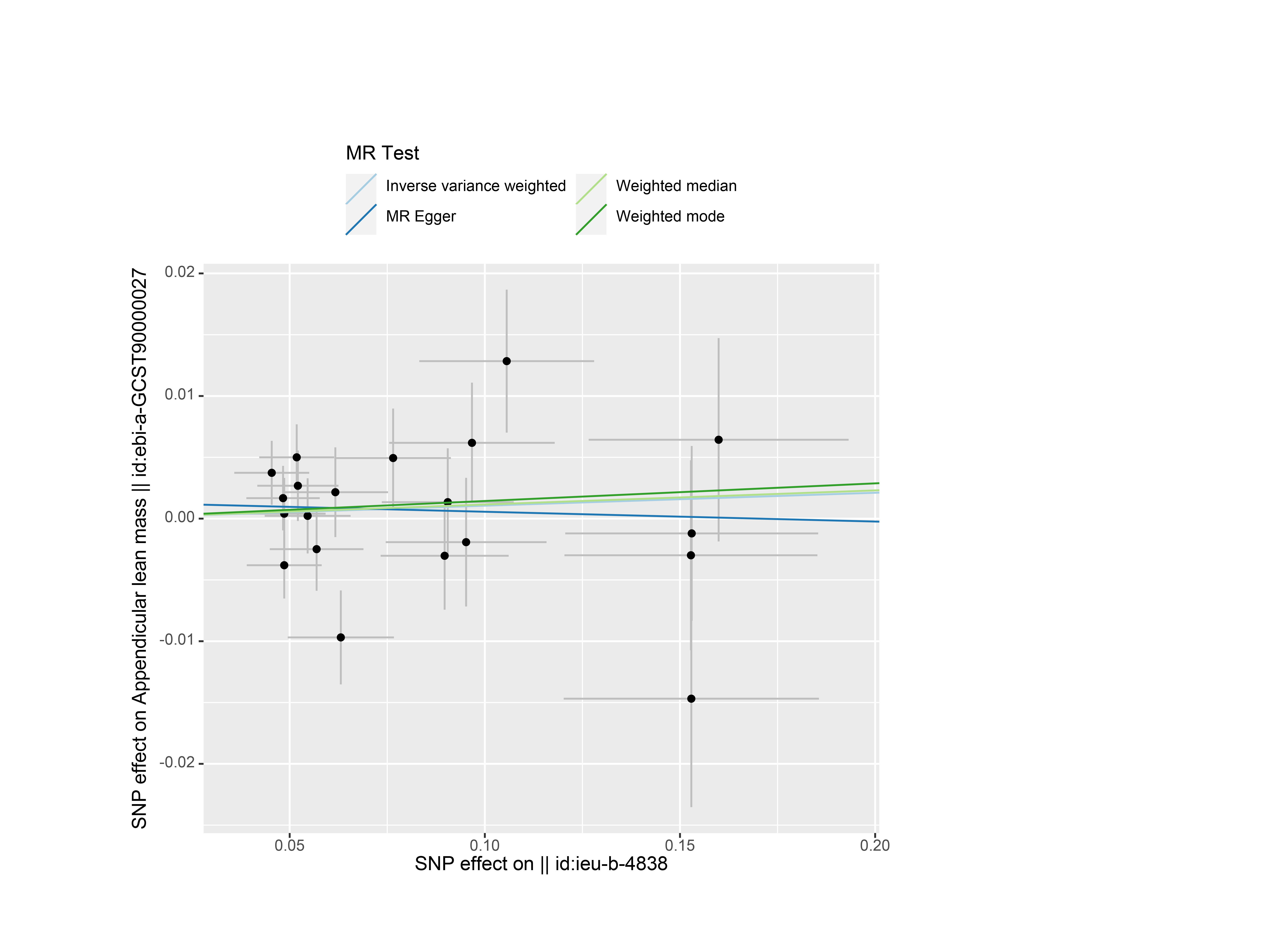

Supplement: S1 Data — (ZIP) [file pone.0309124.s002.zip › Data Sheet/Additional file 1 Scatter plot figure/R16 Cognitive function on ALM-F.tif]

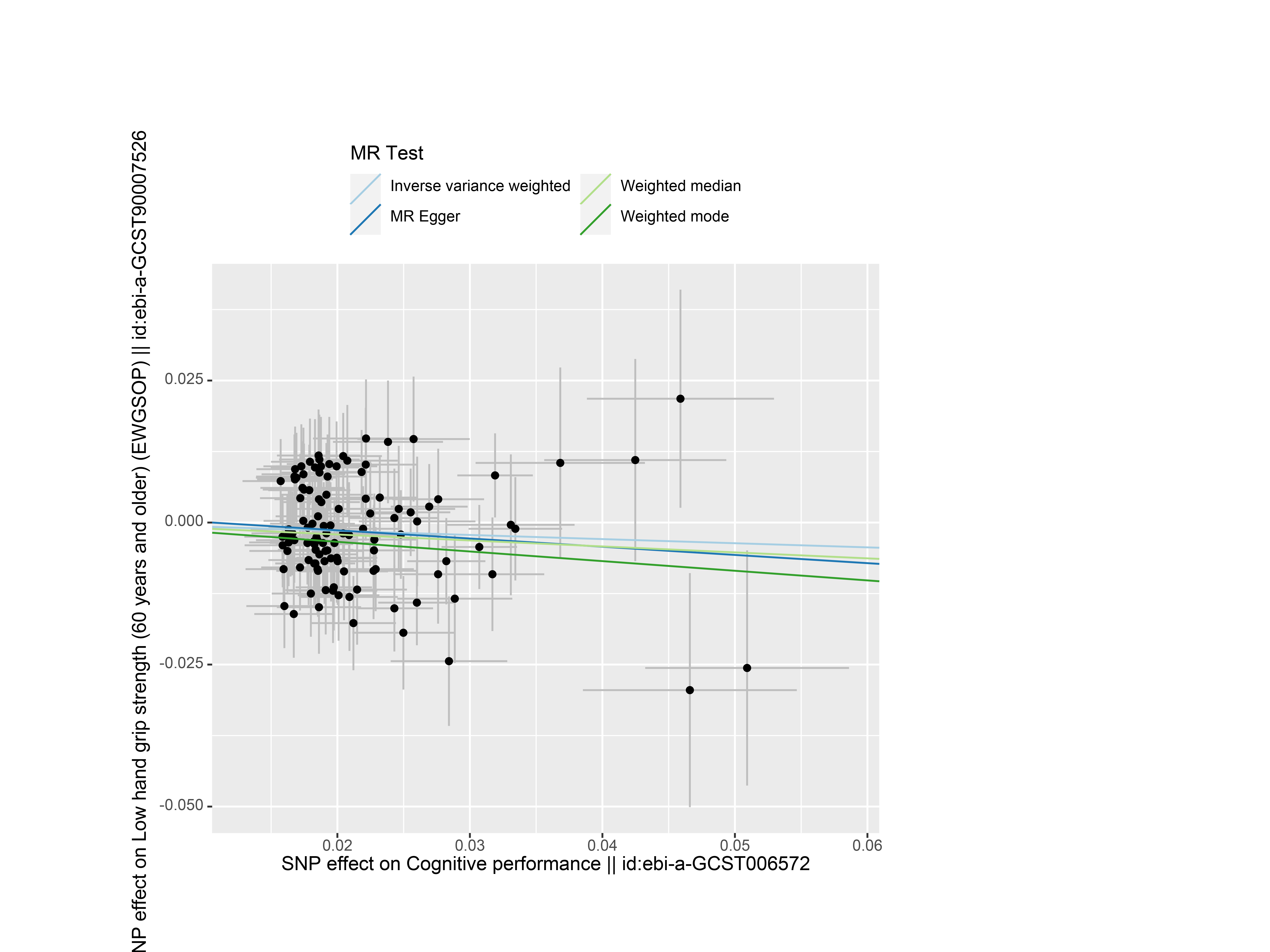

Supplement: S1 Data — (ZIP) [file pone.0309124.s002.zip › Data Sheet/Additional file 1 Scatter plot figure/R17 Cognitive performance on low hand grip strength.tif]

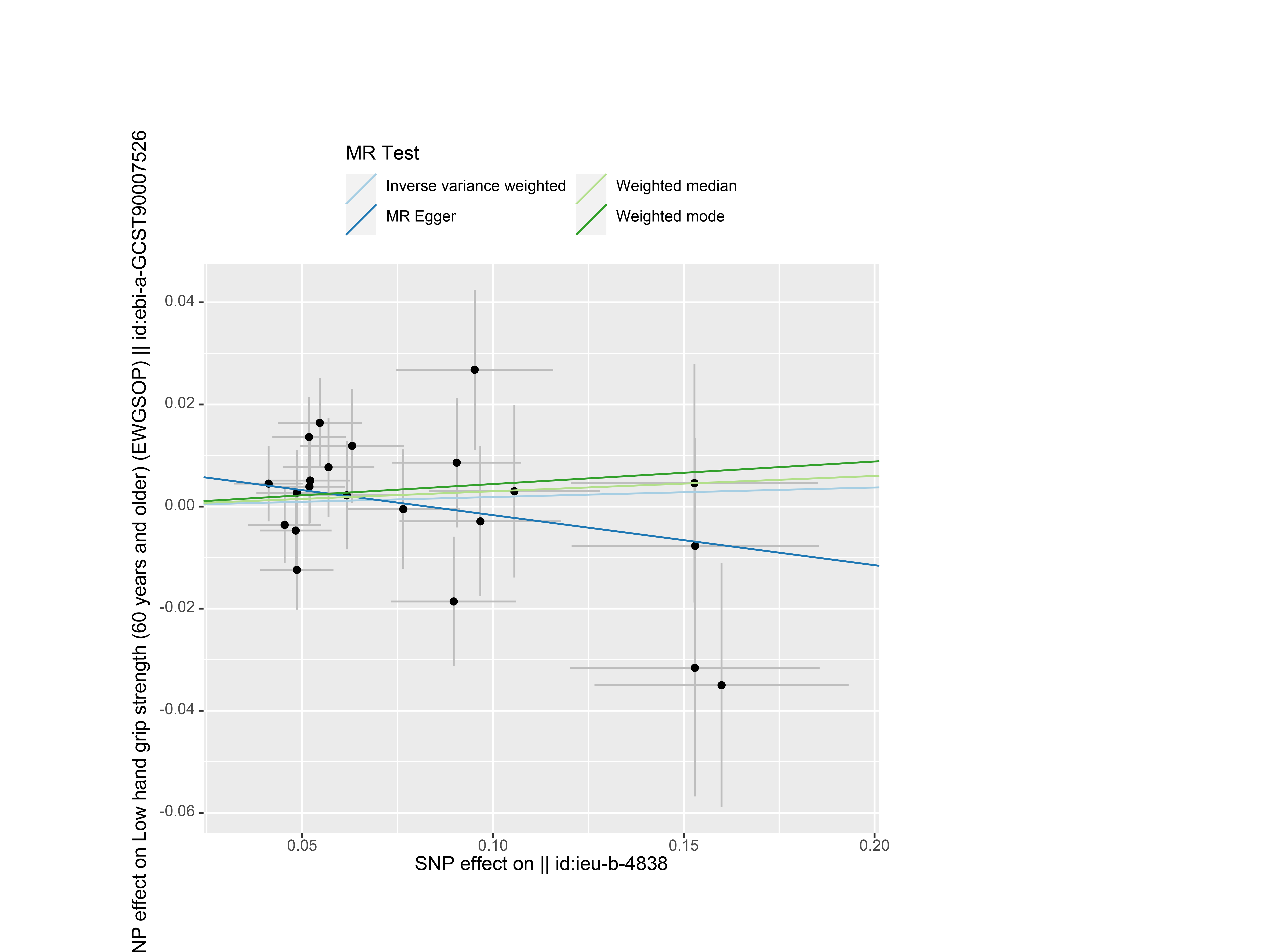

Supplement: S1 Data — (ZIP) [file pone.0309124.s002.zip › Data Sheet/Additional file 1 Scatter plot figure/R18 Cognitive function on low hand grip strength.tif]

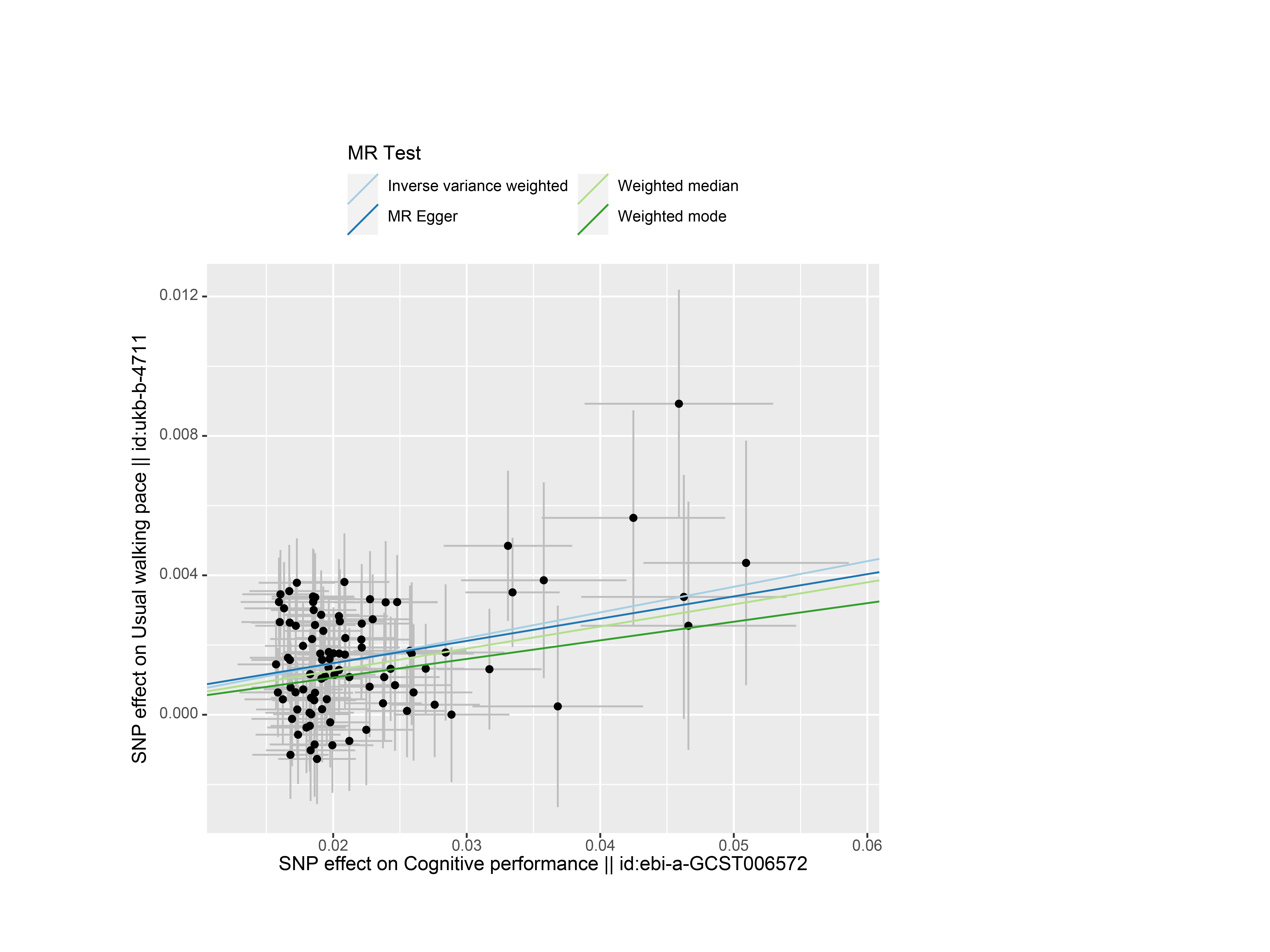

Supplement: S1 Data — (ZIP) [file pone.0309124.s002.zip › Data Sheet/Additional file 1 Scatter plot figure/R19 Cognitive performance on walking pace.tif]

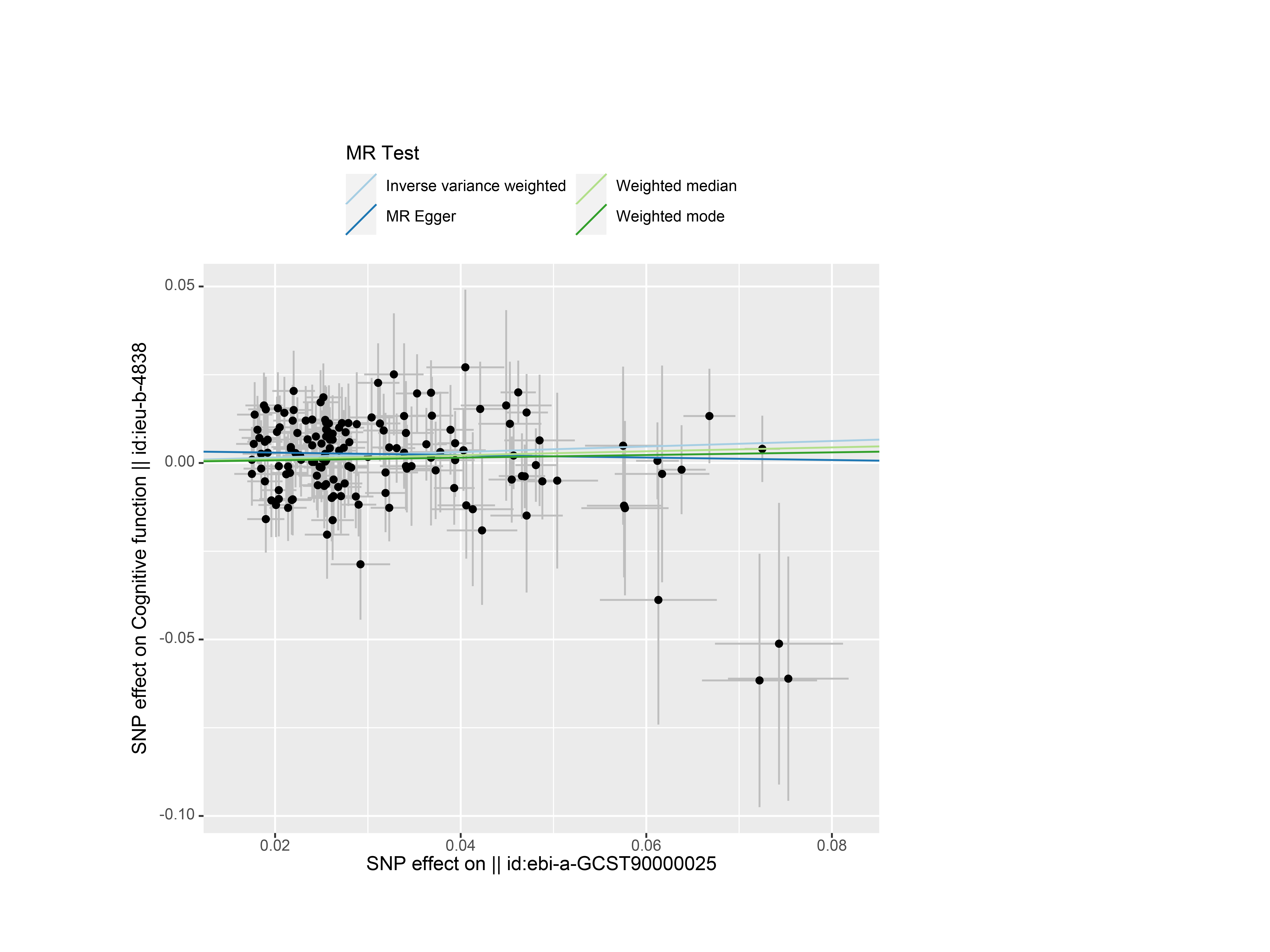

Supplement: S1 Data — (ZIP) [file pone.0309124.s002.zip › Data Sheet/Additional file 1 Scatter plot figure/R2 ALM on cognitive function.tif]

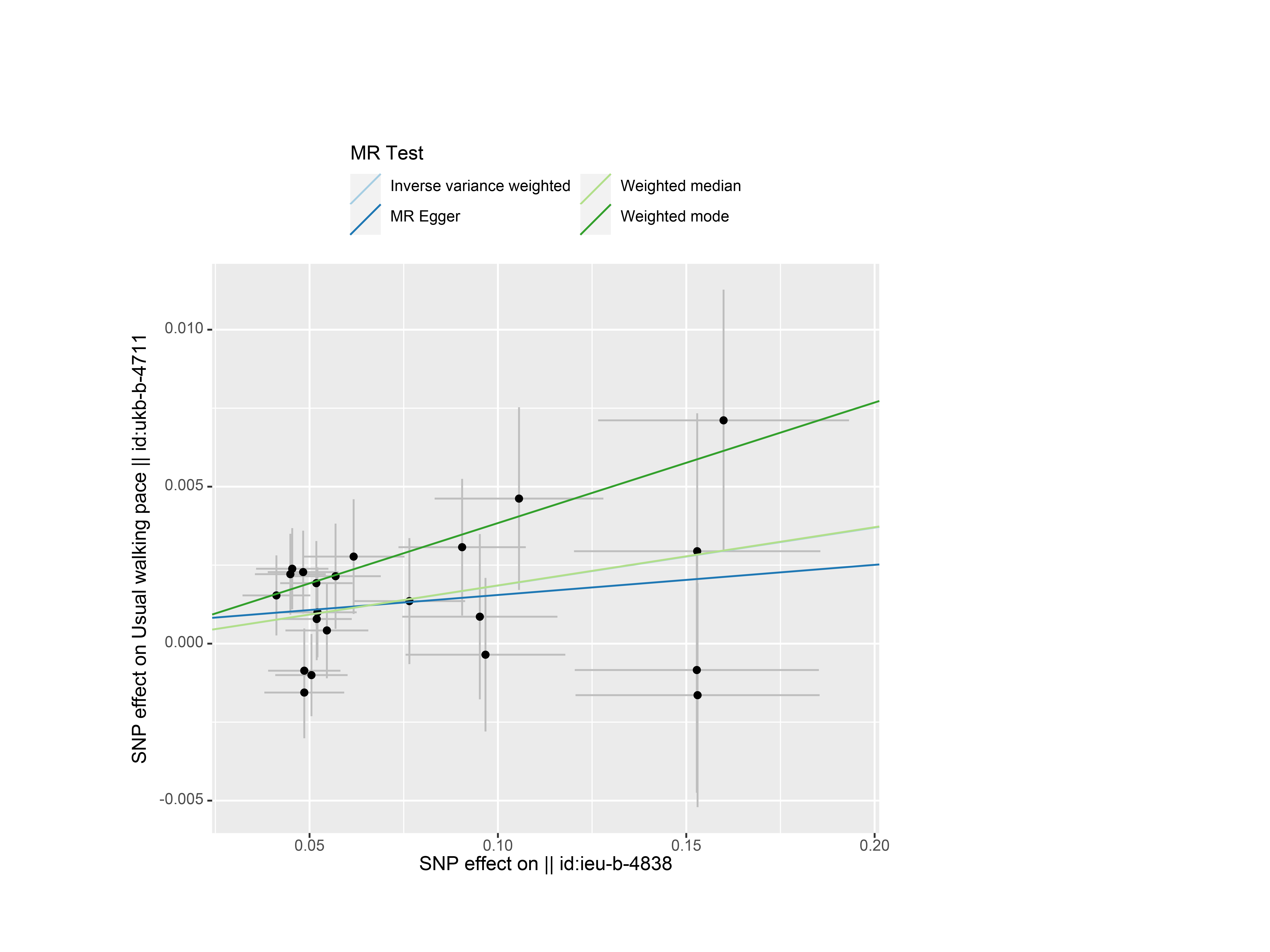

Supplement: S1 Data — (ZIP) [file pone.0309124.s002.zip › Data Sheet/Additional file 1 Scatter plot figure/R20 Cognitive function on walking pace.tif]

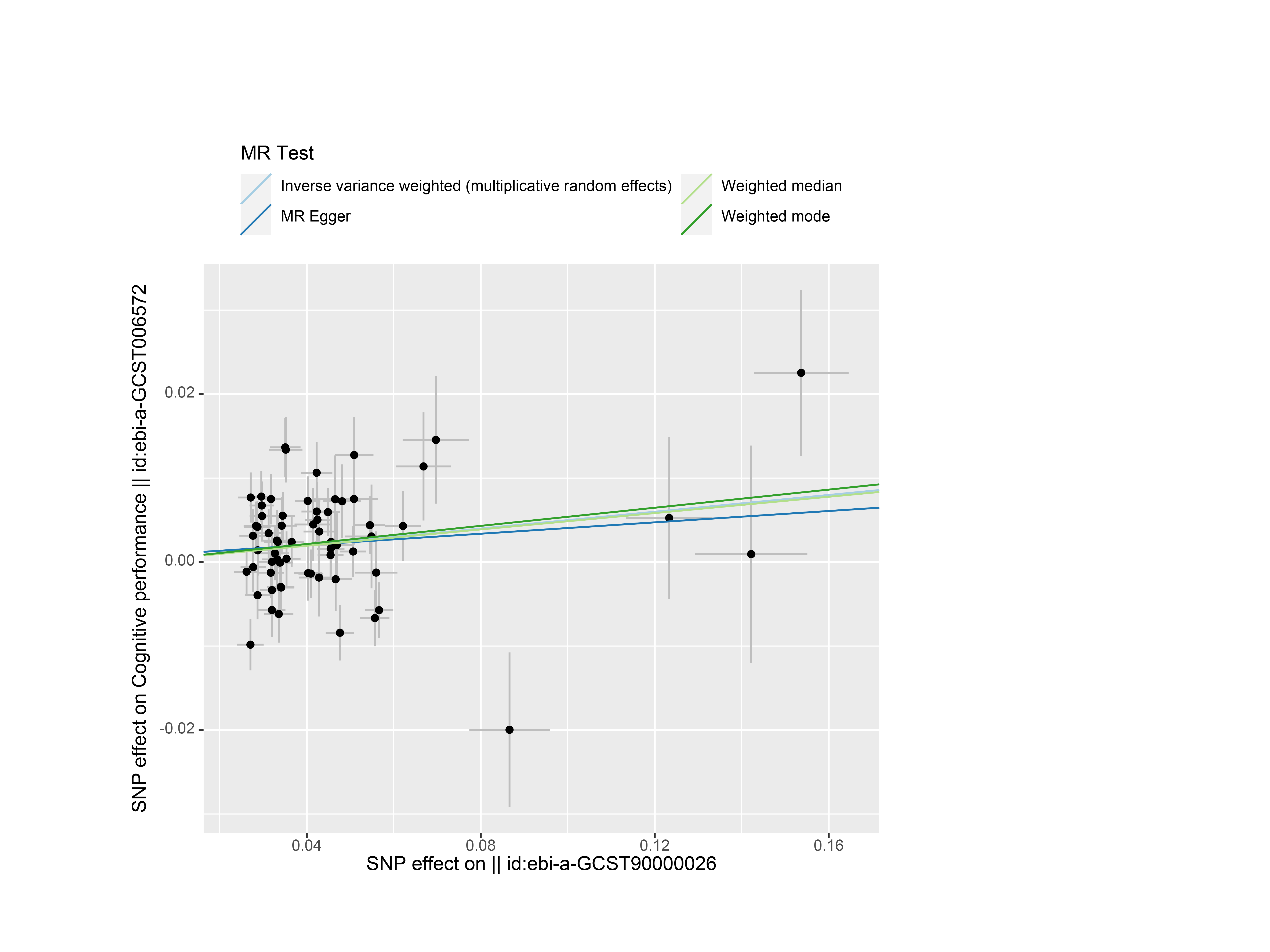

Supplement: S1 Data — (ZIP) [file pone.0309124.s002.zip › Data Sheet/Additional file 1 Scatter plot figure/R3 ALM-M on cognitive performance.tif]

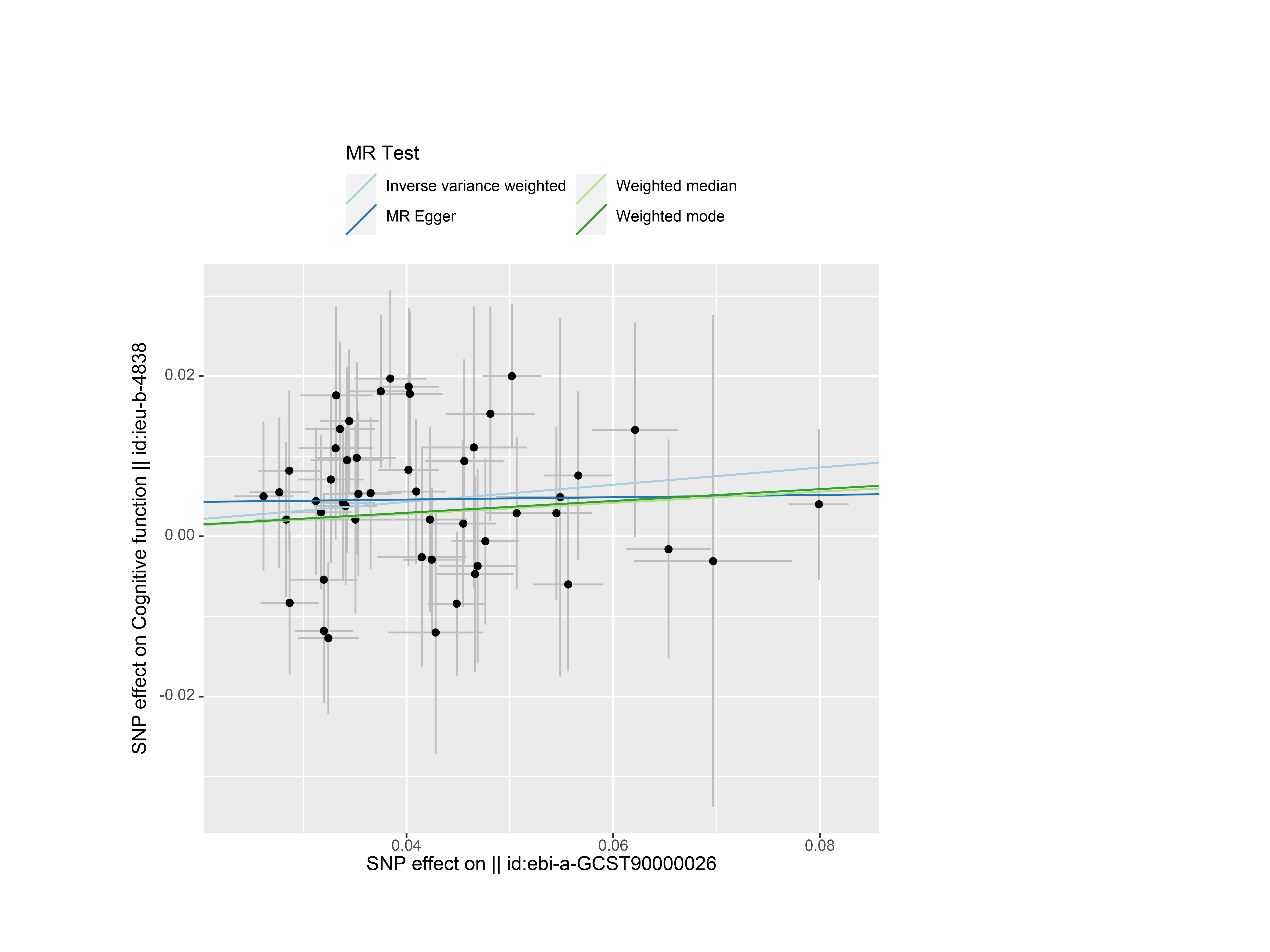

Supplement: S1 Data — (ZIP) [file pone.0309124.s002.zip › Data Sheet/Additional file 1 Scatter plot figure/R4 ALM-M on cognitive function.tif]

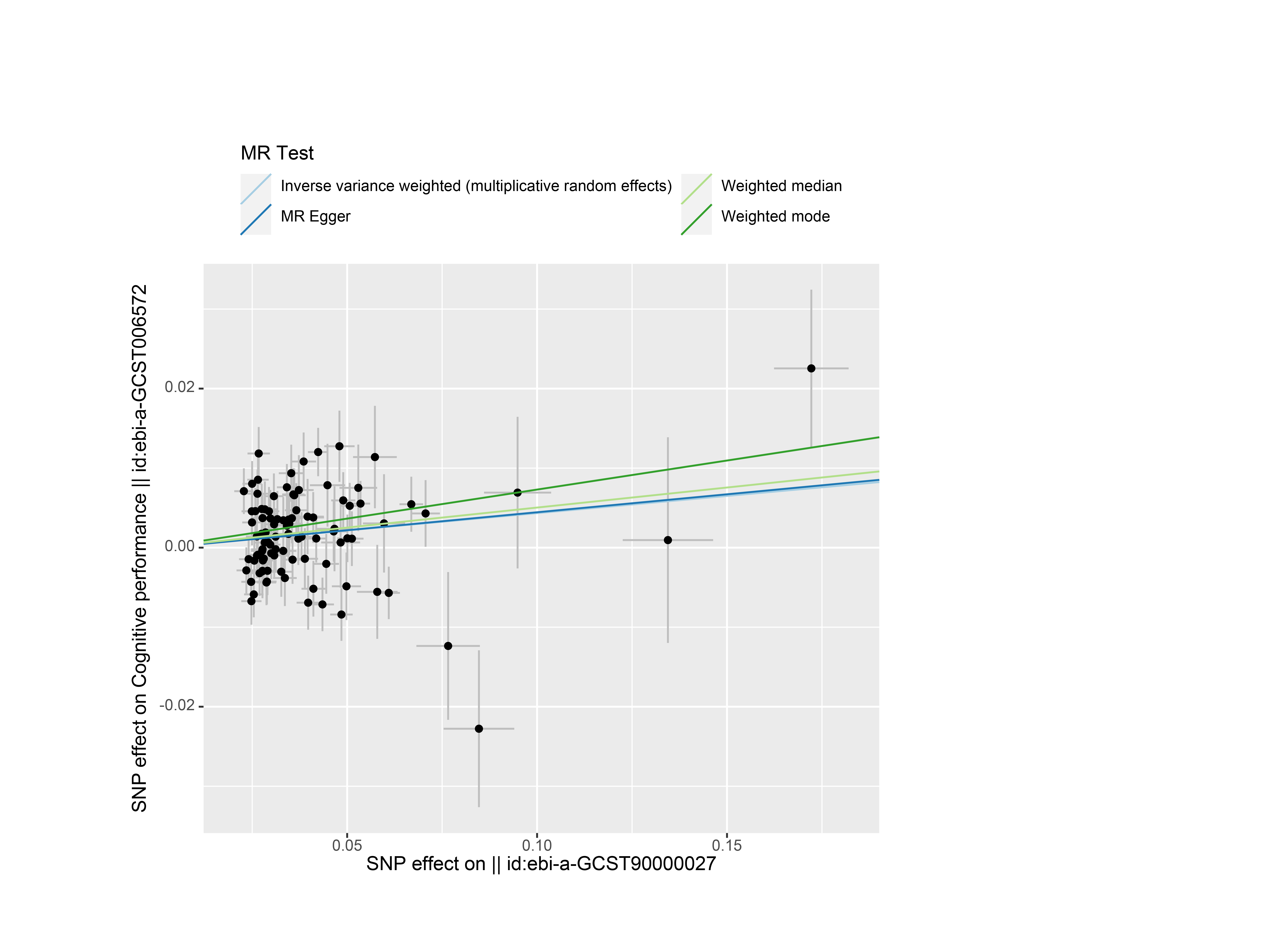

Supplement: S1 Data — (ZIP) [file pone.0309124.s002.zip › Data Sheet/Additional file 1 Scatter plot figure/R5 ALM-F on cognitive performance.tif]

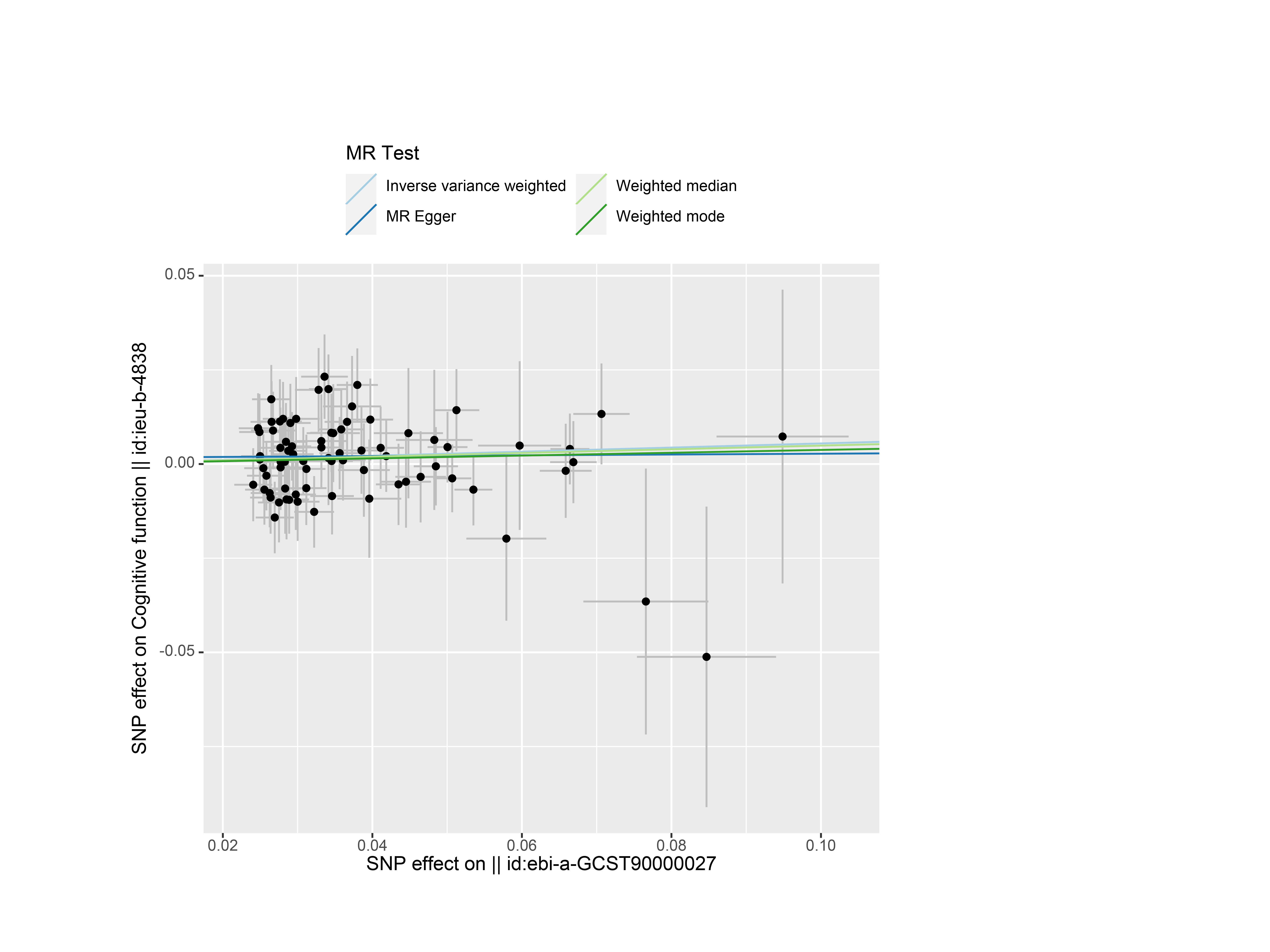

Supplement: S1 Data — (ZIP) [file pone.0309124.s002.zip › Data Sheet/Additional file 1 Scatter plot figure/R6 ALM-F on cognitive performance.tif]

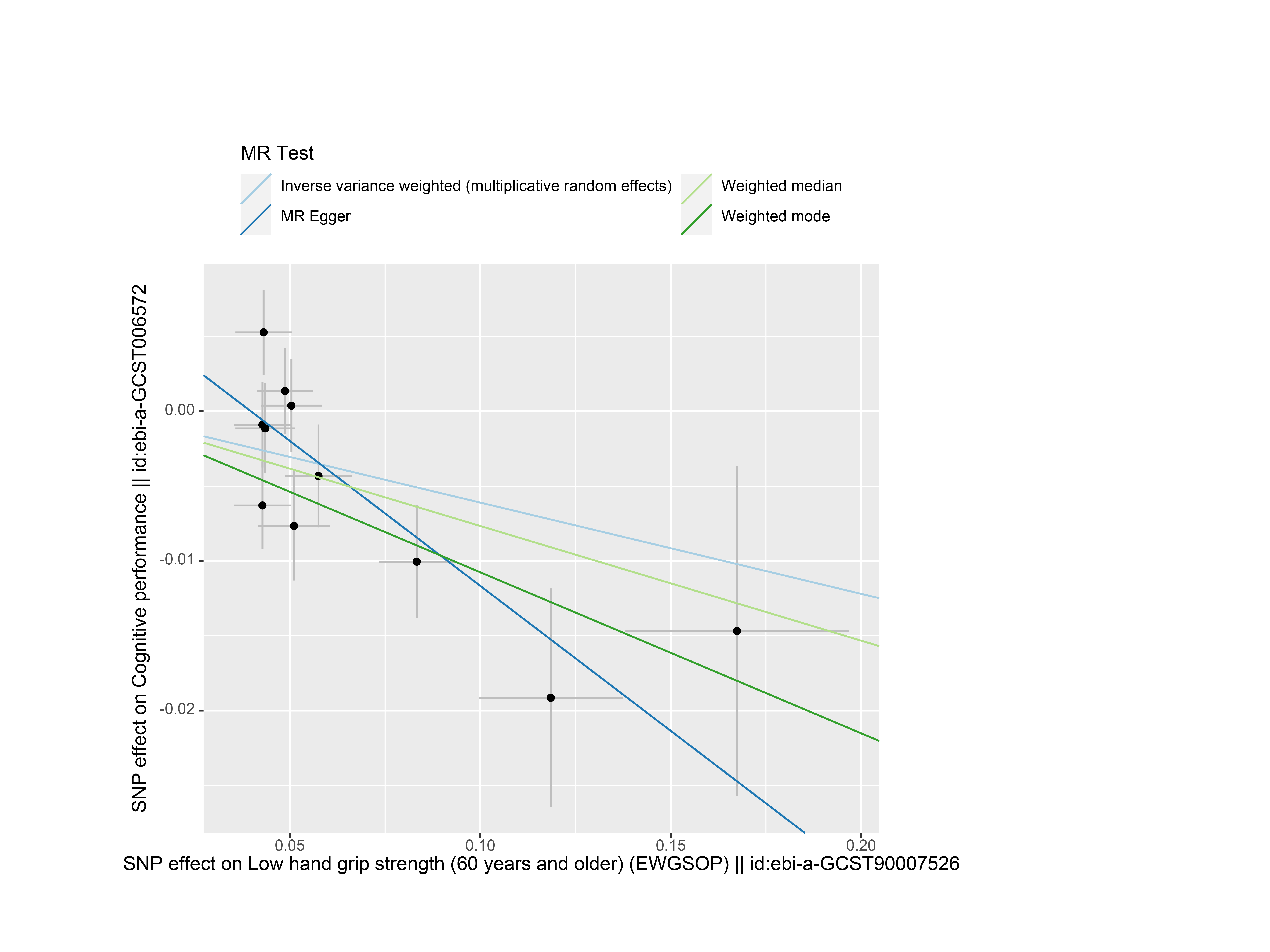

Supplement: S1 Data — (ZIP) [file pone.0309124.s002.zip › Data Sheet/Additional file 1 Scatter plot figure/R7 Low hand grip strength on cognitive performance.tif]

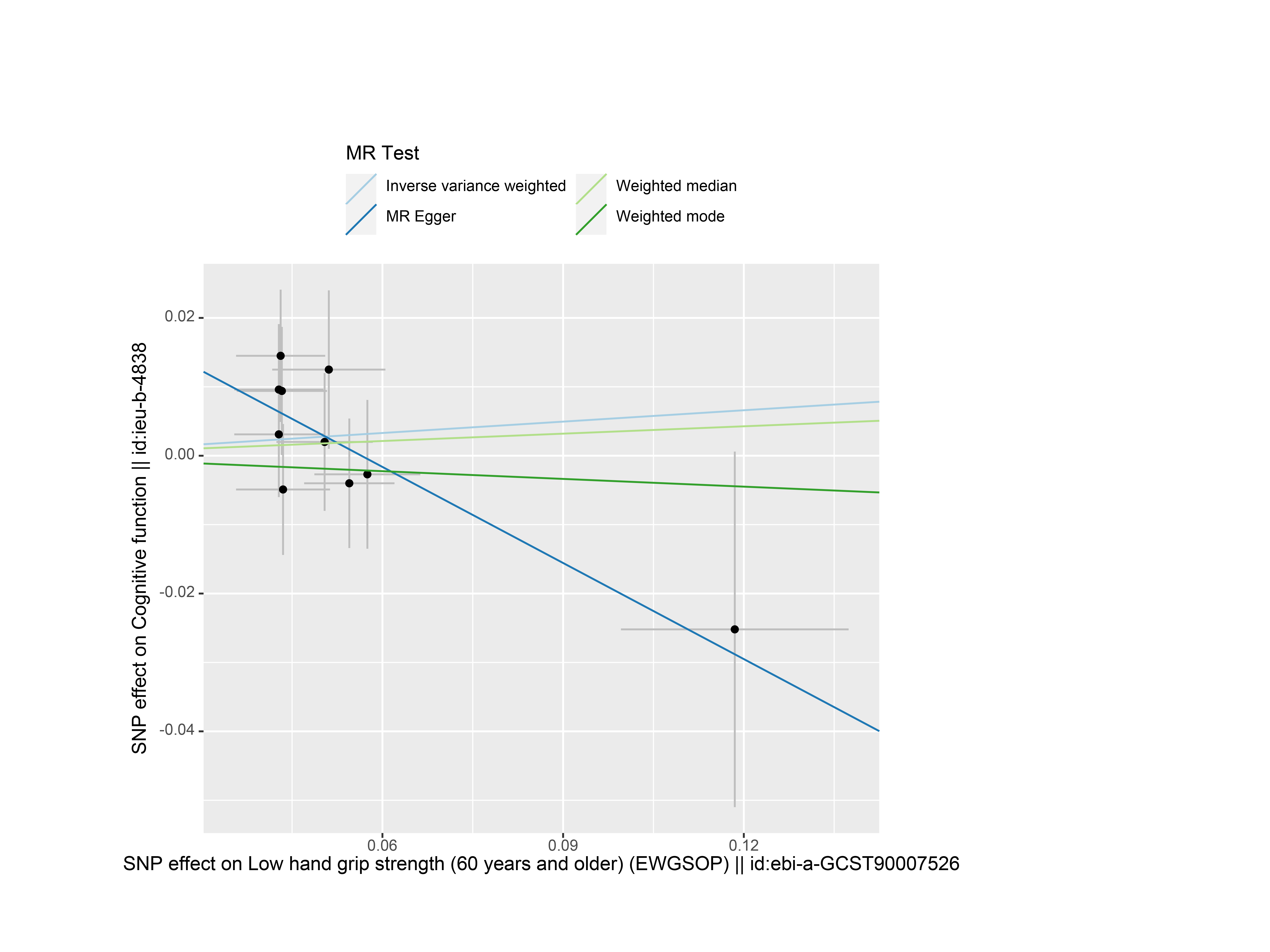

Supplement: S1 Data — (ZIP) [file pone.0309124.s002.zip › Data Sheet/Additional file 1 Scatter plot figure/R8 Low hand grip strength on cognitive function.tif]

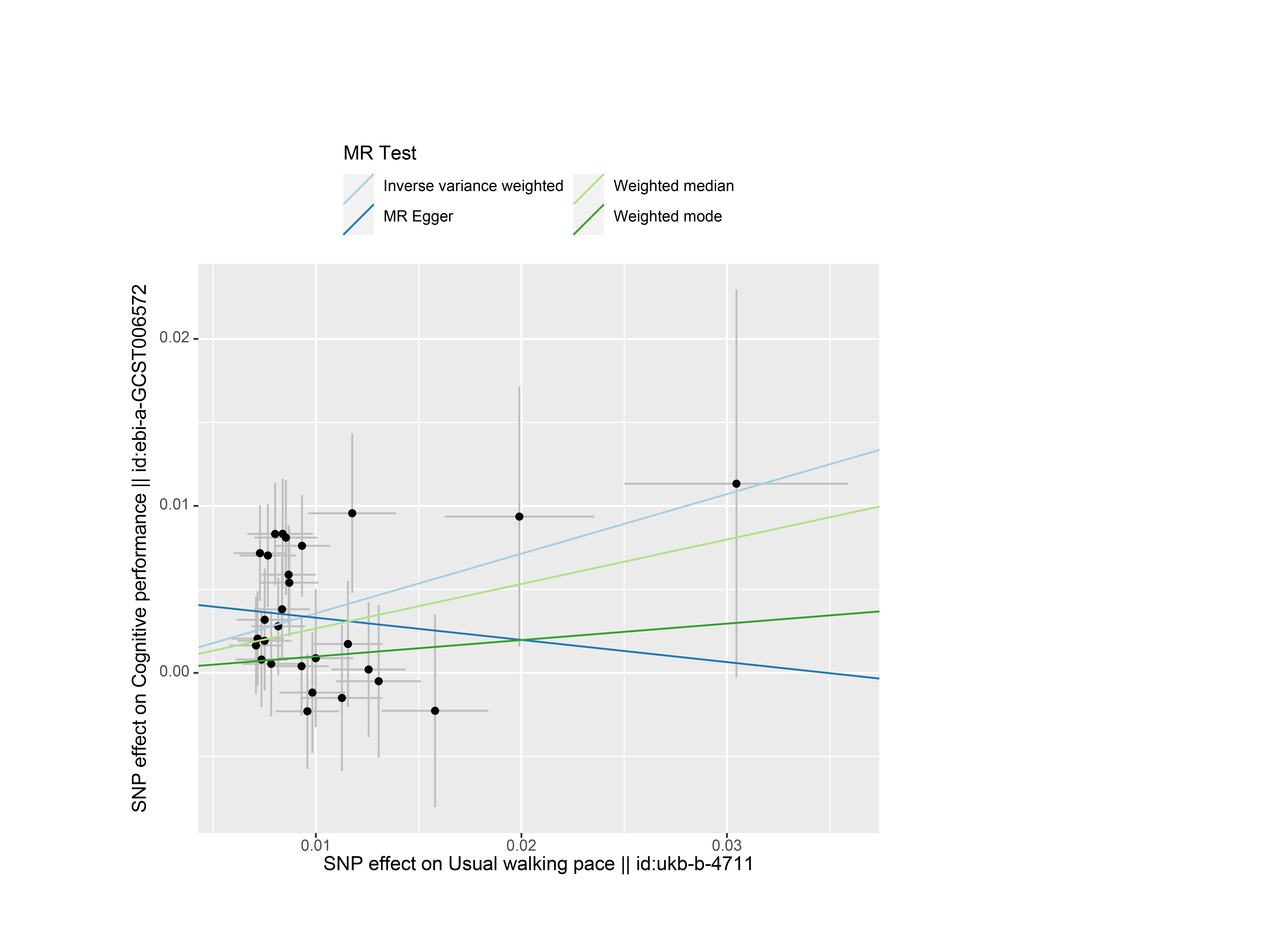

Supplement: S1 Data — (ZIP) [file pone.0309124.s002.zip › Data Sheet/Additional file 1 Scatter plot figure/R9 Walking pace on cognitive performance.tif]

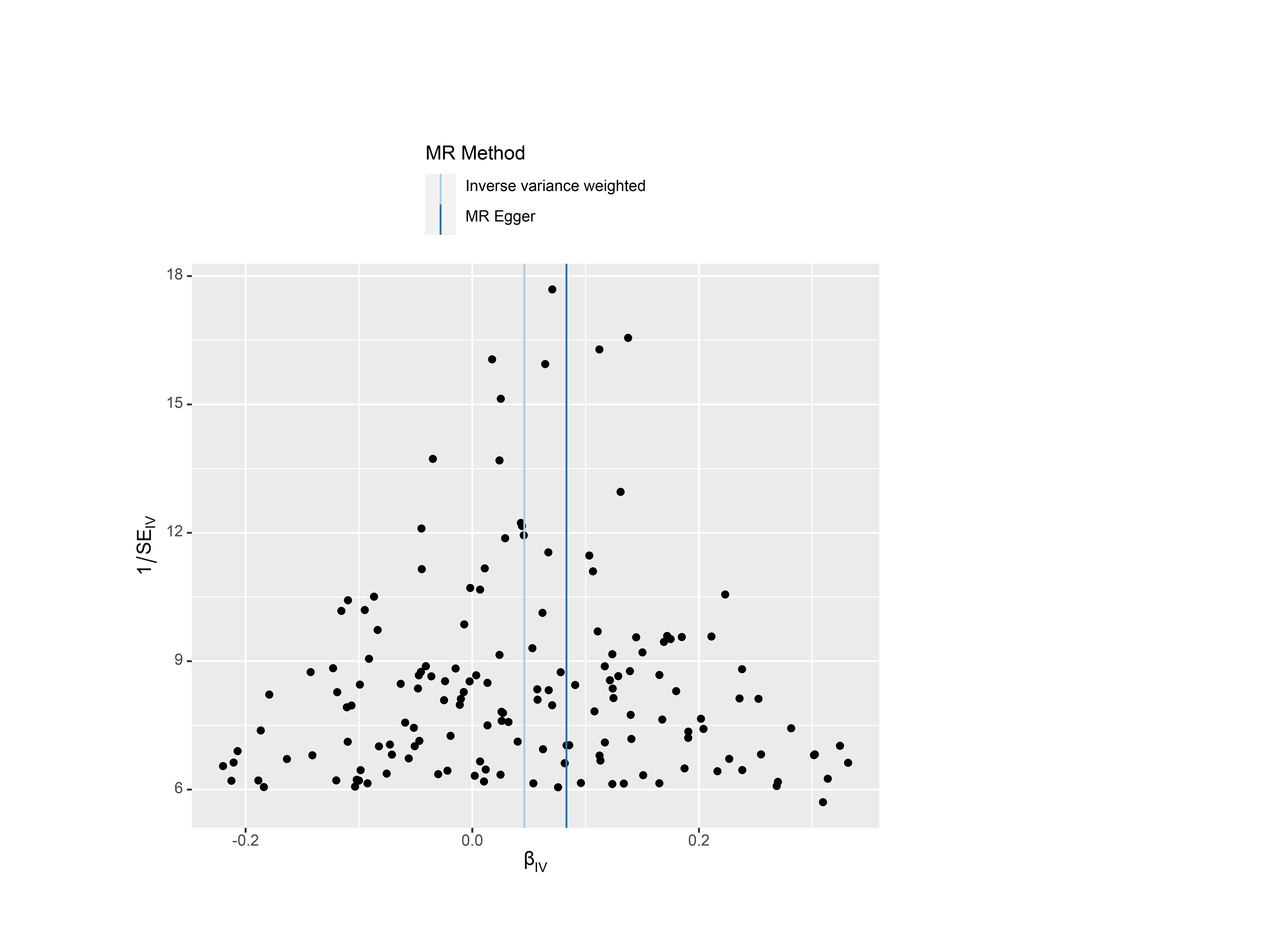

Supplement: S1 Data — (ZIP) [file pone.0309124.s002.zip › Data Sheet/Additional file 2 Funnel plot figure/S1 ALM on cognitive performance.tif]

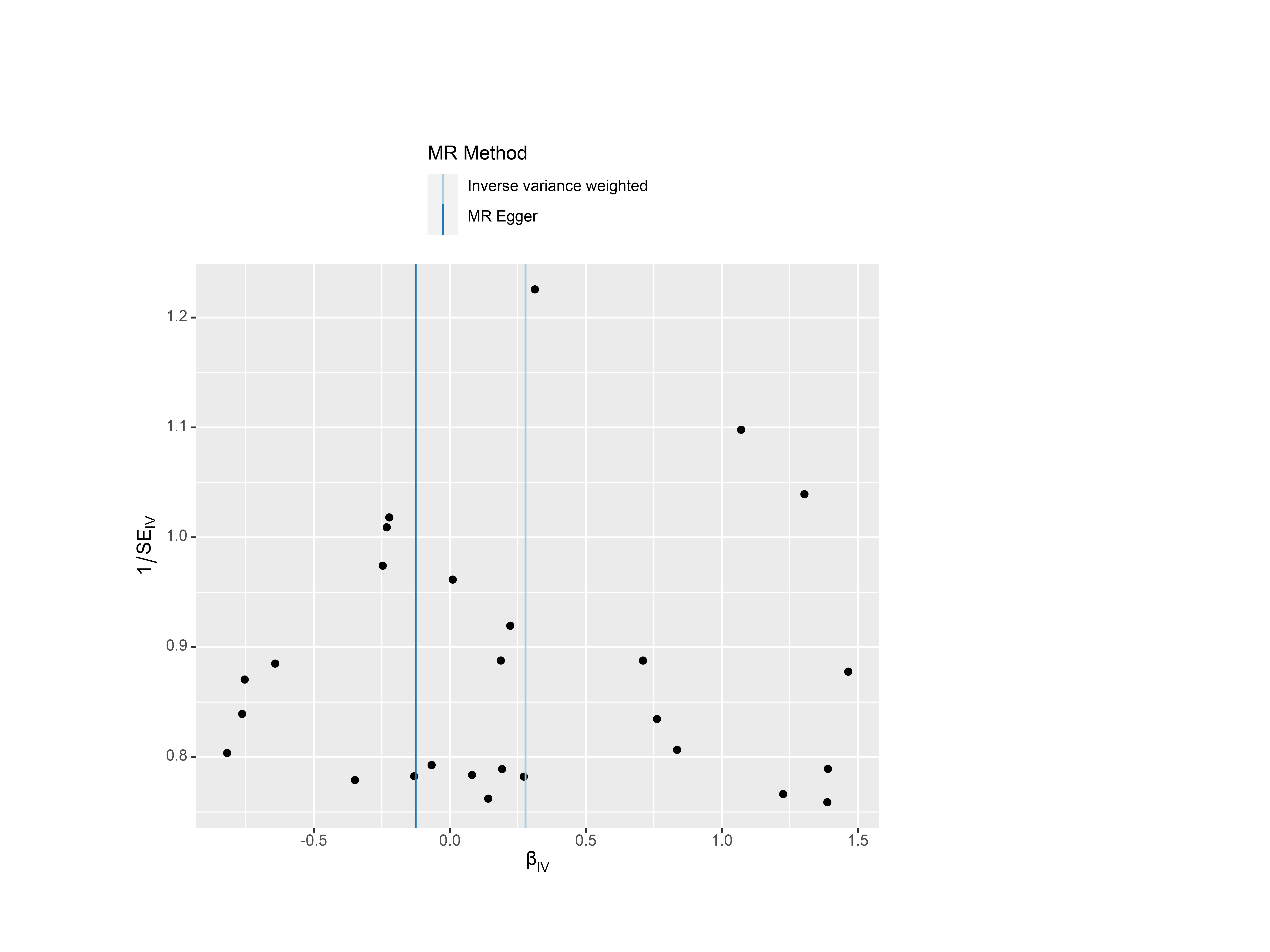

Supplement: S1 Data — (ZIP) [file pone.0309124.s002.zip › Data Sheet/Additional file 2 Funnel plot figure/S10 Walking pace on cognitive function.tif]

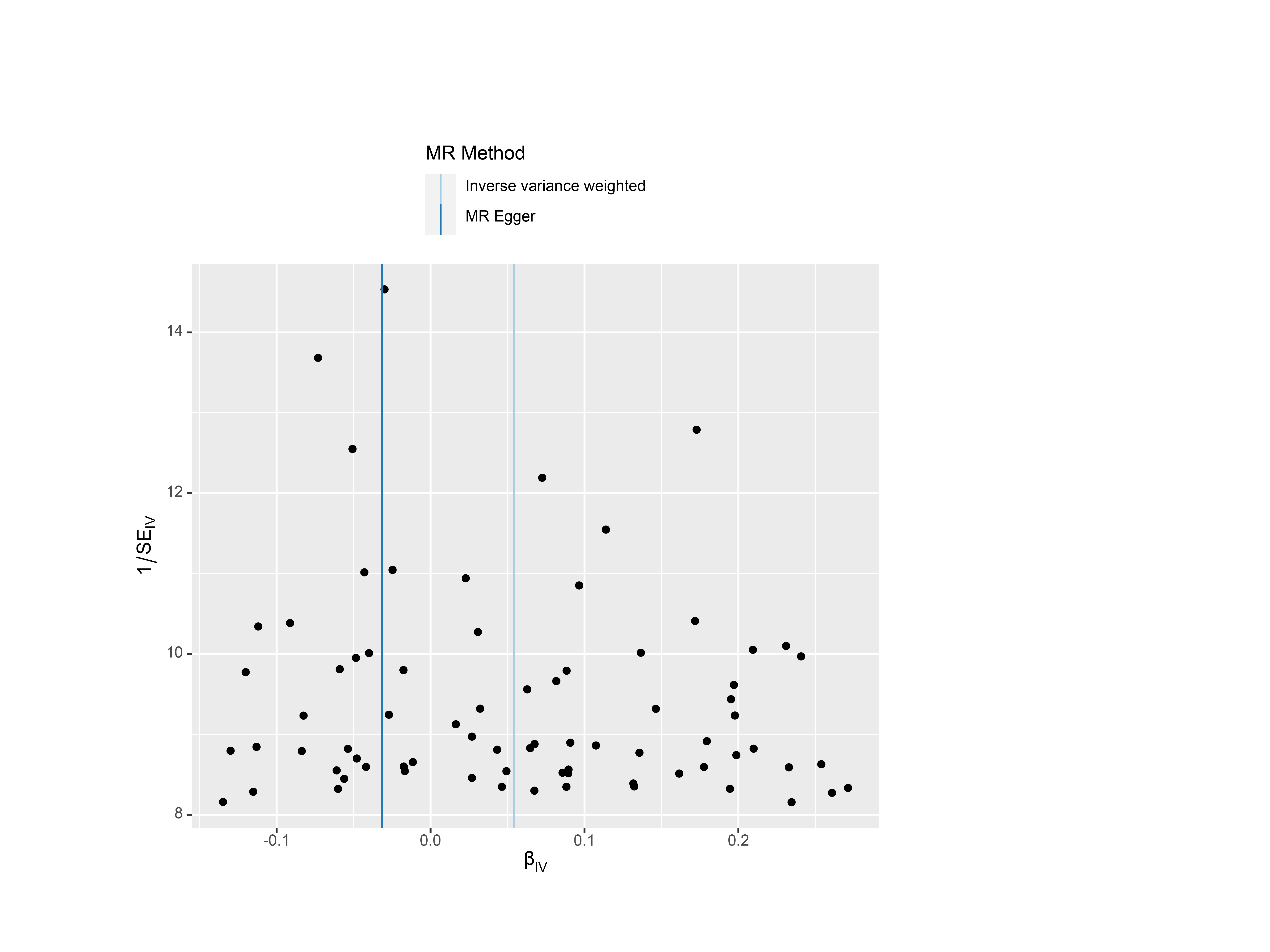

Supplement: S1 Data — (ZIP) [file pone.0309124.s002.zip › Data Sheet/Additional file 2 Funnel plot figure/S11 Cognitive performance on ALM.tif]

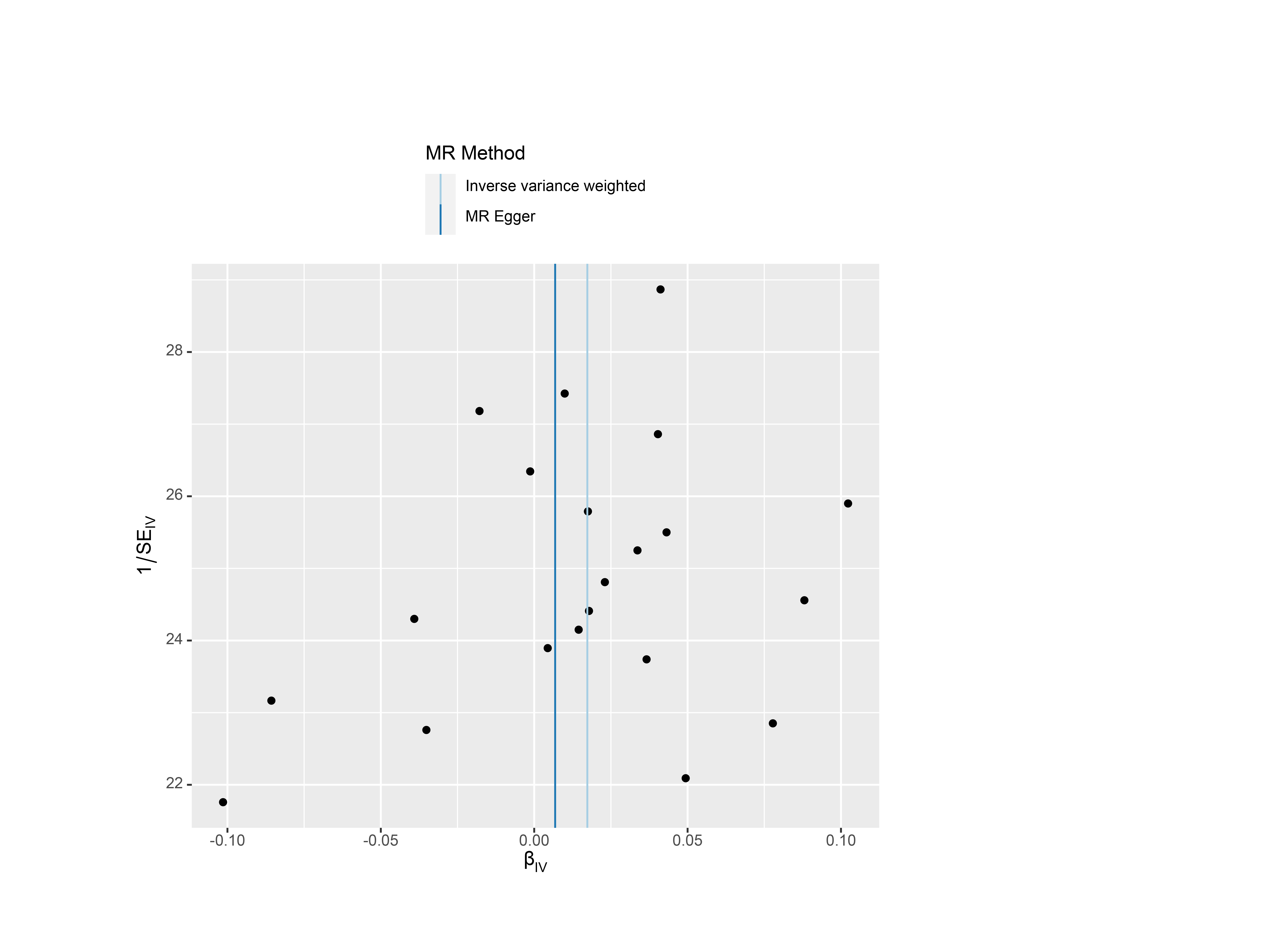

Supplement: S1 Data — (ZIP) [file pone.0309124.s002.zip › Data Sheet/Additional file 2 Funnel plot figure/S12 Cognitive function on ALM.tif]

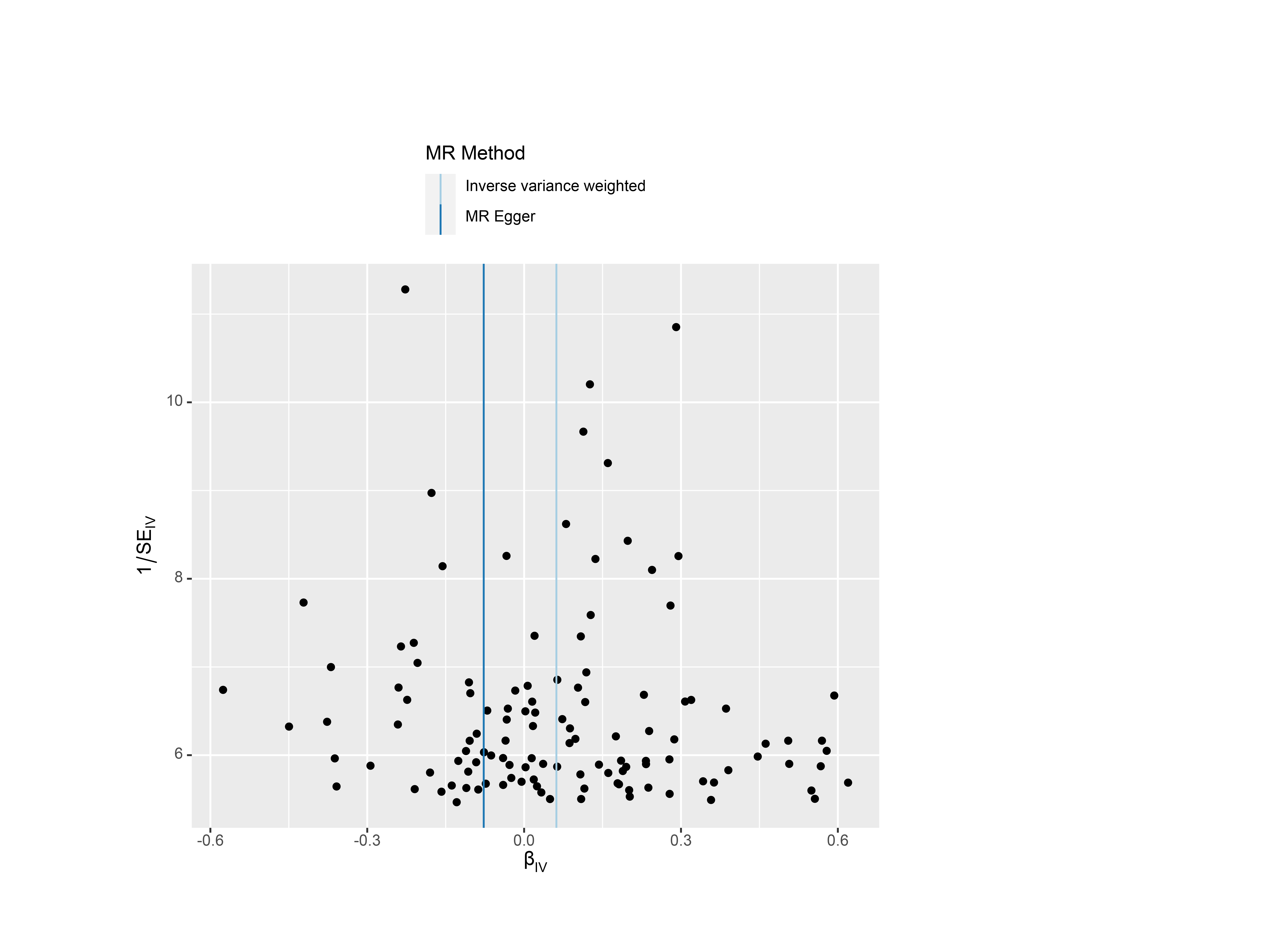

Supplement: S1 Data — (ZIP) [file pone.0309124.s002.zip › Data Sheet/Additional file 2 Funnel plot figure/S13 Cognitive performance on ALM-M.tif]

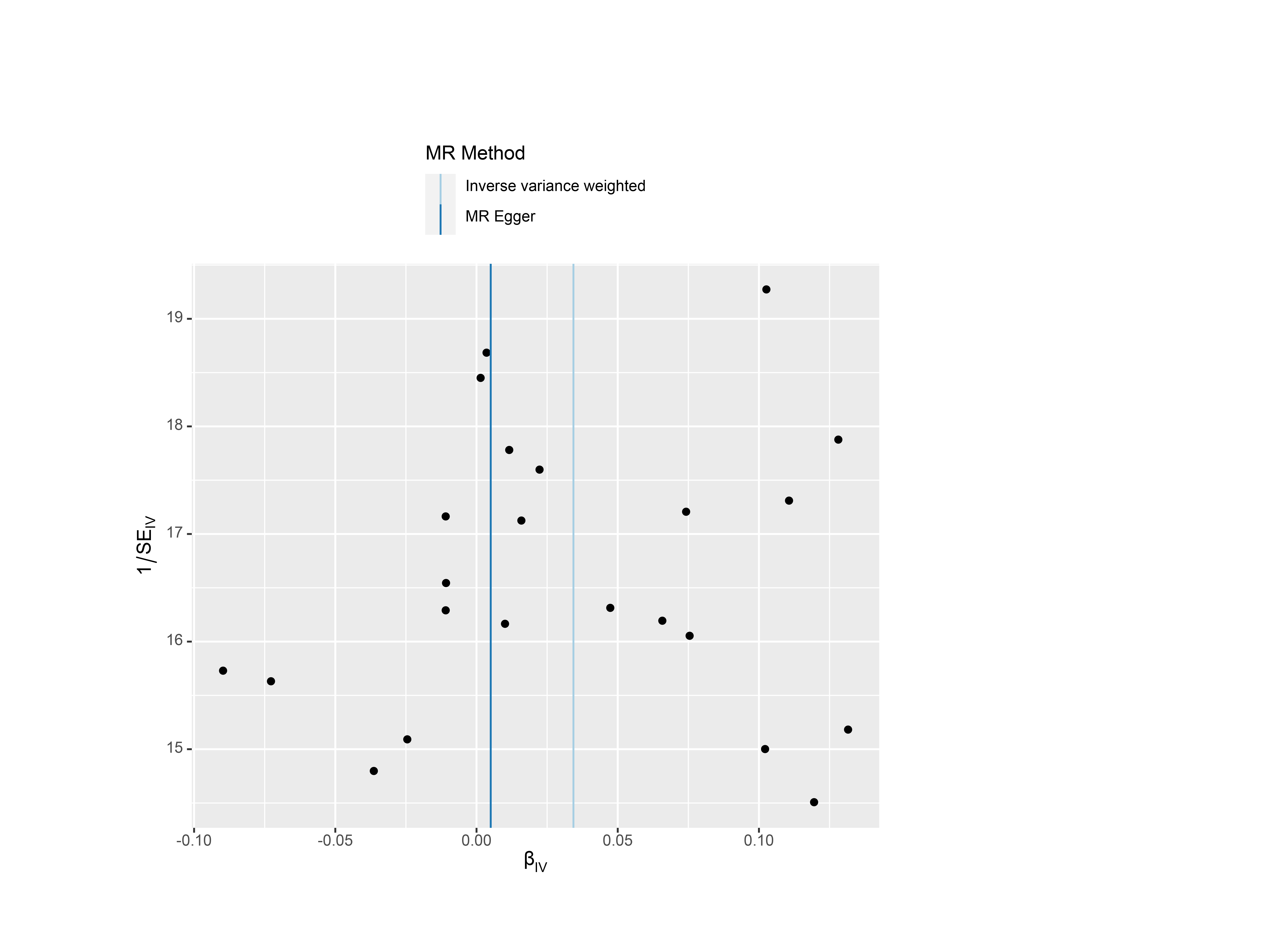

Supplement: S1 Data — (ZIP) [file pone.0309124.s002.zip › Data Sheet/Additional file 2 Funnel plot figure/S14 Cognitive function on ALM-M.tif]

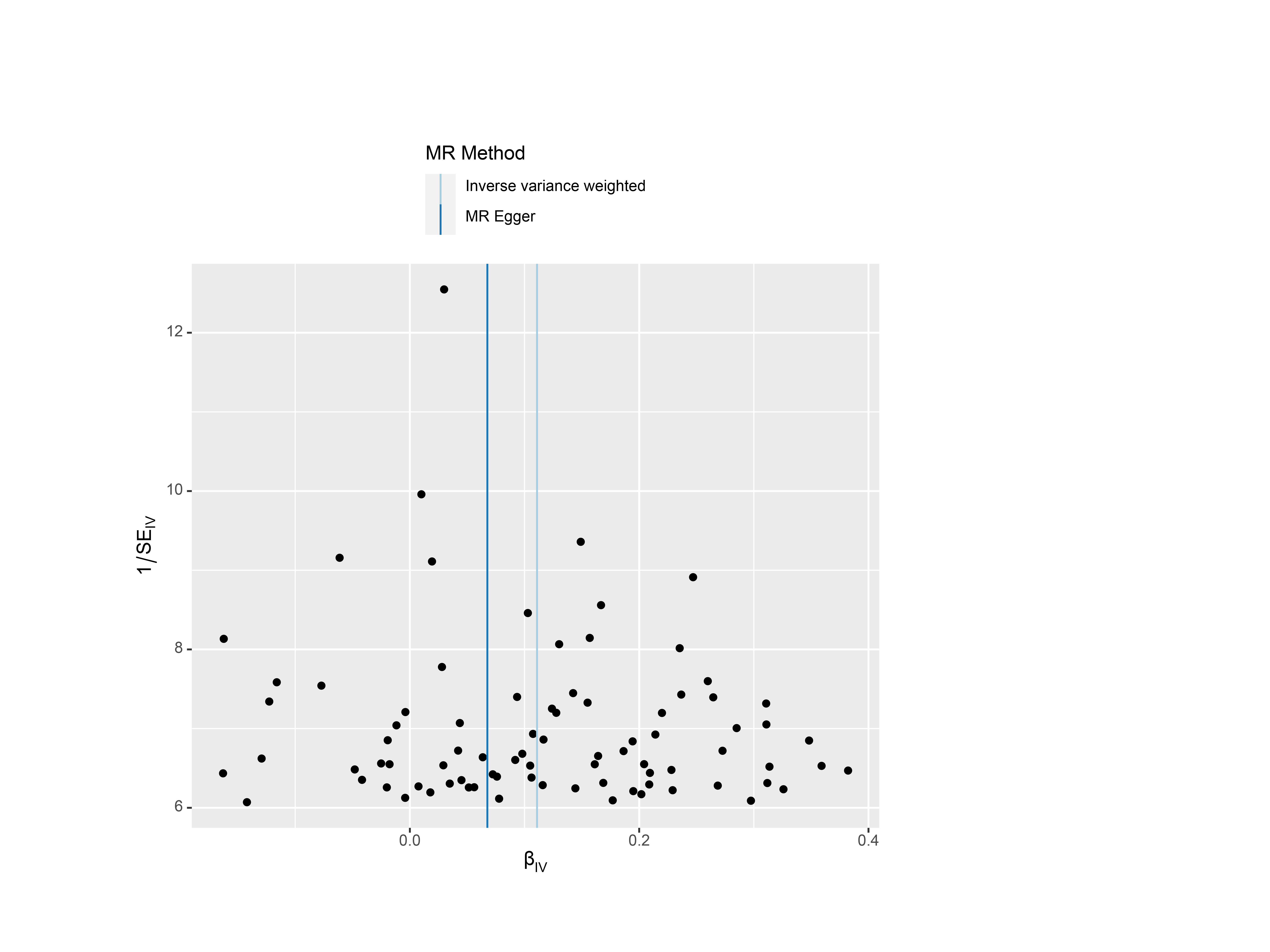

Supplement: S1 Data — (ZIP) [file pone.0309124.s002.zip › Data Sheet/Additional file 2 Funnel plot figure/S15 Cognitive performance on ALM-F.tif]

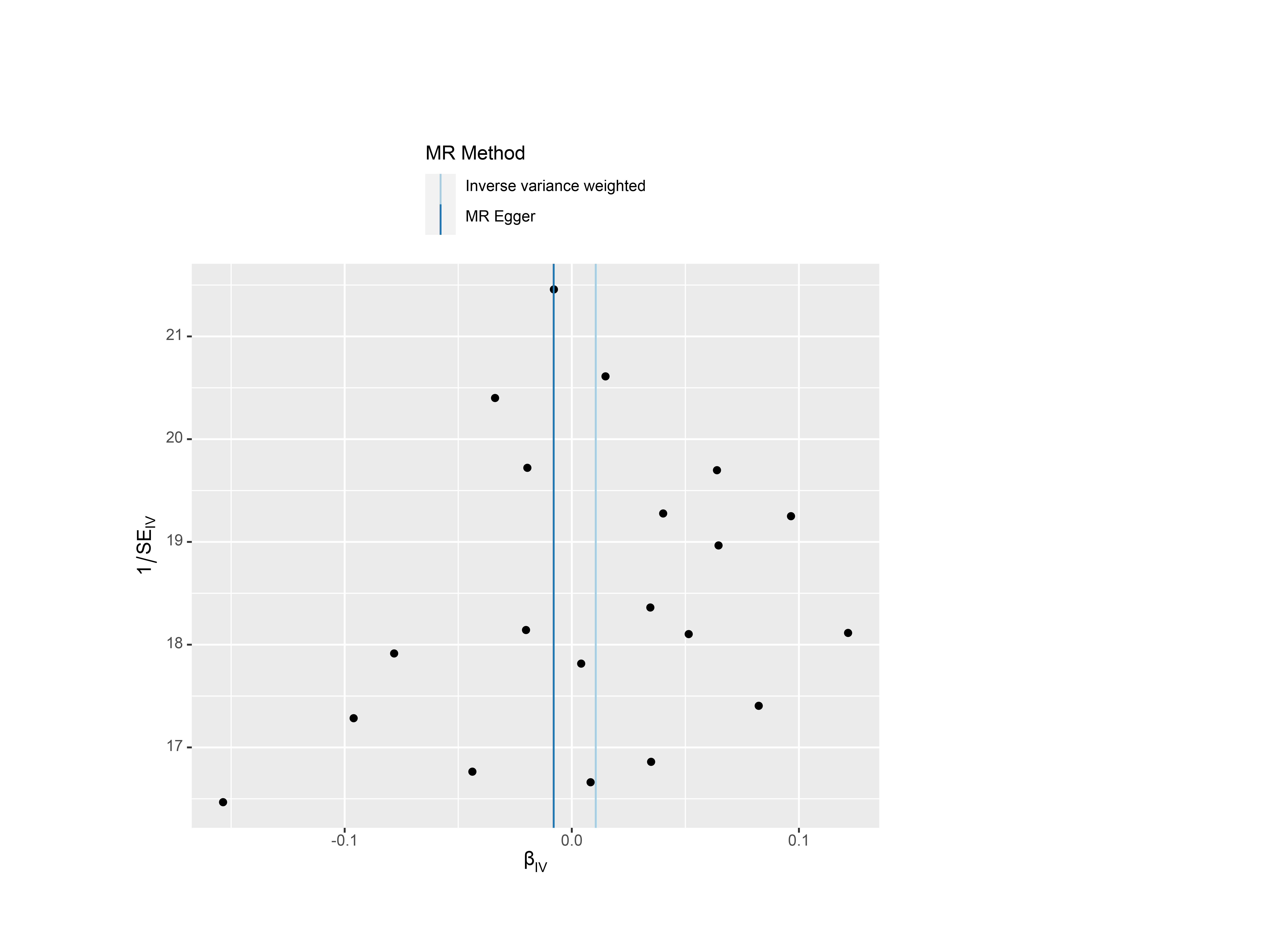

Supplement: S1 Data — (ZIP) [file pone.0309124.s002.zip › Data Sheet/Additional file 2 Funnel plot figure/S16 Cognitive function on ALM-F.tif]

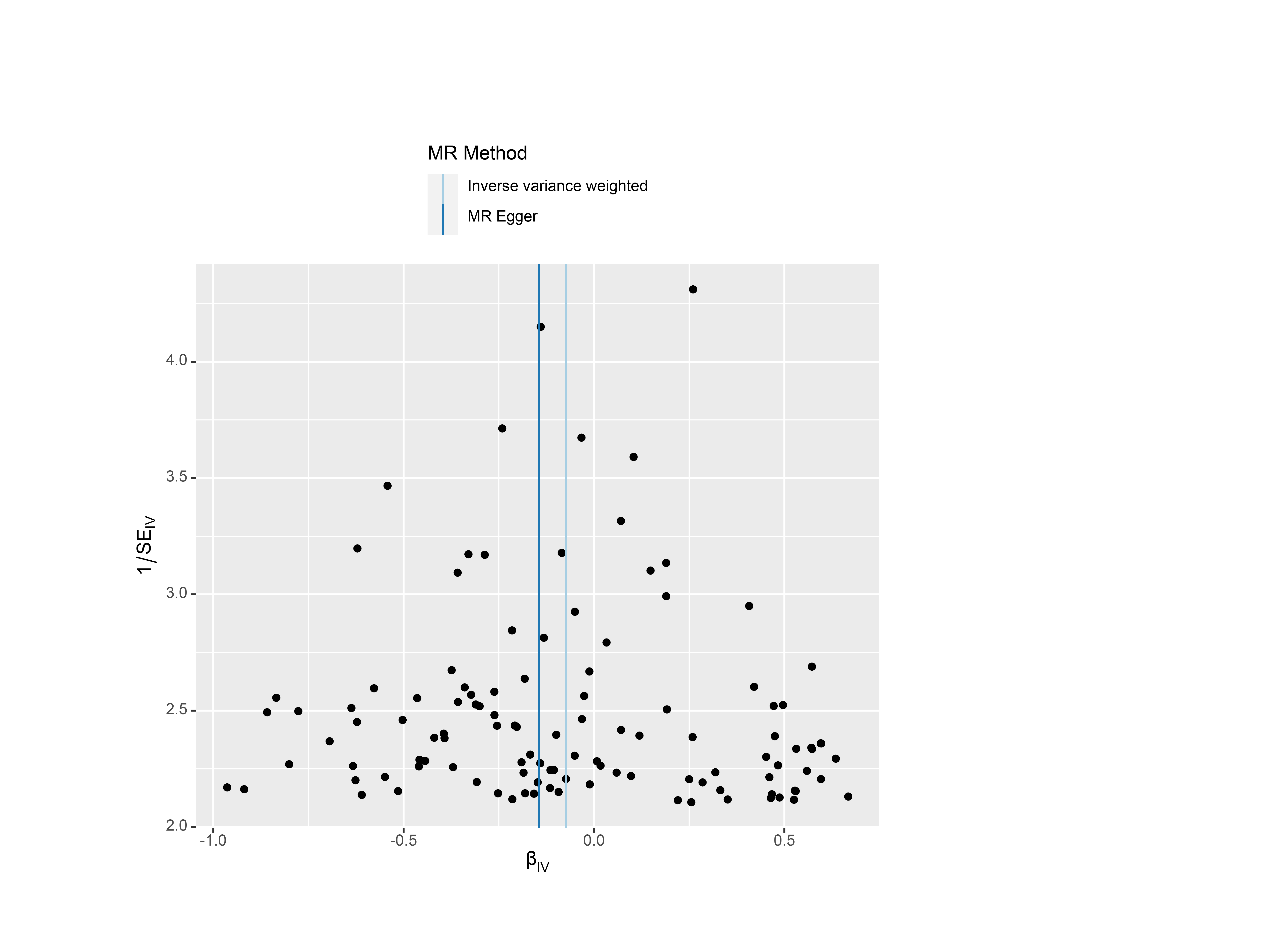

Supplement: S1 Data — (ZIP) [file pone.0309124.s002.zip › Data Sheet/Additional file 2 Funnel plot figure/S17 Cognitive performance on low hand grip strength.tif]

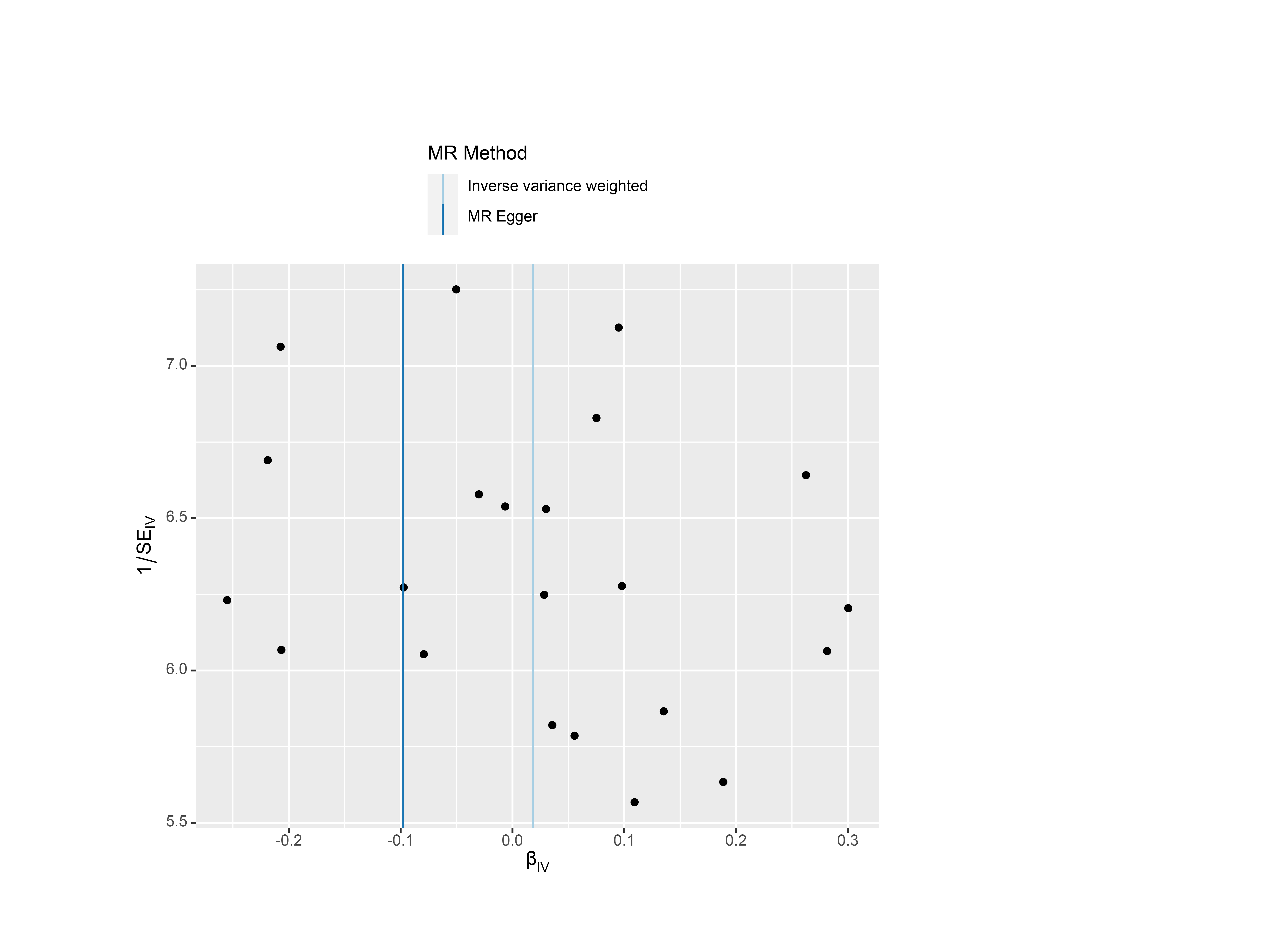

Supplement: S1 Data — (ZIP) [file pone.0309124.s002.zip › Data Sheet/Additional file 2 Funnel plot figure/S18 Cognitive function on low hand grip strength.tif]

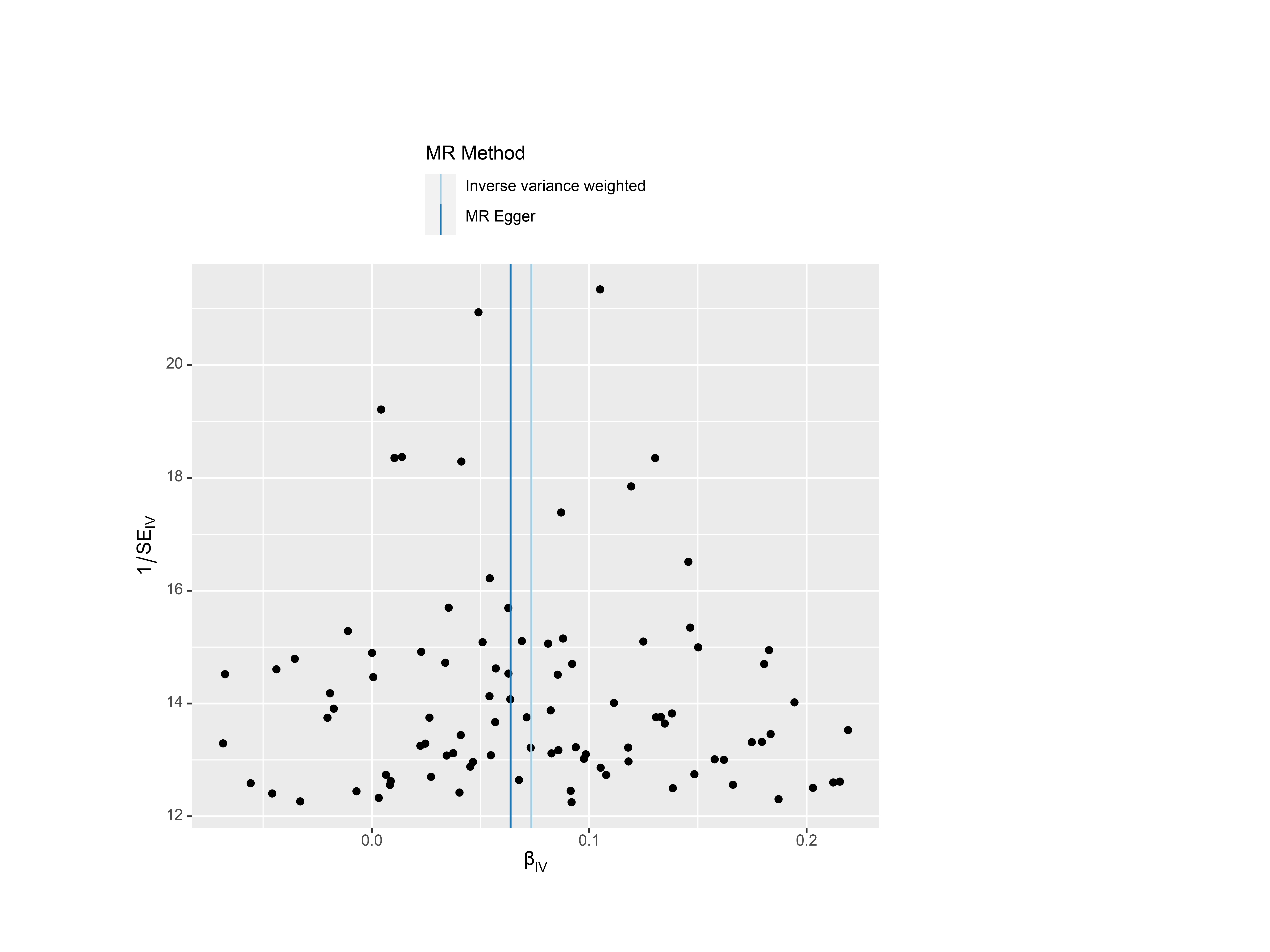

Supplement: S1 Data — (ZIP) [file pone.0309124.s002.zip › Data Sheet/Additional file 2 Funnel plot figure/S19 Cognitive performance on walking pace.tif]

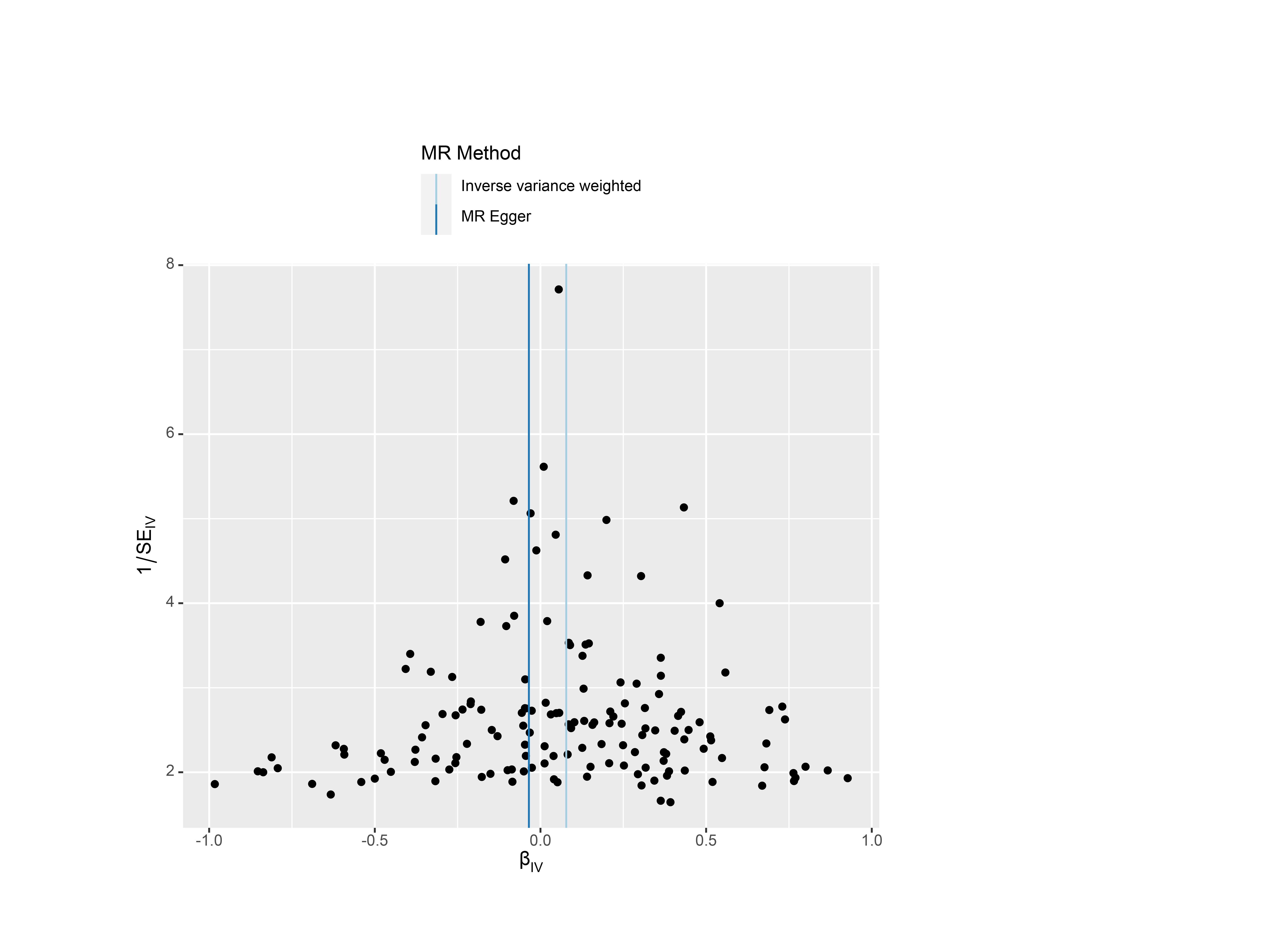

Supplement: S1 Data — (ZIP) [file pone.0309124.s002.zip › Data Sheet/Additional file 2 Funnel plot figure/S2 ALM on cognitive function.tif]

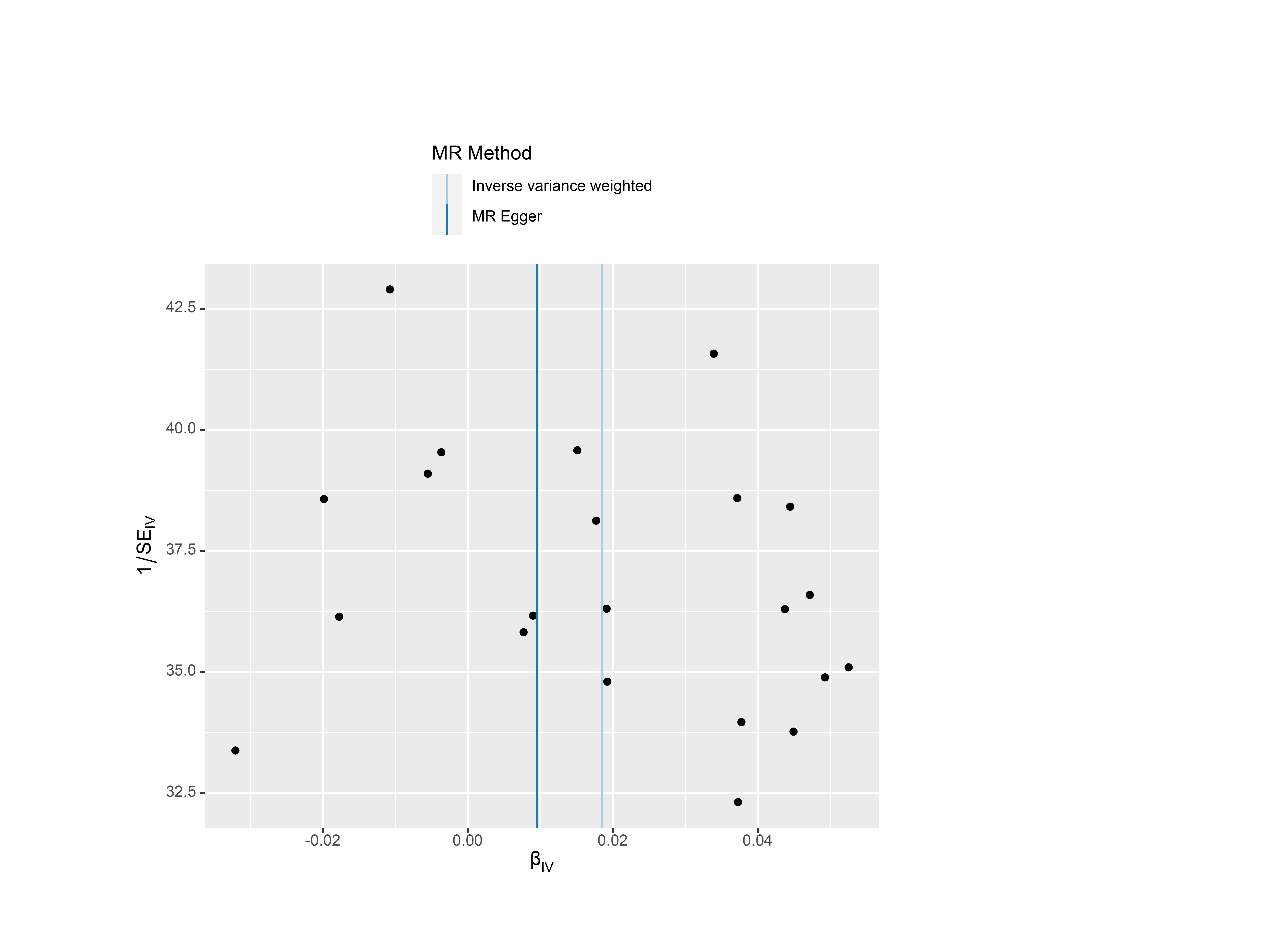

Supplement: S1 Data — (ZIP) [file pone.0309124.s002.zip › Data Sheet/Additional file 2 Funnel plot figure/S20 Cognitive function on walking pace.tif]

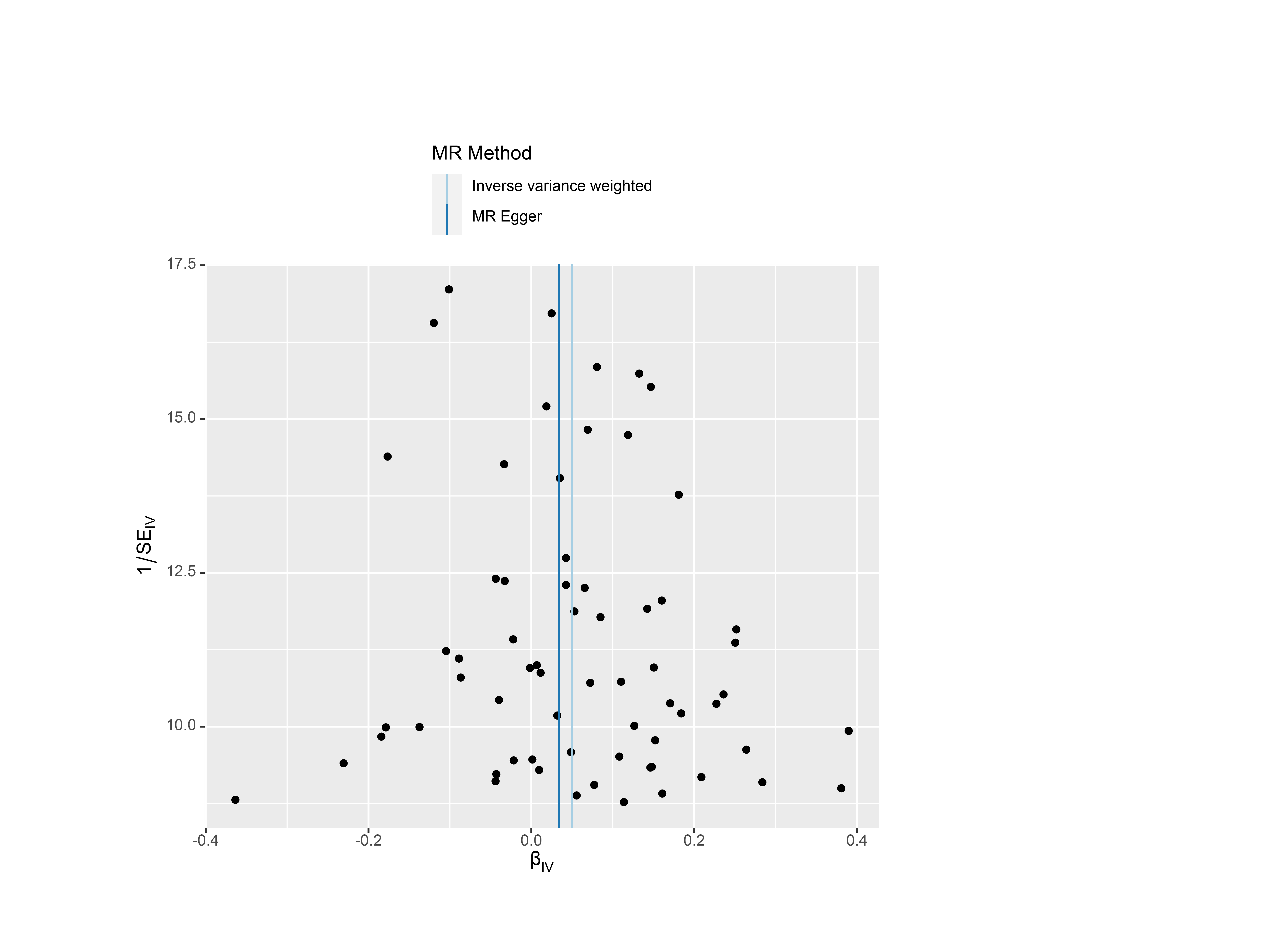

Supplement: S1 Data — (ZIP) [file pone.0309124.s002.zip › Data Sheet/Additional file 2 Funnel plot figure/S3 ALM-M on cognitive performance.tif]

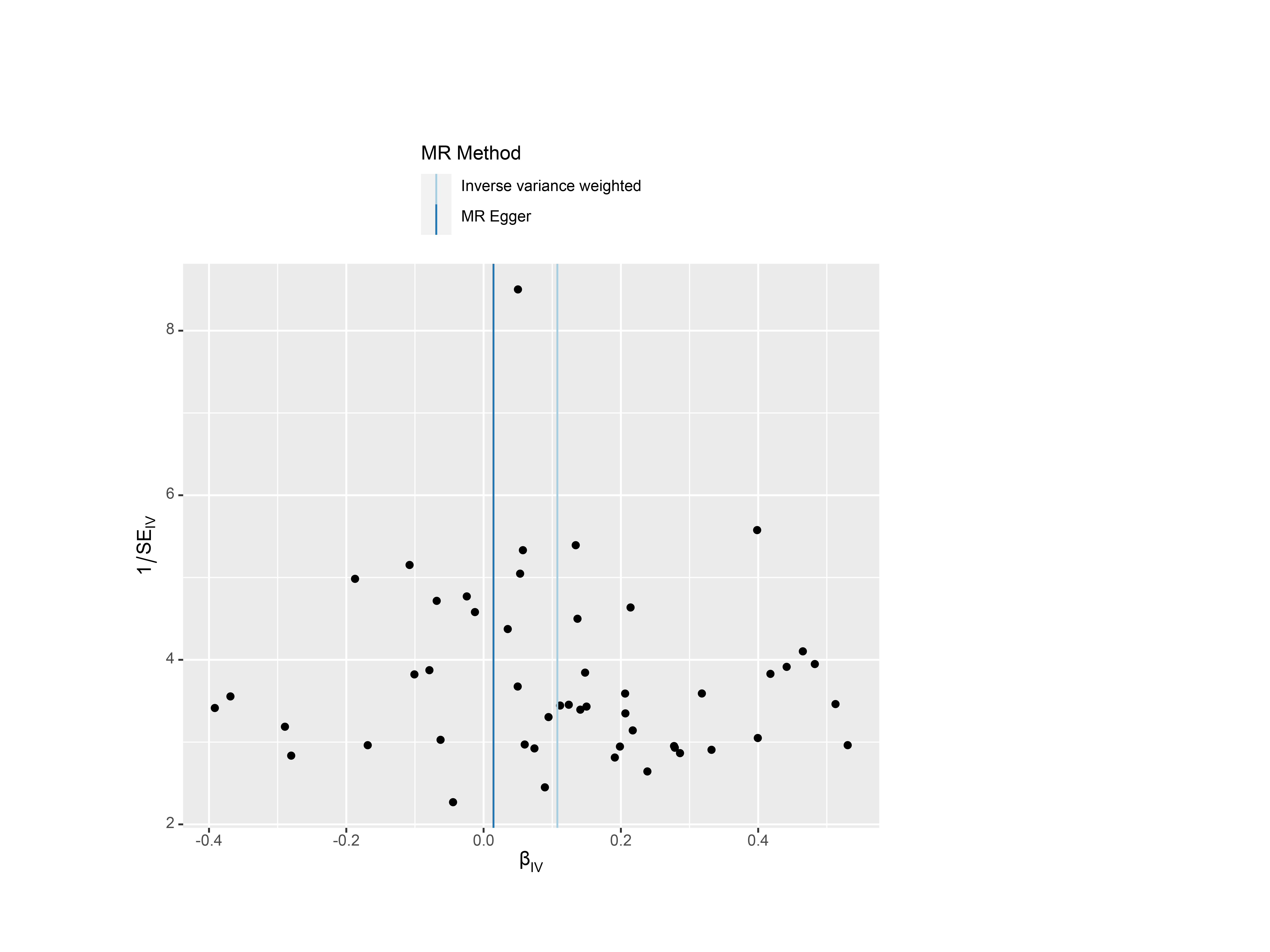

Supplement: S1 Data — (ZIP) [file pone.0309124.s002.zip › Data Sheet/Additional file 2 Funnel plot figure/S4 ALM-M on cognitive function.tif]

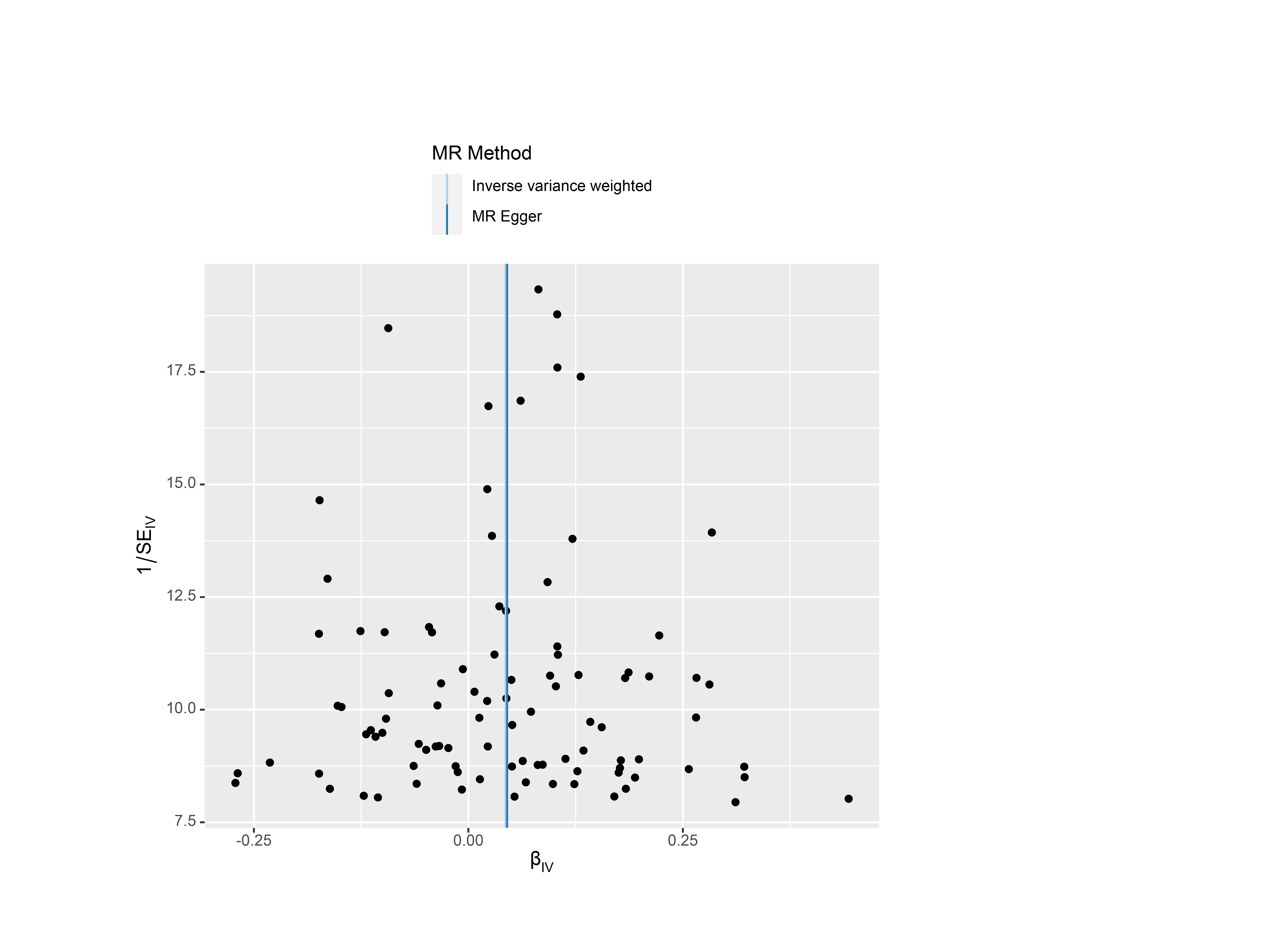

Supplement: S1 Data — (ZIP) [file pone.0309124.s002.zip › Data Sheet/Additional file 2 Funnel plot figure/S5 ALM-F on cognitive performance.tif]

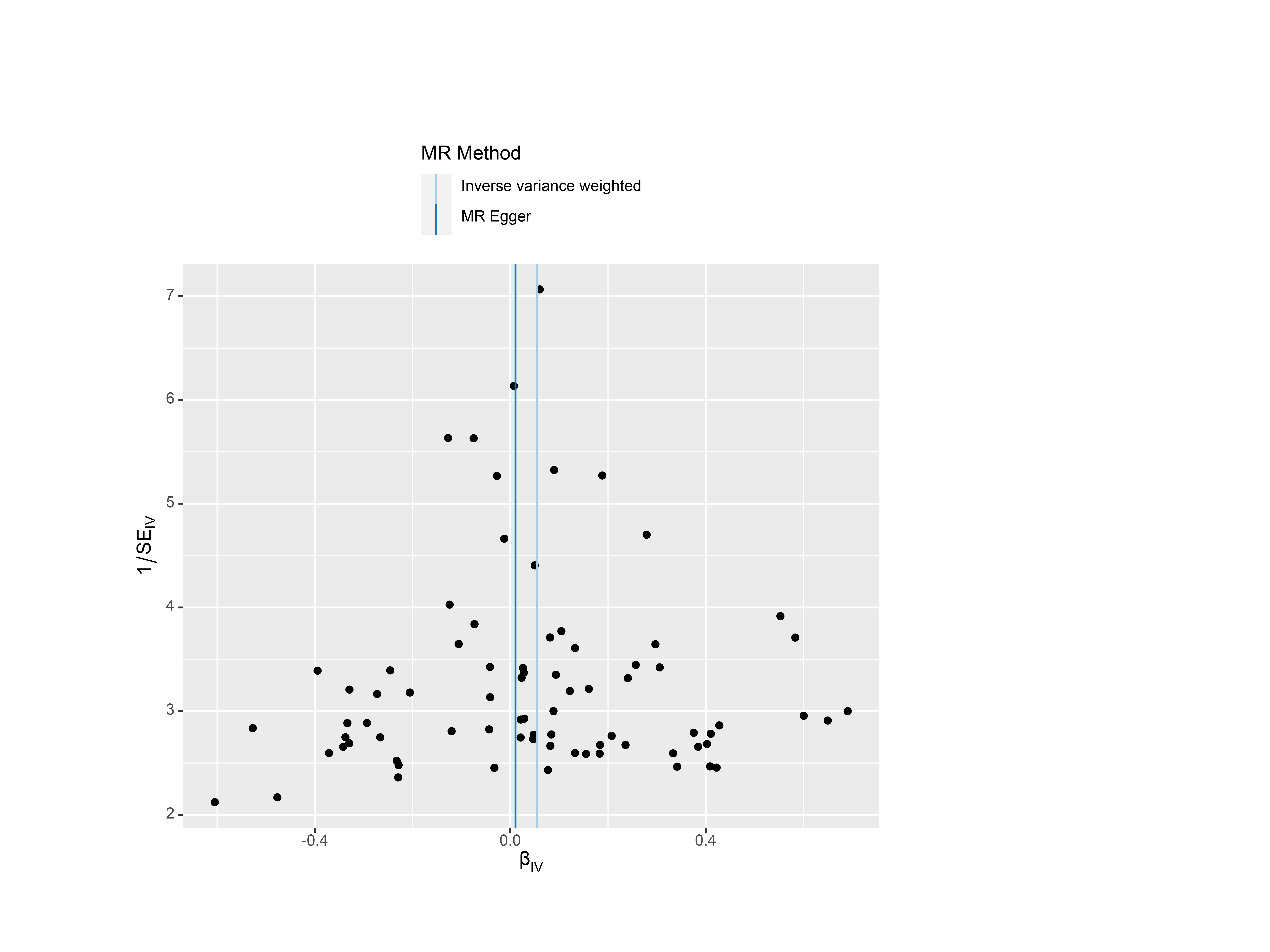

Supplement: S1 Data — (ZIP) [file pone.0309124.s002.zip › Data Sheet/Additional file 2 Funnel plot figure/S6 ALM-F on cognitive function.tif]

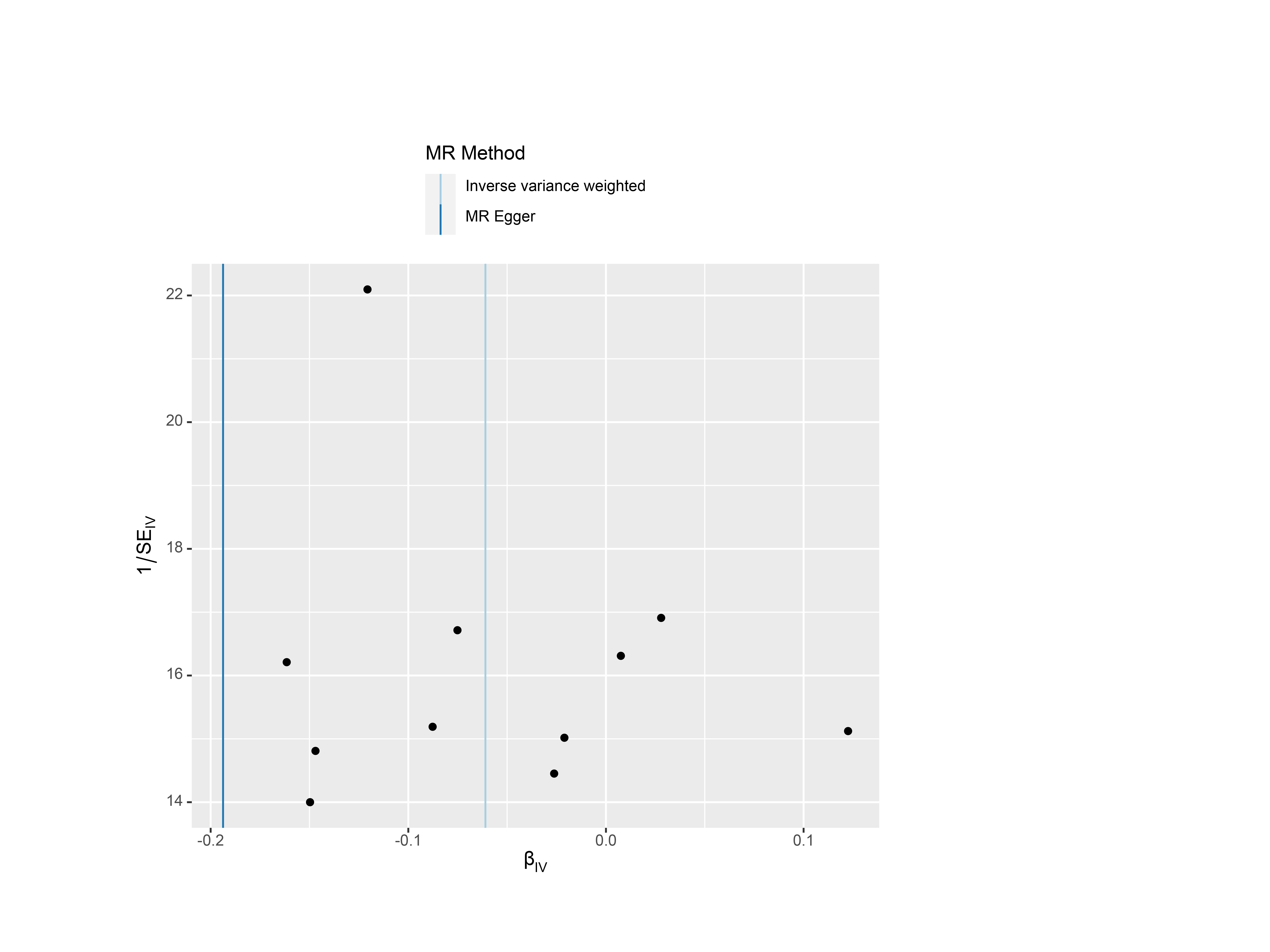

Supplement: S1 Data — (ZIP) [file pone.0309124.s002.zip › Data Sheet/Additional file 2 Funnel plot figure/S7 Low hand grip strength on cognitive performance.tif]

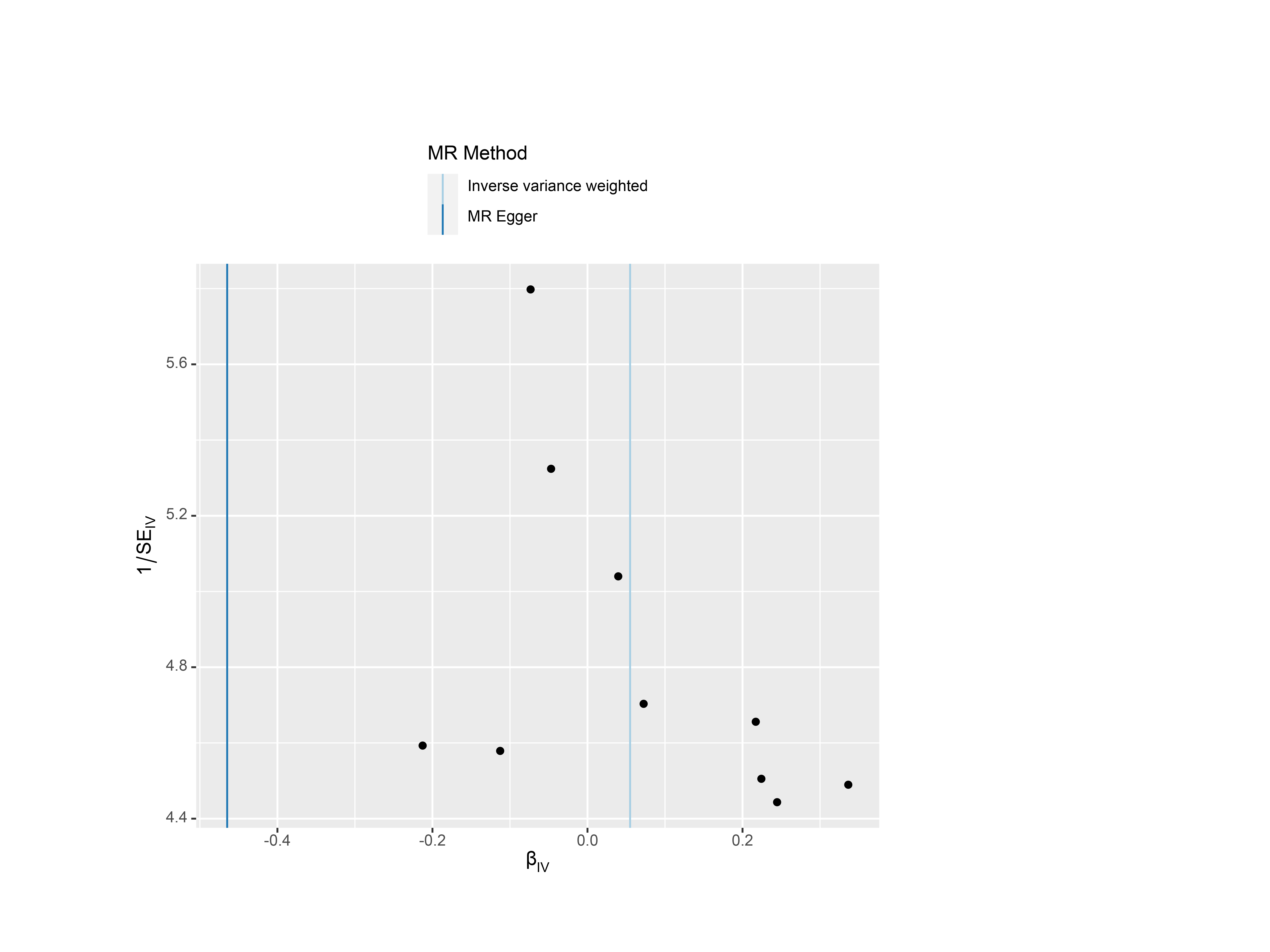

Supplement: S1 Data — (ZIP) [file pone.0309124.s002.zip › Data Sheet/Additional file 2 Funnel plot figure/S8 Low hand grip strength on cognitive function.tif]

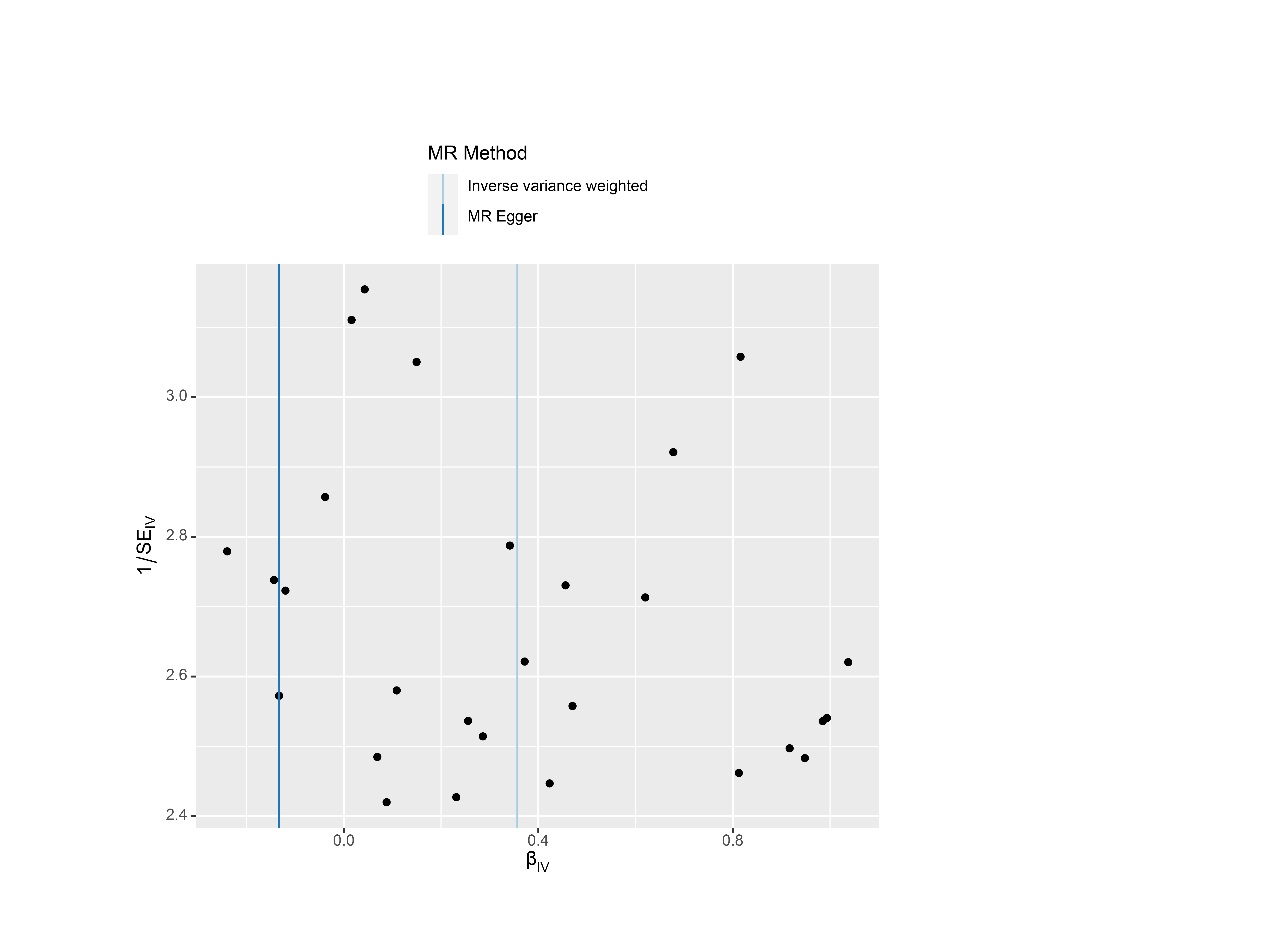

Supplement: S1 Data — (ZIP) [file pone.0309124.s002.zip › Data Sheet/Additional file 2 Funnel plot figure/S9 Walking pace on cognitive performance.tif]

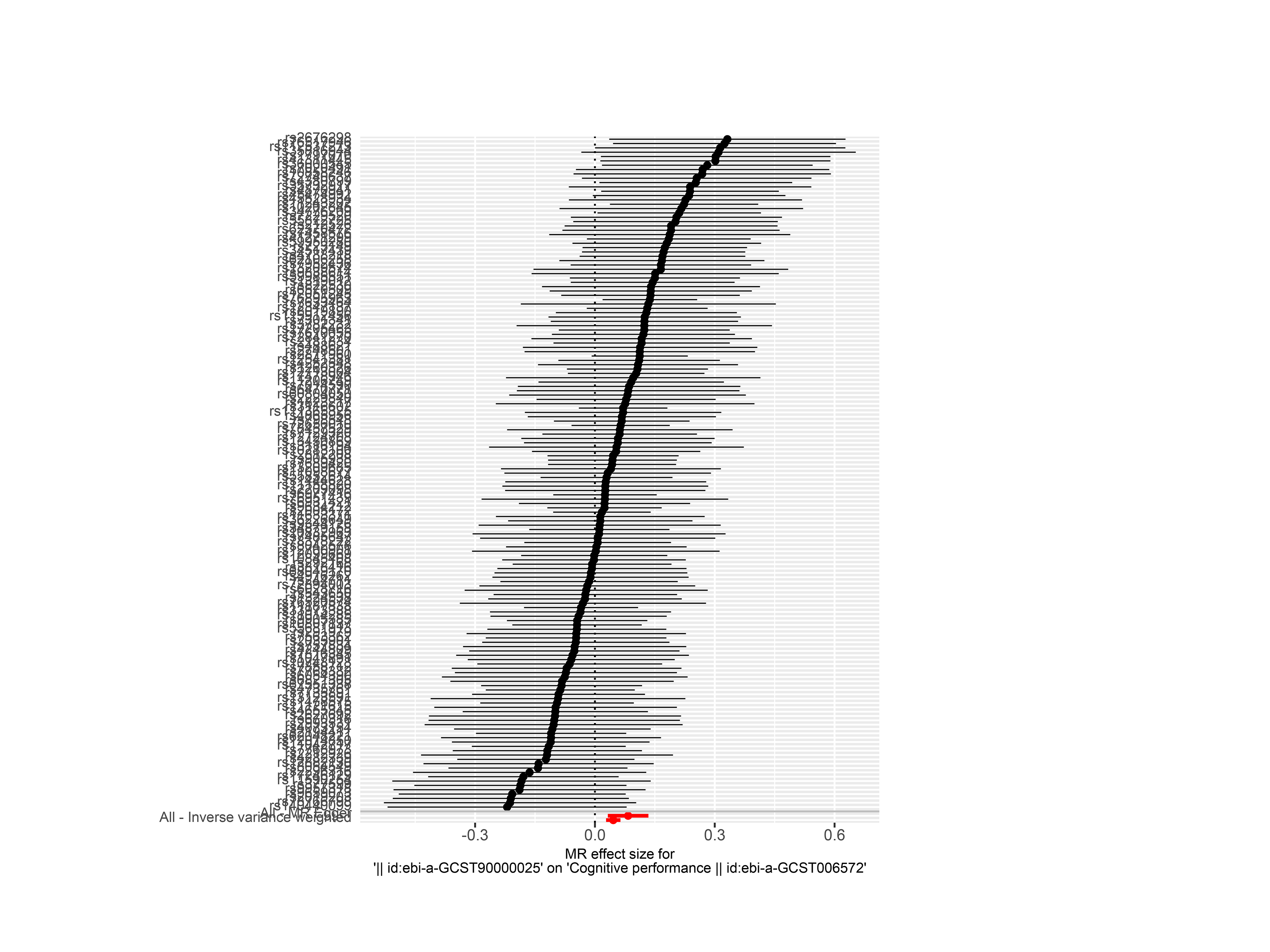

Supplement: S1 Data — (ZIP) [file pone.0309124.s002.zip › Data Sheet/Additional file 3 Forest plot figure/T1 ALM on cognitive performance.tif]

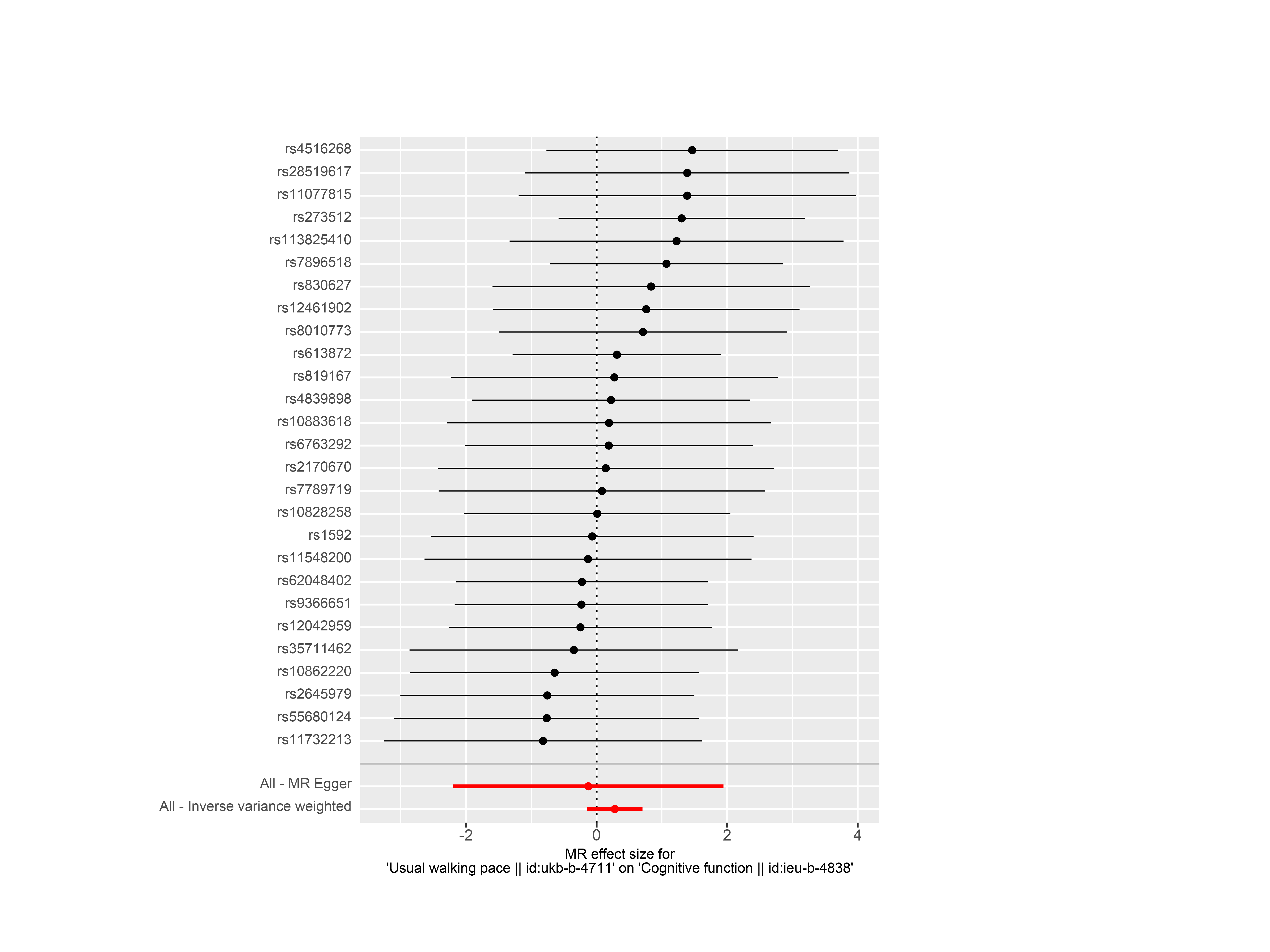

Supplement: S1 Data — (ZIP) [file pone.0309124.s002.zip › Data Sheet/Additional file 3 Forest plot figure/T10 Walking pace on cognitive function.tif]

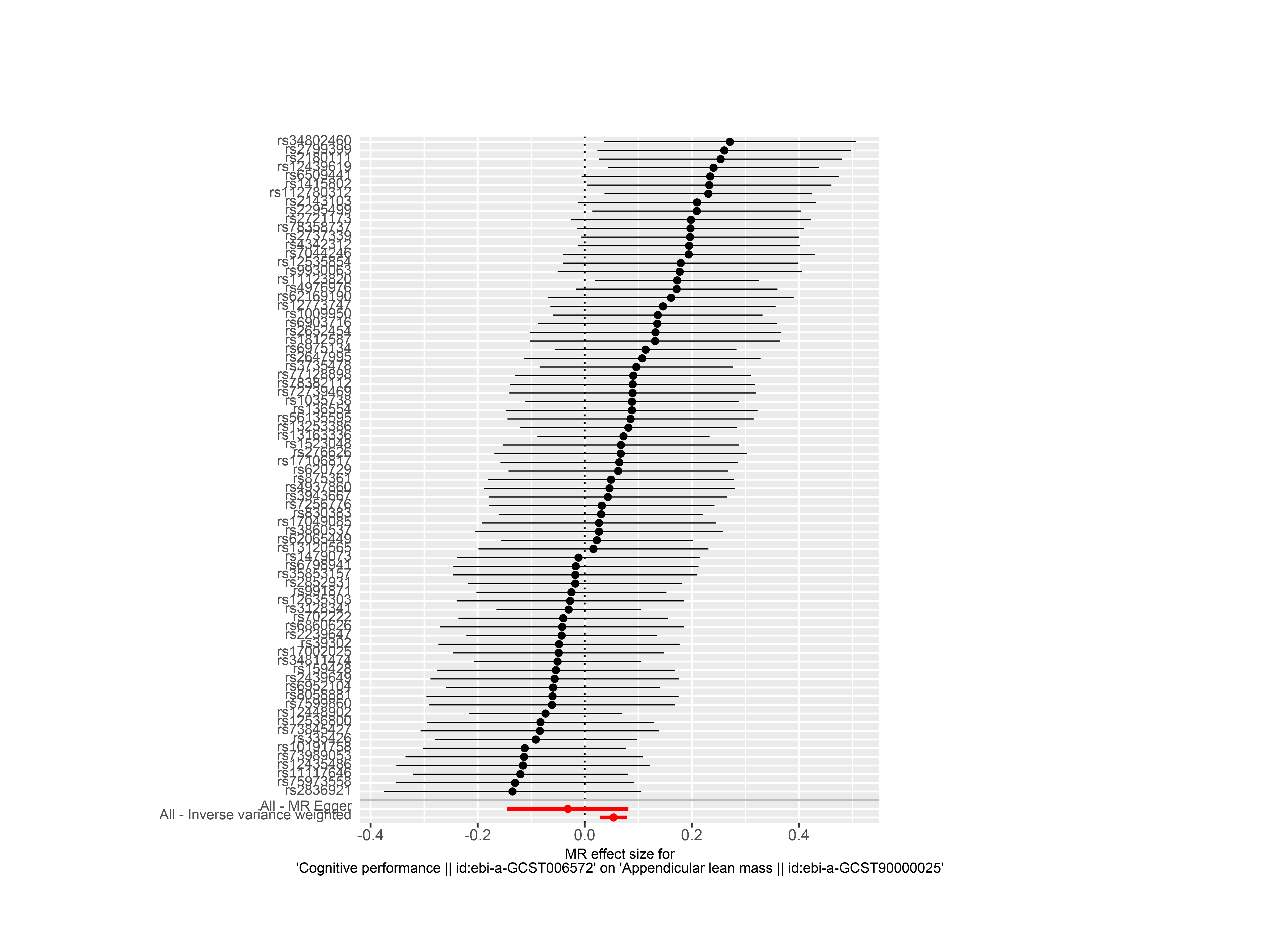

Supplement: S1 Data — (ZIP) [file pone.0309124.s002.zip › Data Sheet/Additional file 3 Forest plot figure/T11 Cognitive performance on ALM.tif]

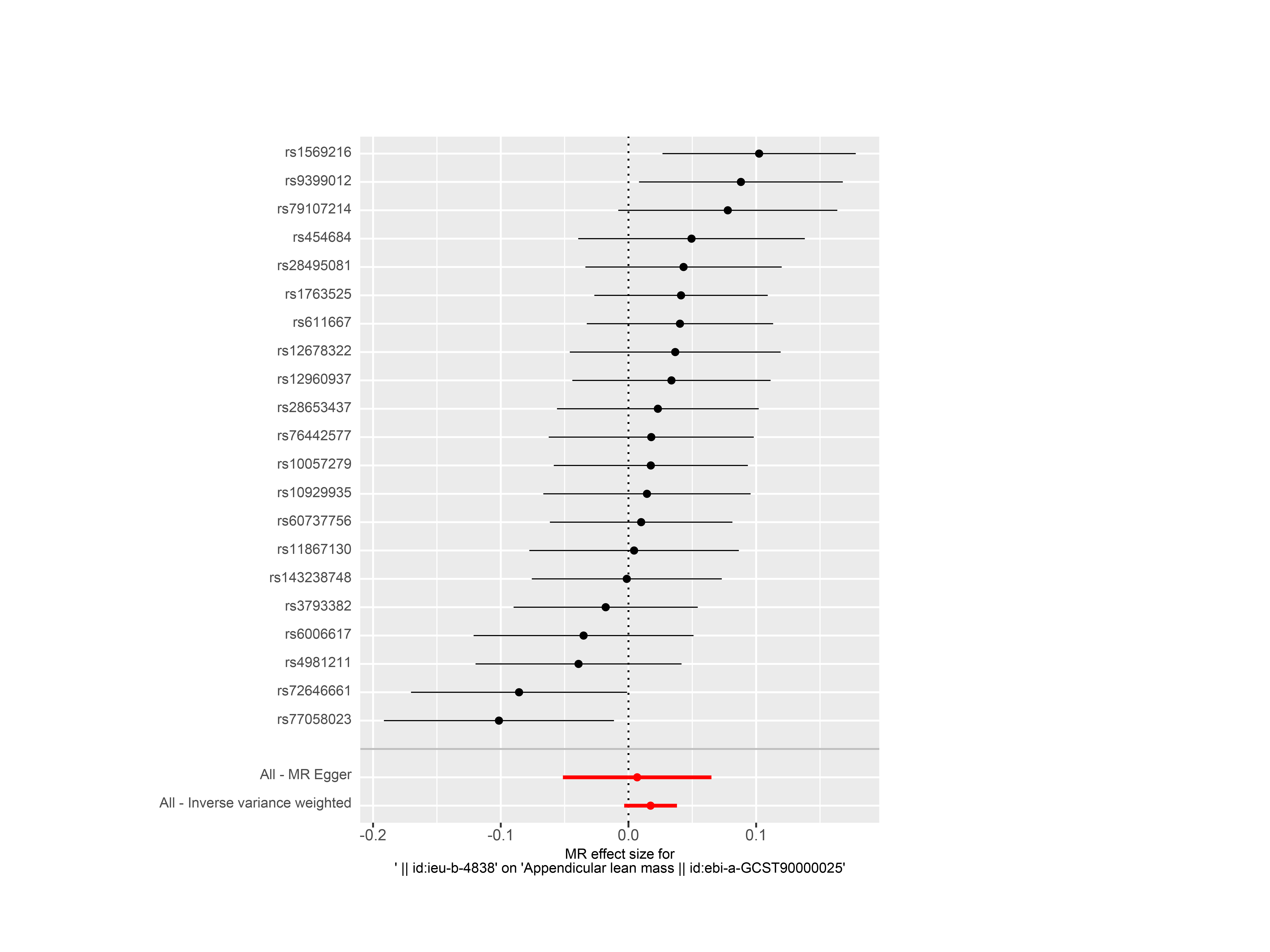

Supplement: S1 Data — (ZIP) [file pone.0309124.s002.zip › Data Sheet/Additional file 3 Forest plot figure/T12 Cognitive function on ALM.tif]

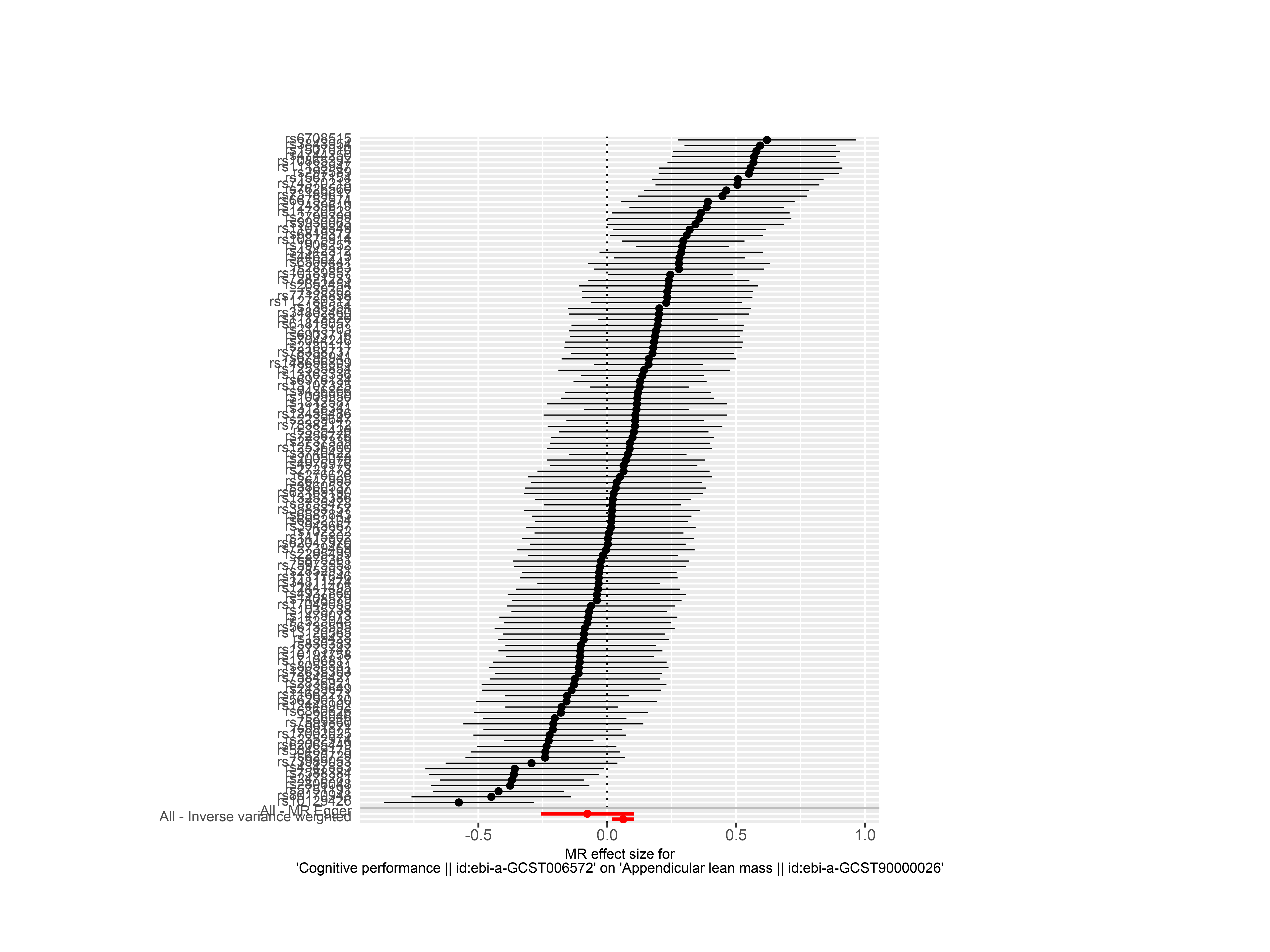

Supplement: S1 Data — (ZIP) [file pone.0309124.s002.zip › Data Sheet/Additional file 3 Forest plot figure/T13 Cognitive performance on ALM-M.tif]

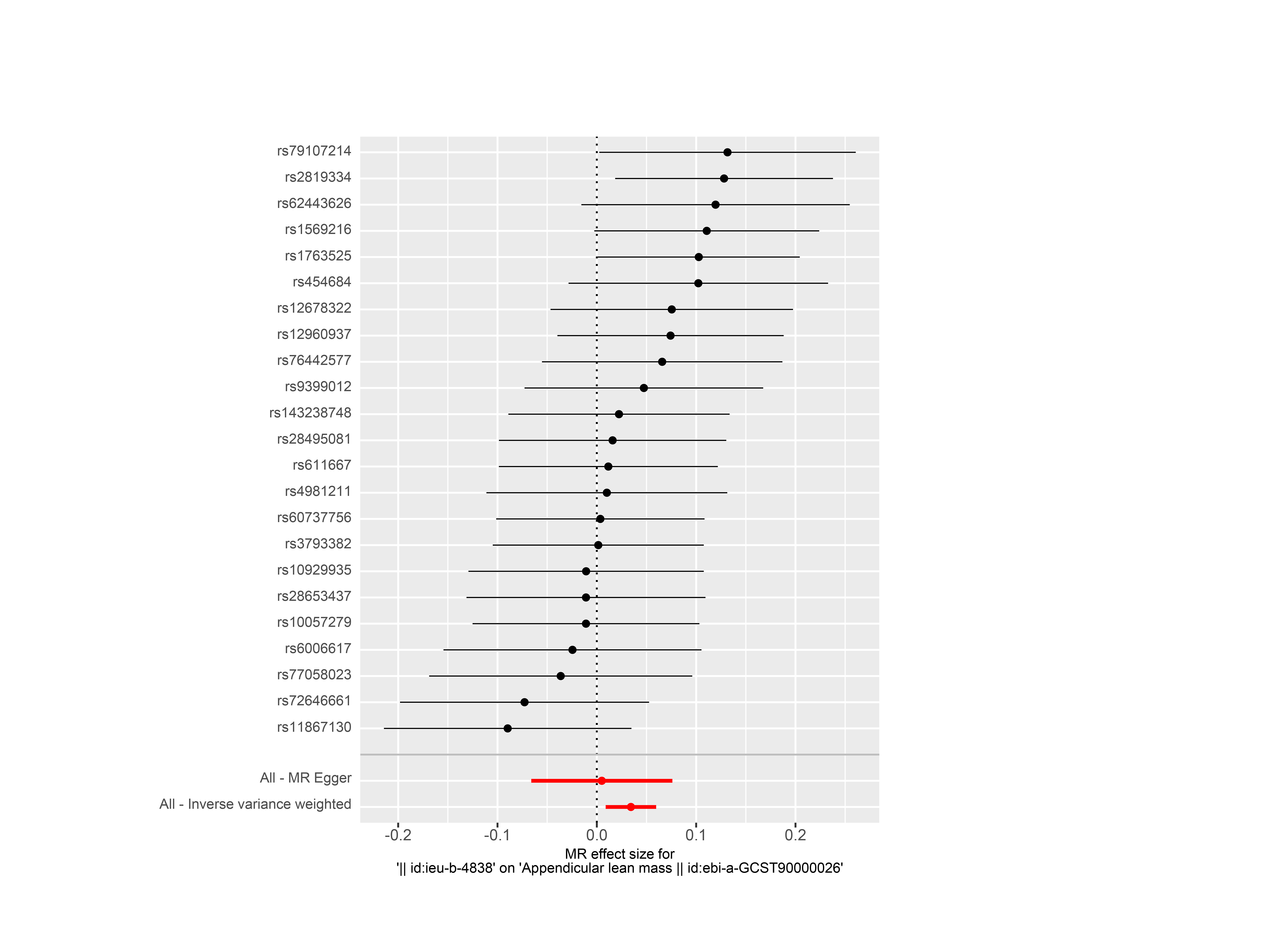

Supplement: S1 Data — (ZIP) [file pone.0309124.s002.zip › Data Sheet/Additional file 3 Forest plot figure/T14 Cognitive function on ALM-M.tif]

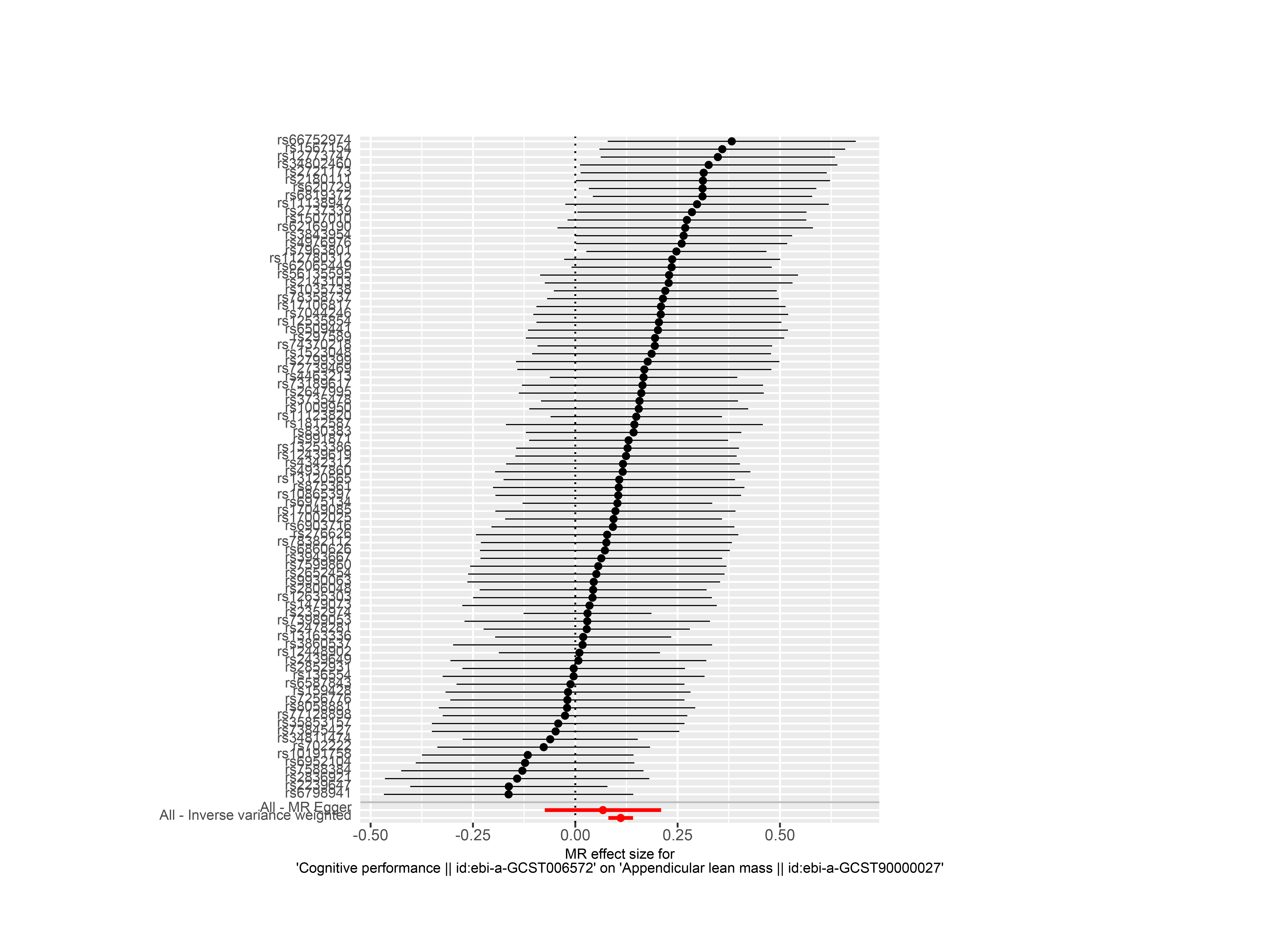

Supplement: S1 Data — (ZIP) [file pone.0309124.s002.zip › Data Sheet/Additional file 3 Forest plot figure/T15 Cognitive performance on ALM-F.tif]

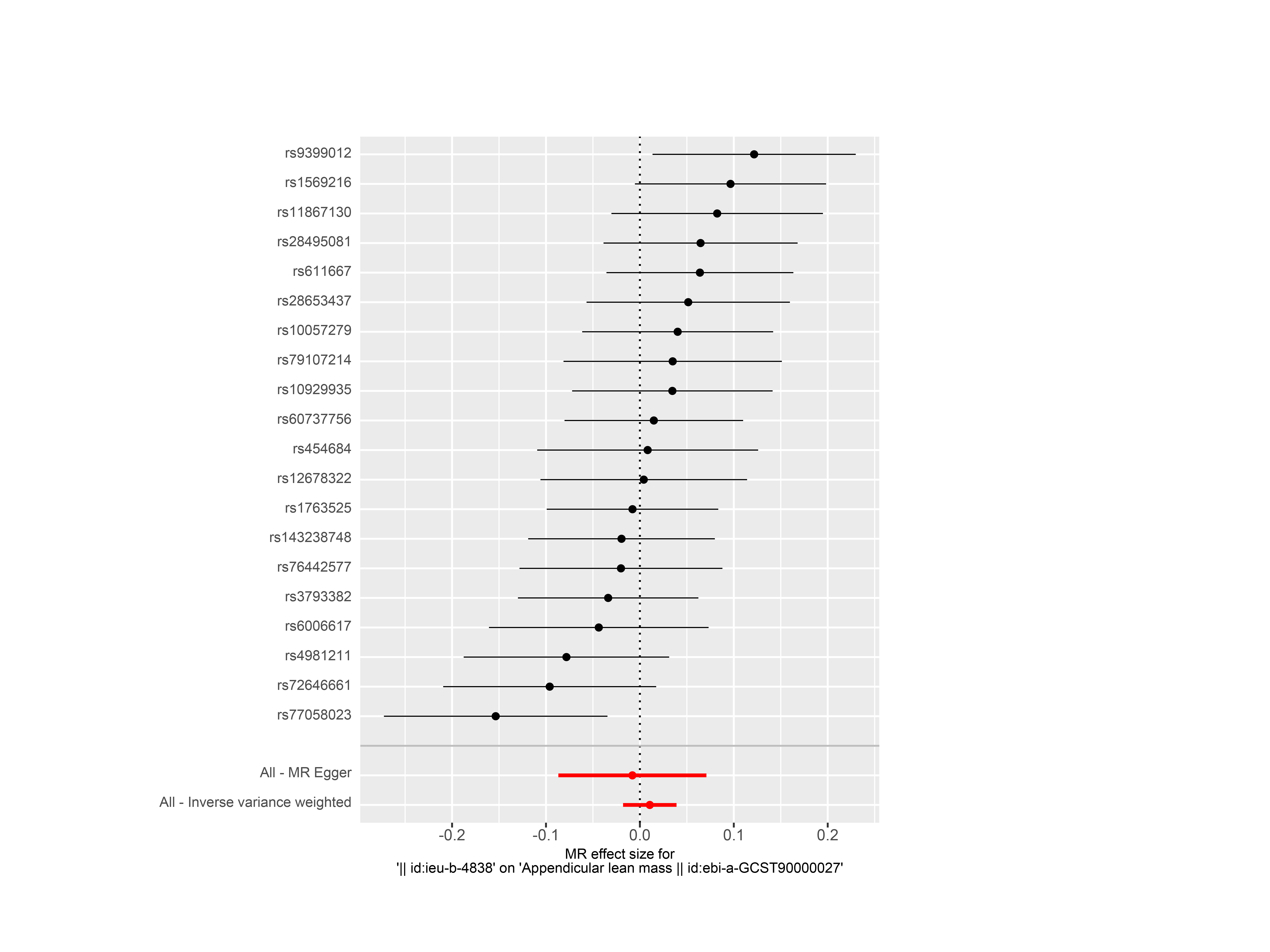

Supplement: S1 Data — (ZIP) [file pone.0309124.s002.zip › Data Sheet/Additional file 3 Forest plot figure/T16 Cognitive function on ALM-F.tif]

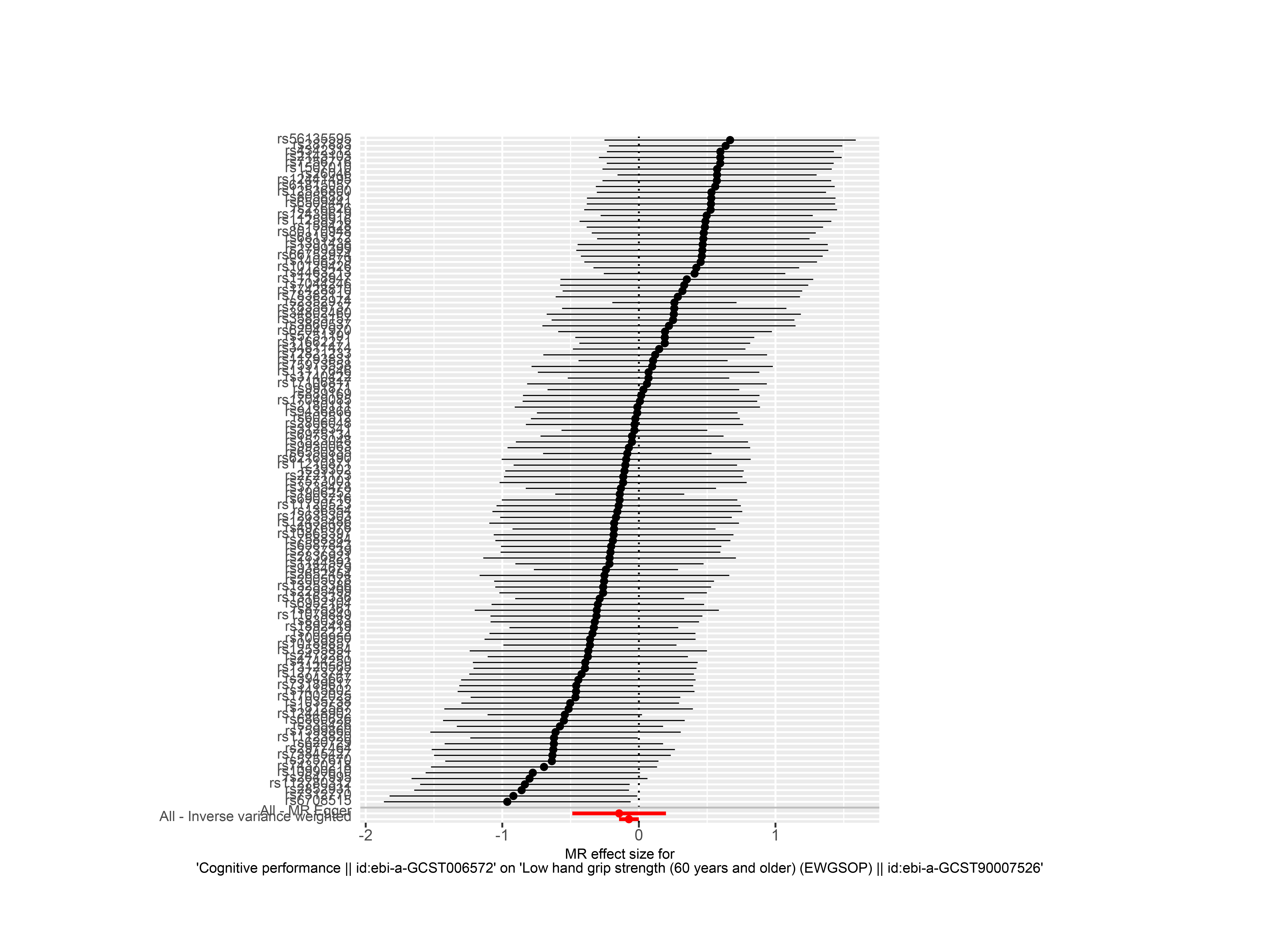

Supplement: S1 Data — (ZIP) [file pone.0309124.s002.zip › Data Sheet/Additional file 3 Forest plot figure/T17 Cognitive performance on low hand grip strength.tif]

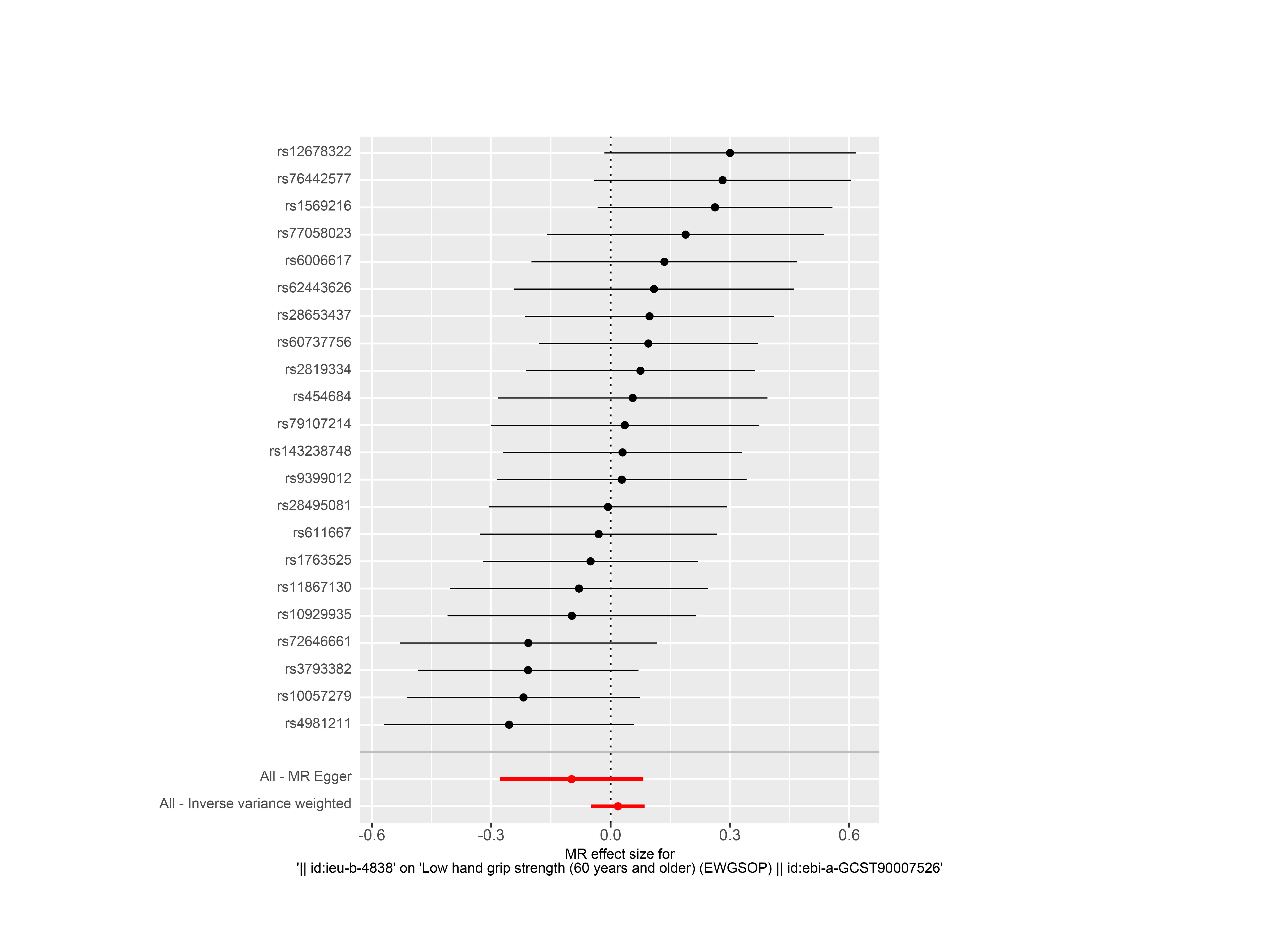

Supplement: S1 Data — (ZIP) [file pone.0309124.s002.zip › Data Sheet/Additional file 3 Forest plot figure/T18 Cognitive function on low hand grip strength.tif]

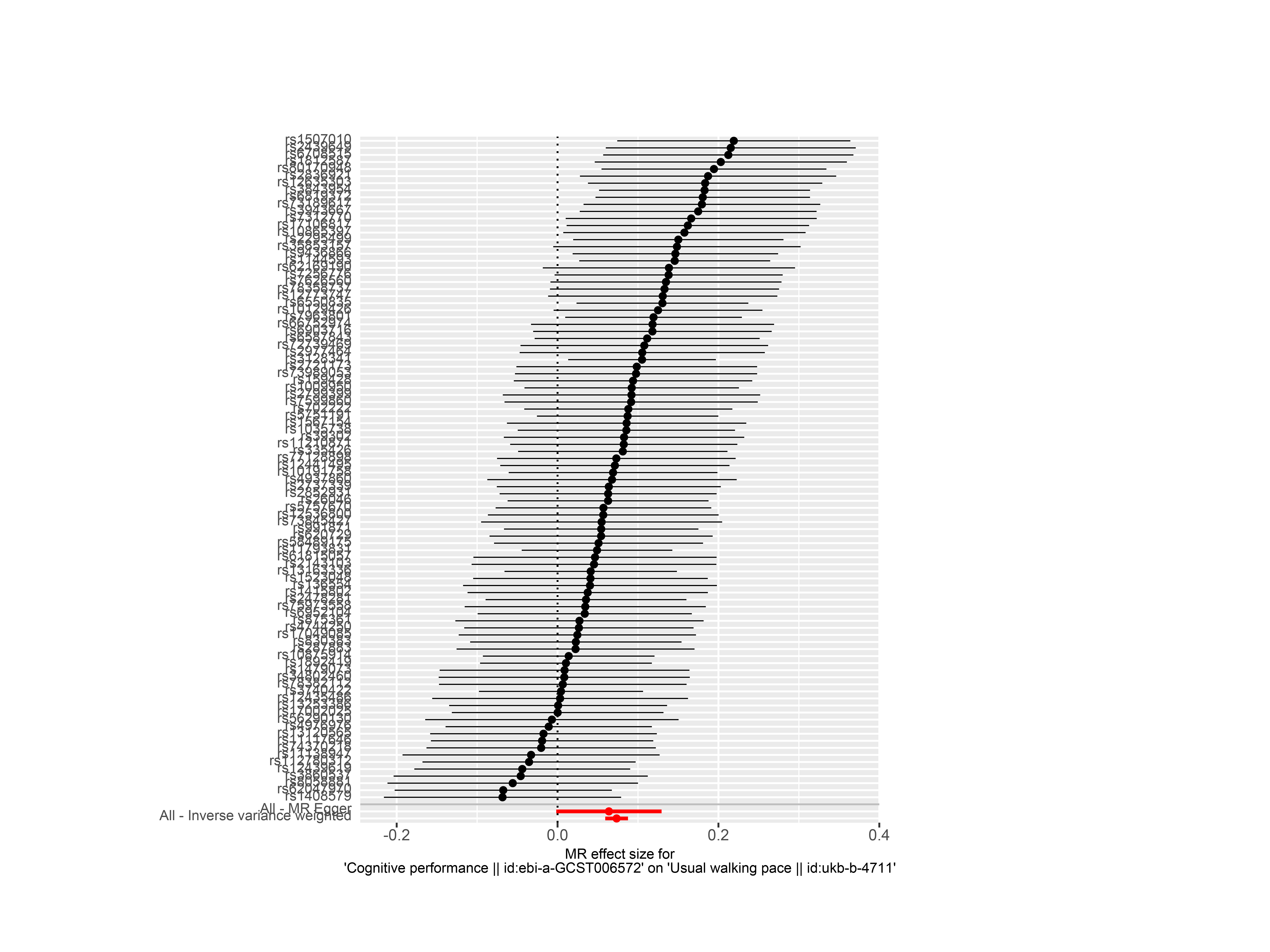

Supplement: S1 Data — (ZIP) [file pone.0309124.s002.zip › Data Sheet/Additional file 3 Forest plot figure/T19 Cognitive performance on walking pace.tif]

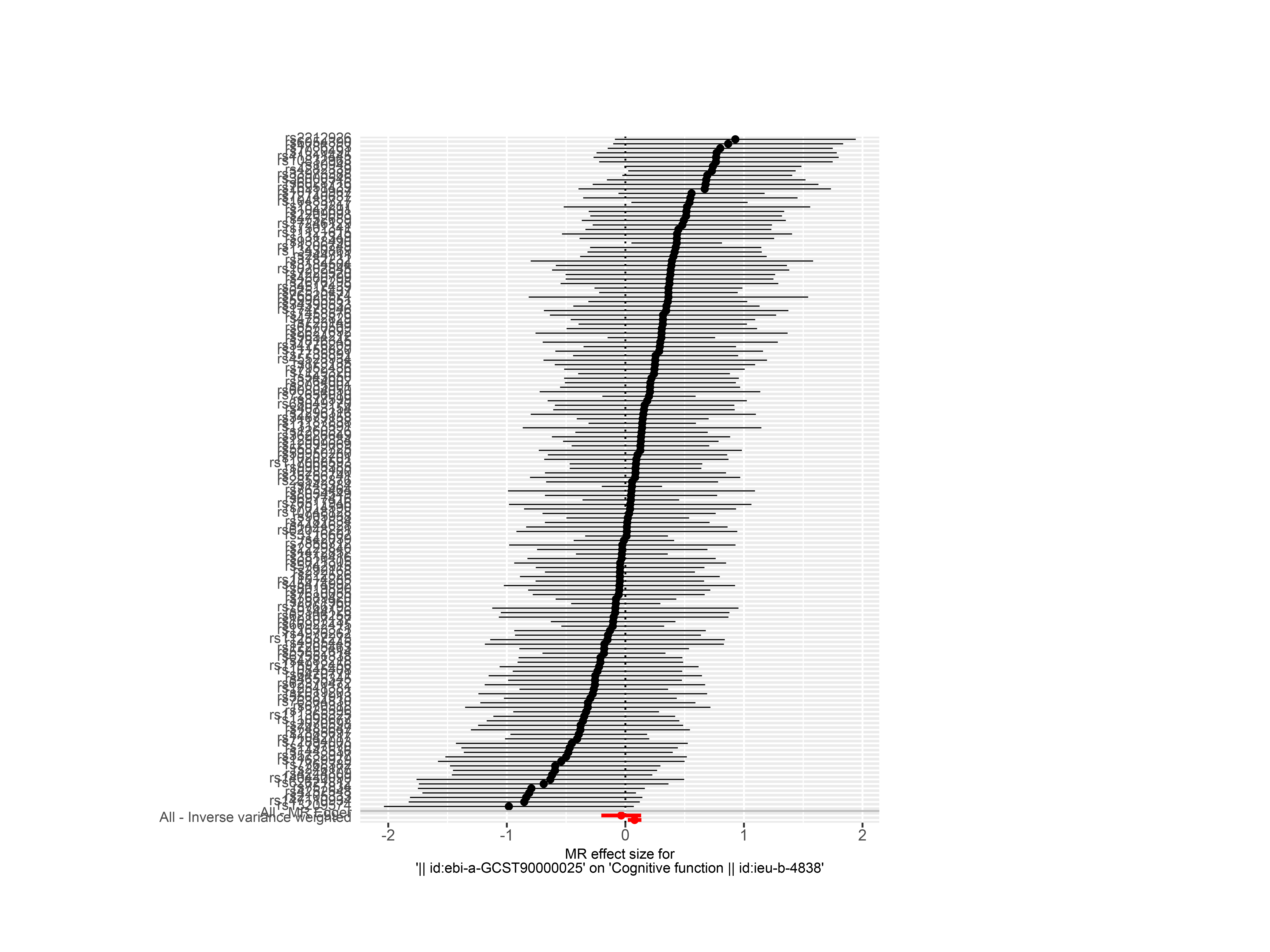

Supplement: S1 Data — (ZIP) [file pone.0309124.s002.zip › Data Sheet/Additional file 3 Forest plot figure/T2 ALM on cognitive function.tif]

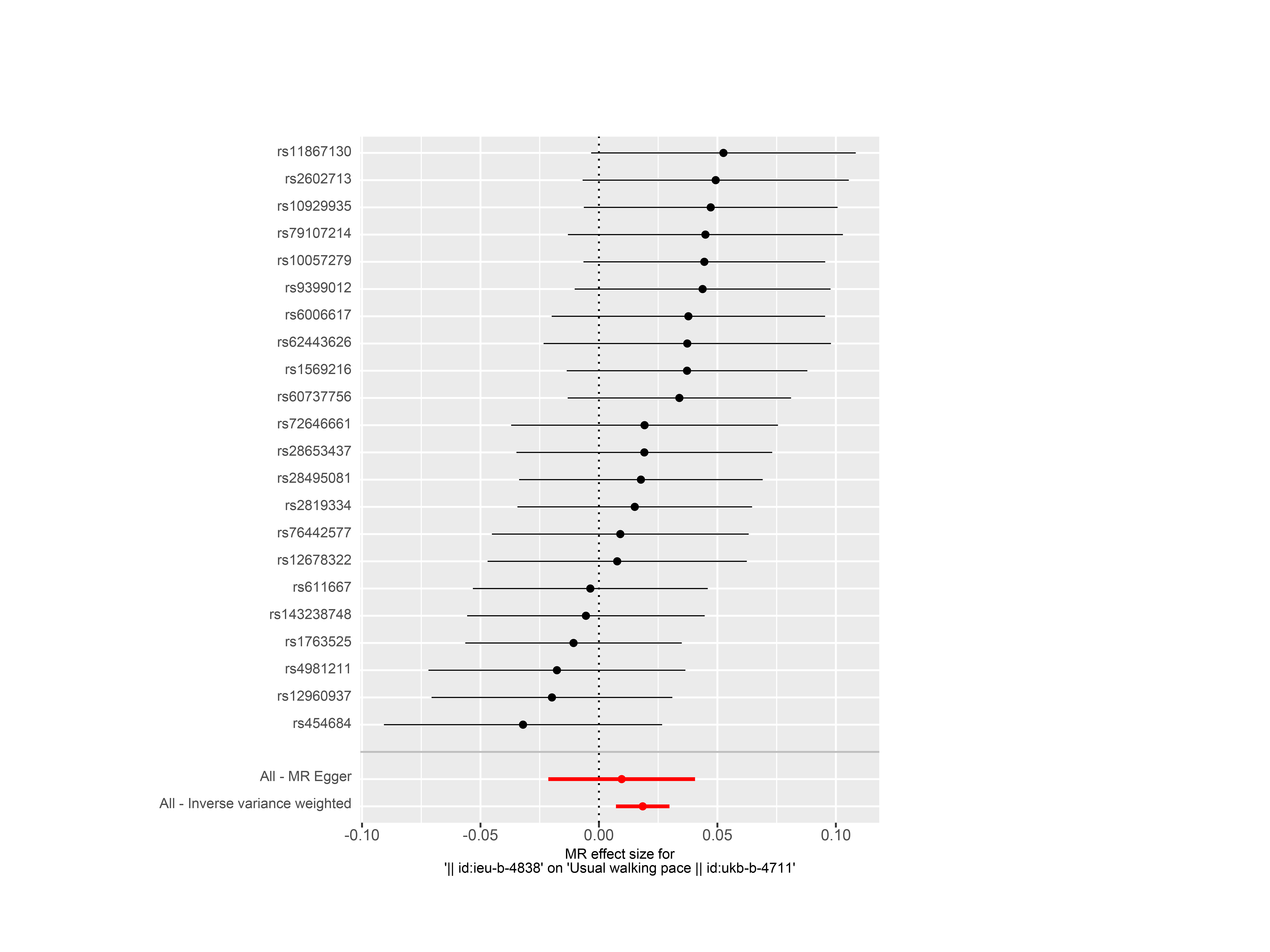

Supplement: S1 Data — (ZIP) [file pone.0309124.s002.zip › Data Sheet/Additional file 3 Forest plot figure/T20 Cognitive function on walking pace.tif]

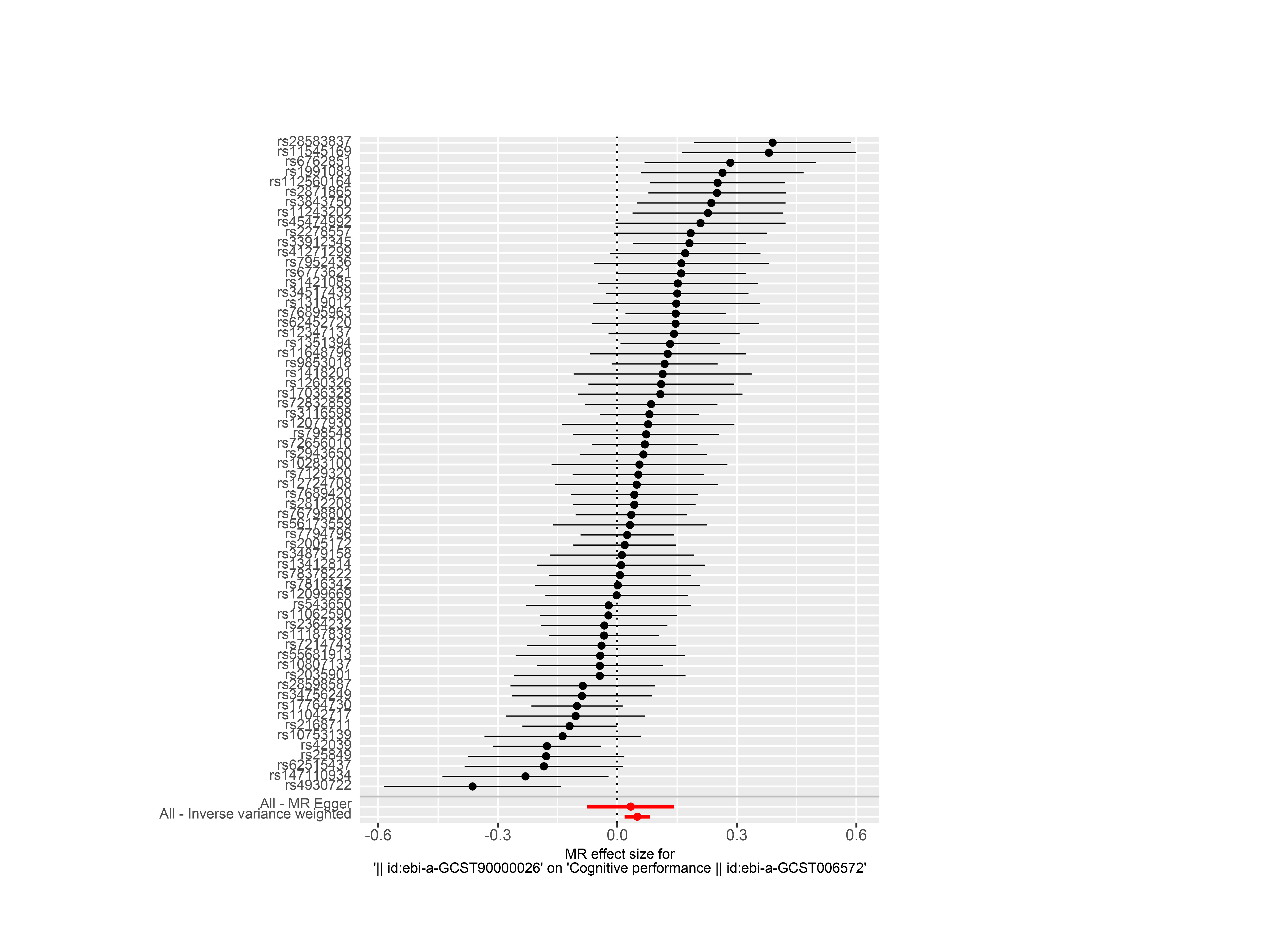

Supplement: S1 Data — (ZIP) [file pone.0309124.s002.zip › Data Sheet/Additional file 3 Forest plot figure/T3 ALM-M on cognitive performnce.tif]

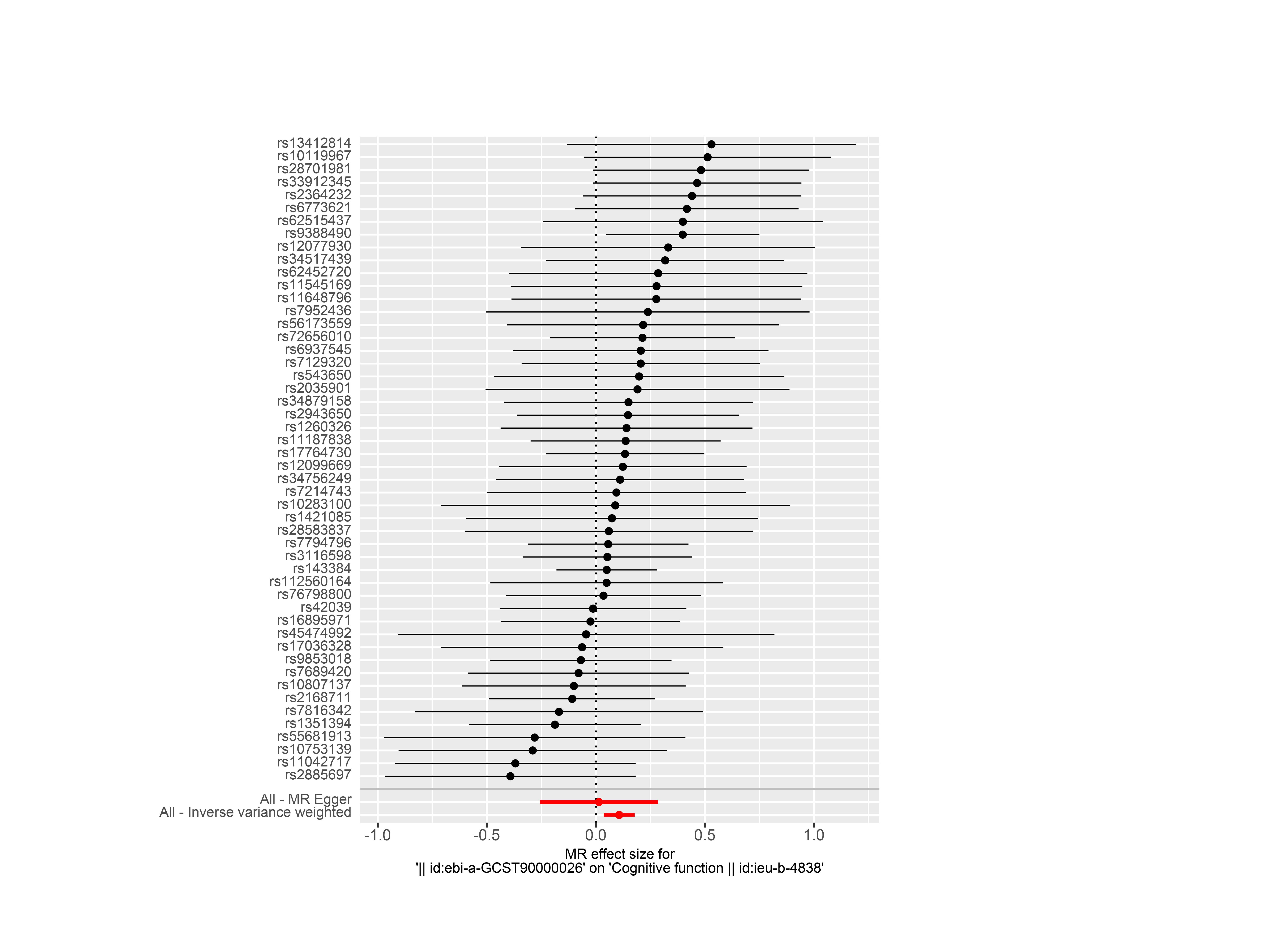

Supplement: S1 Data — (ZIP) [file pone.0309124.s002.zip › Data Sheet/Additional file 3 Forest plot figure/T4 ALM-M on cognitive function.tif]

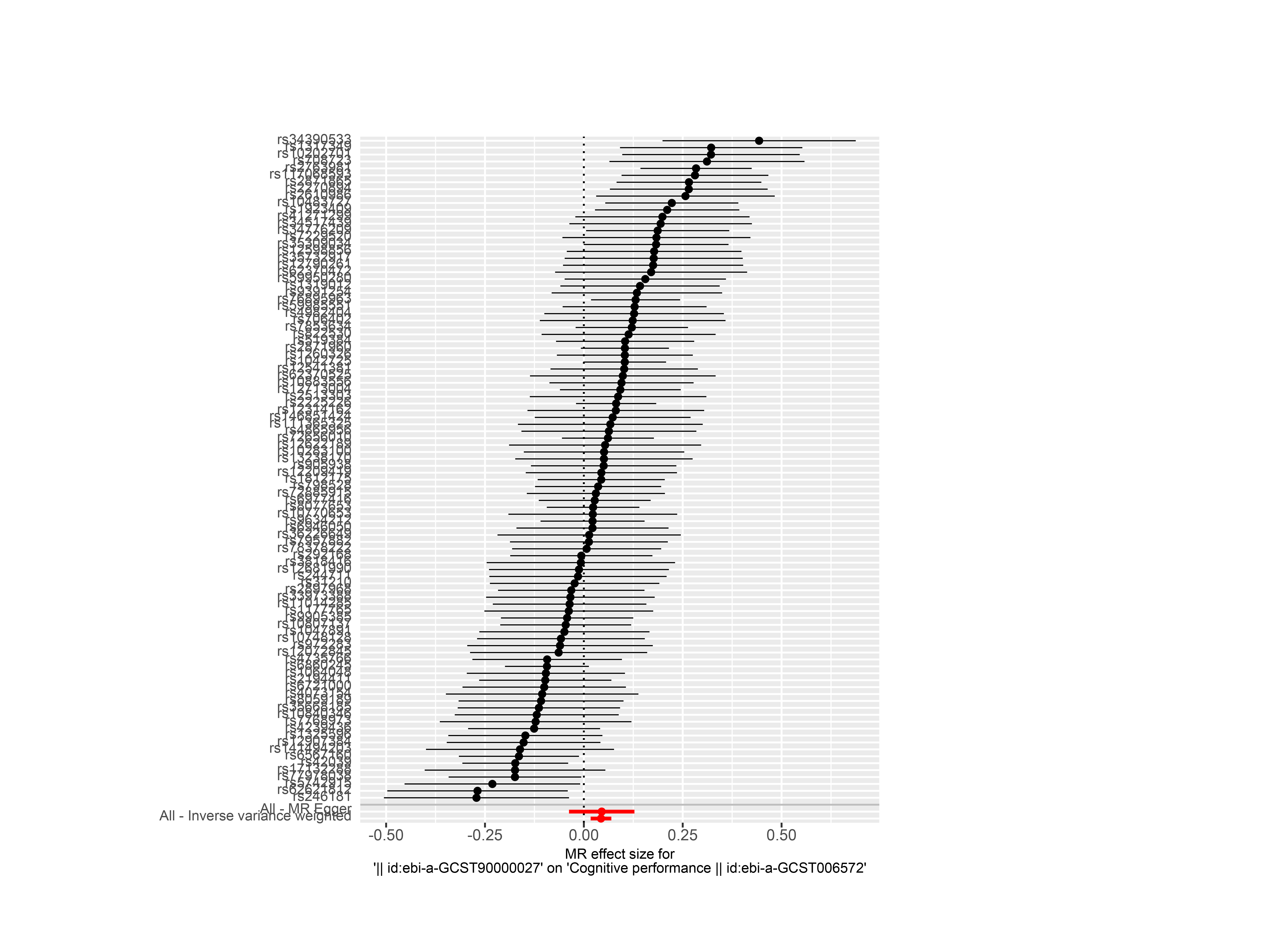

Supplement: S1 Data — (ZIP) [file pone.0309124.s002.zip › Data Sheet/Additional file 3 Forest plot figure/T5 ALM-F on cognitive performance.tif]

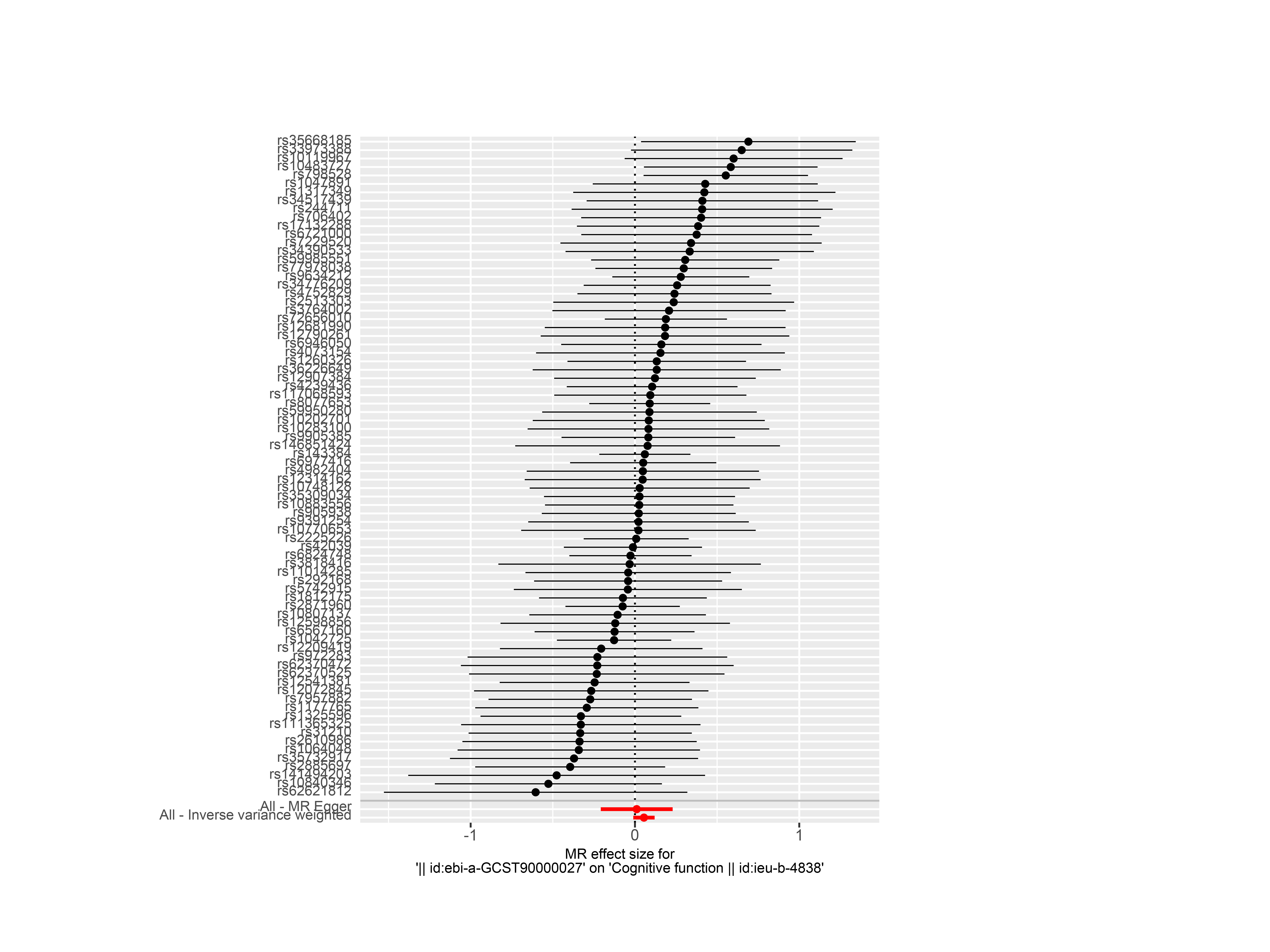

Supplement: S1 Data — (ZIP) [file pone.0309124.s002.zip › Data Sheet/Additional file 3 Forest plot figure/T6 ALM-F on cognitive function.tif]

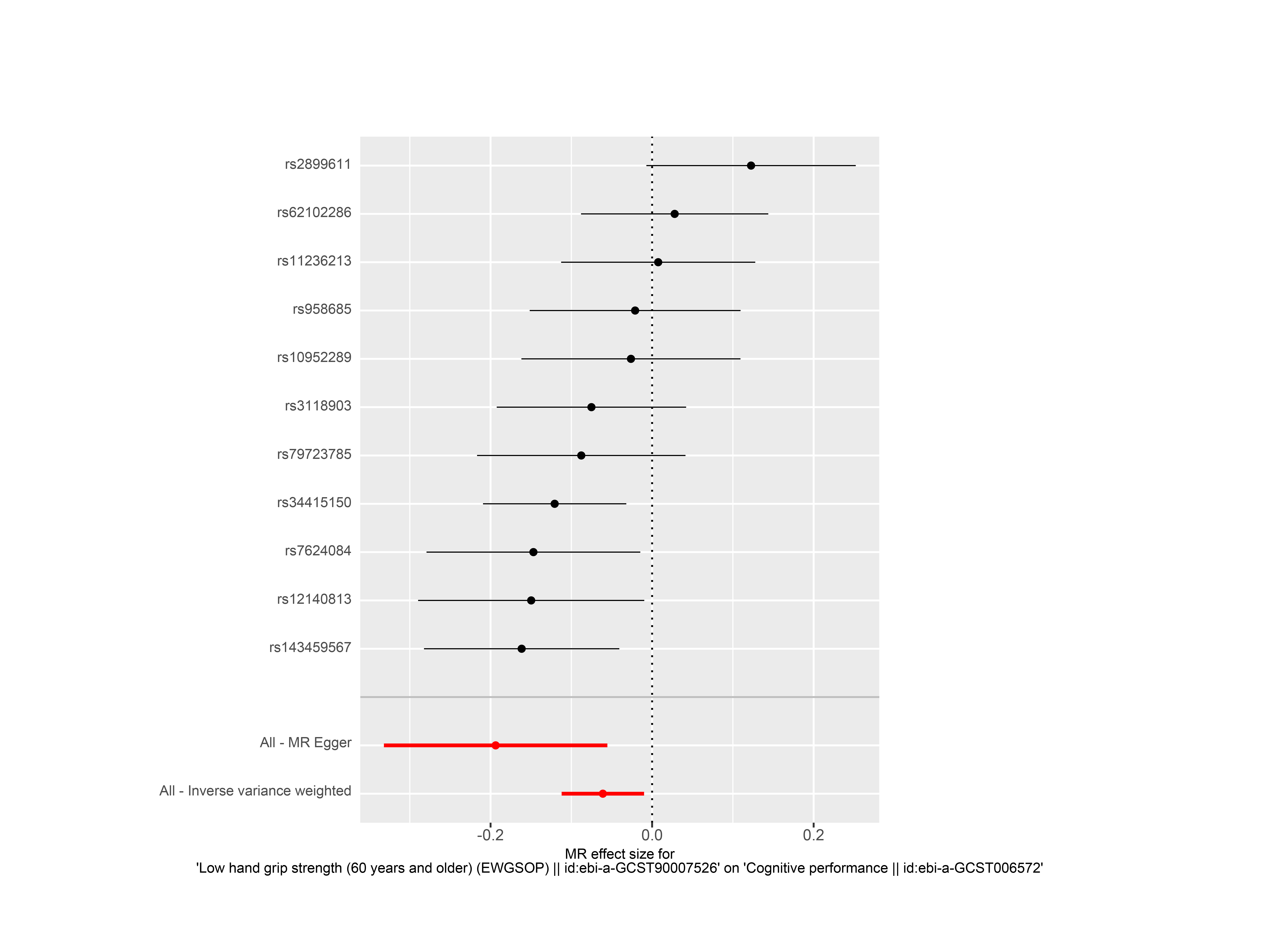

Supplement: S1 Data — (ZIP) [file pone.0309124.s002.zip › Data Sheet/Additional file 3 Forest plot figure/T7 Low hand grip strength on cognitive performance.tif]

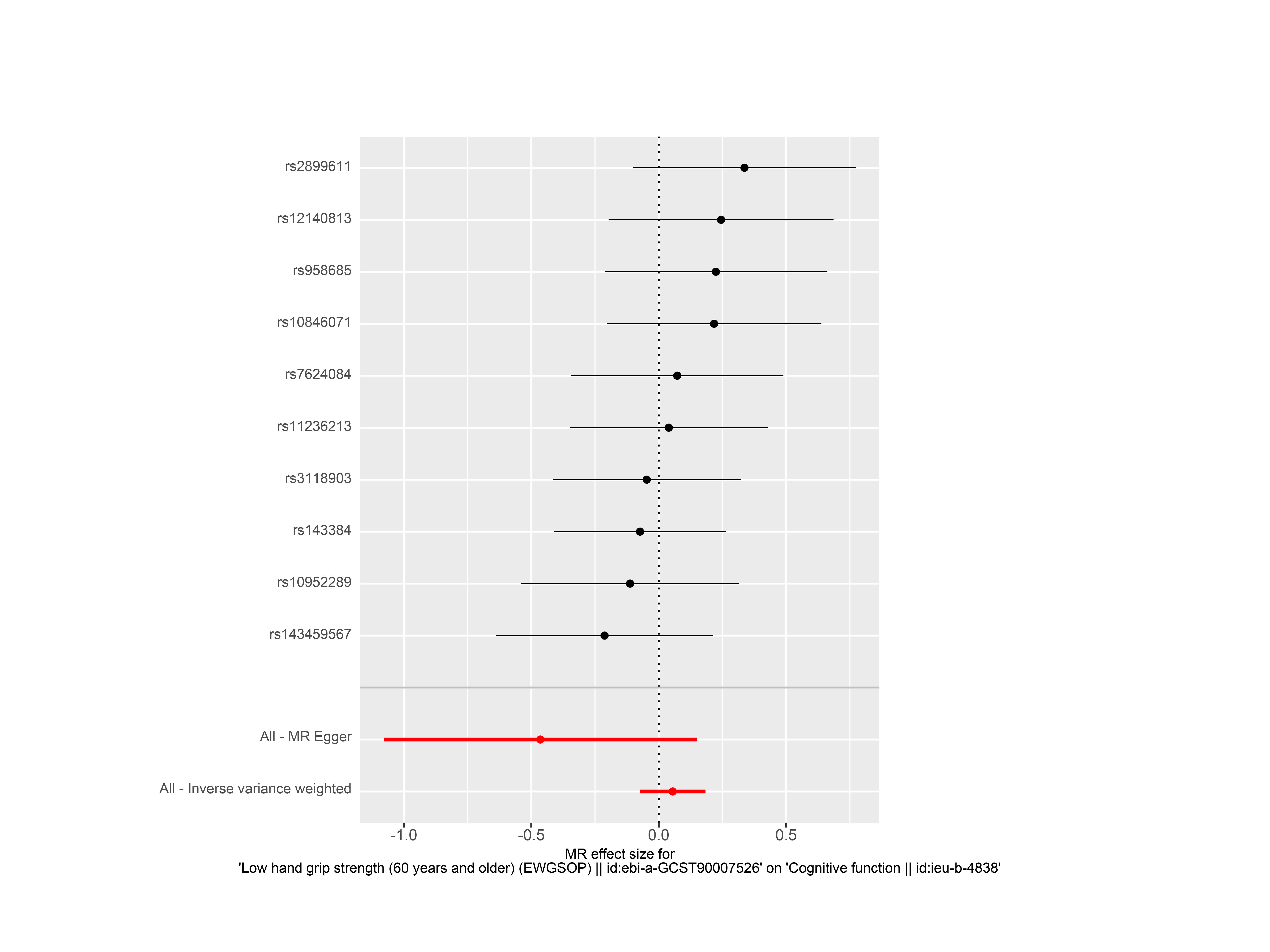

Supplement: S1 Data — (ZIP) [file pone.0309124.s002.zip › Data Sheet/Additional file 3 Forest plot figure/T8 Low hand grip strength on cognitive function.tif]

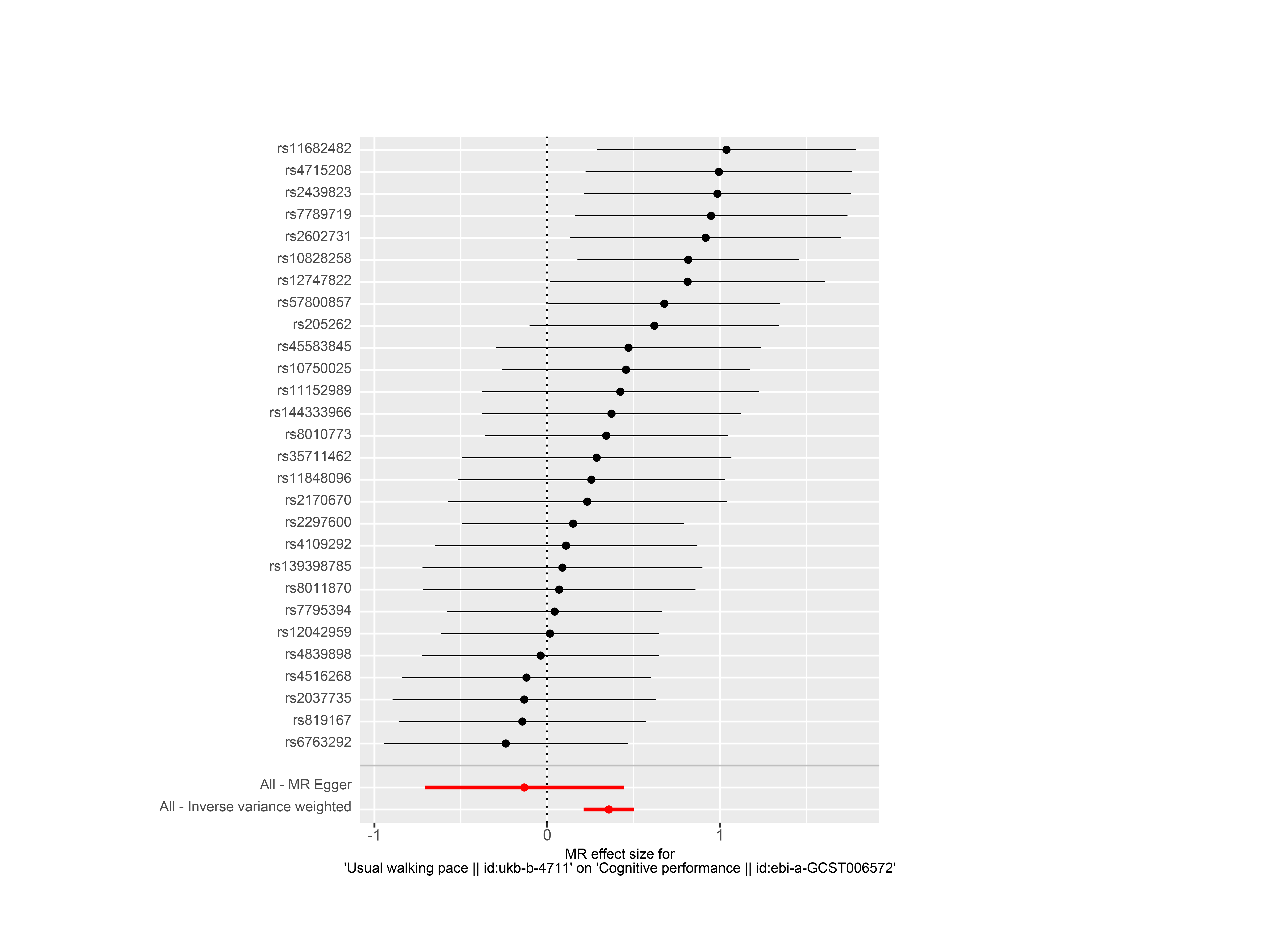

Supplement: S1 Data — (ZIP) [file pone.0309124.s002.zip › Data Sheet/Additional file 3 Forest plot figure/T9 Walking pace on cognitive performance.tif]

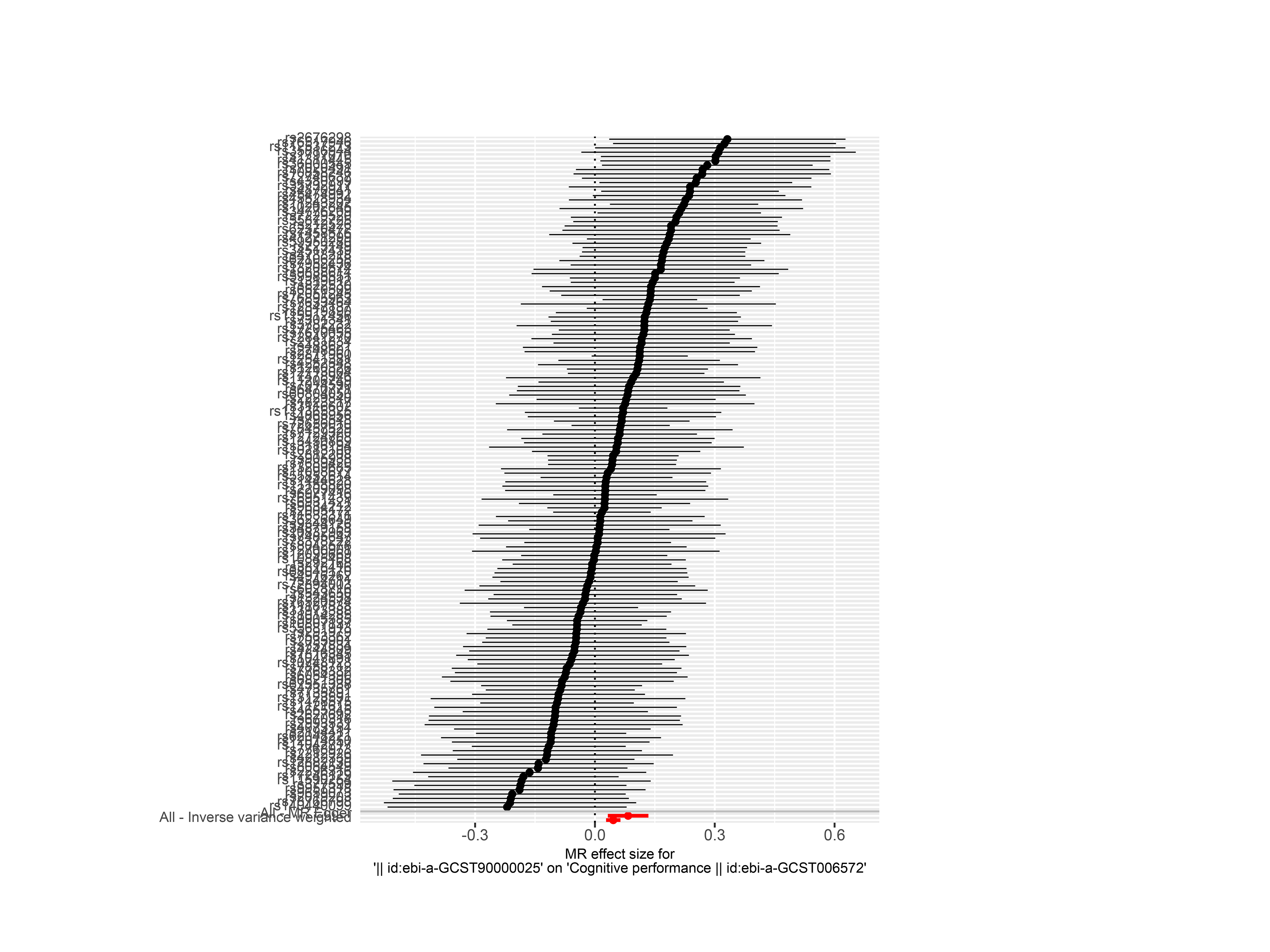

Supplement: S1 Data — (ZIP) [file pone.0309124.s002.zip › Data Sheet/Additional file 4 Leave-one-out sensitivity analysis/H1 ALM on cognitive performance.tif]

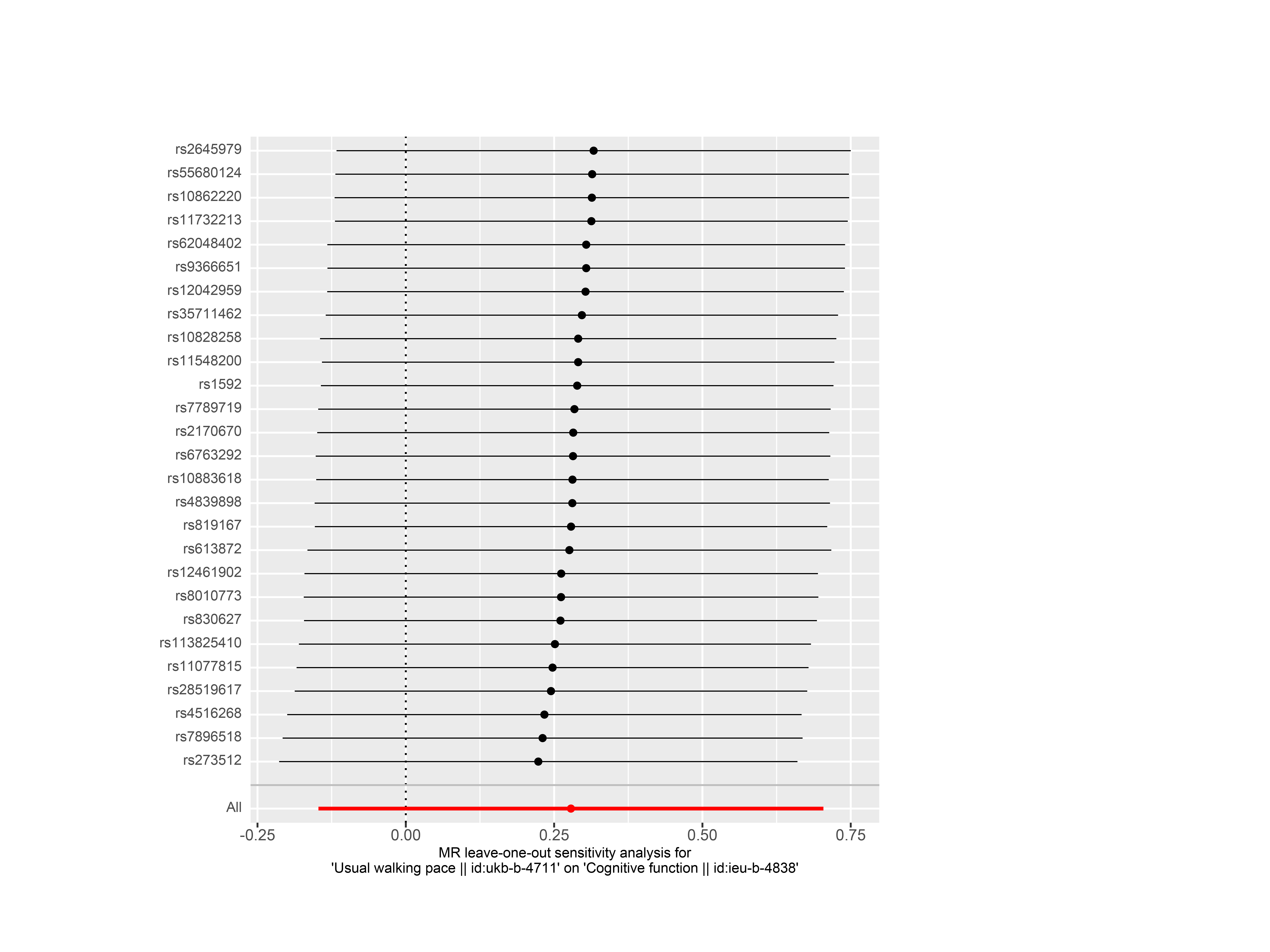

Supplement: S1 Data — (ZIP) [file pone.0309124.s002.zip › Data Sheet/Additional file 4 Leave-one-out sensitivity analysis/H10 Walking pace on cognitive function.tif]

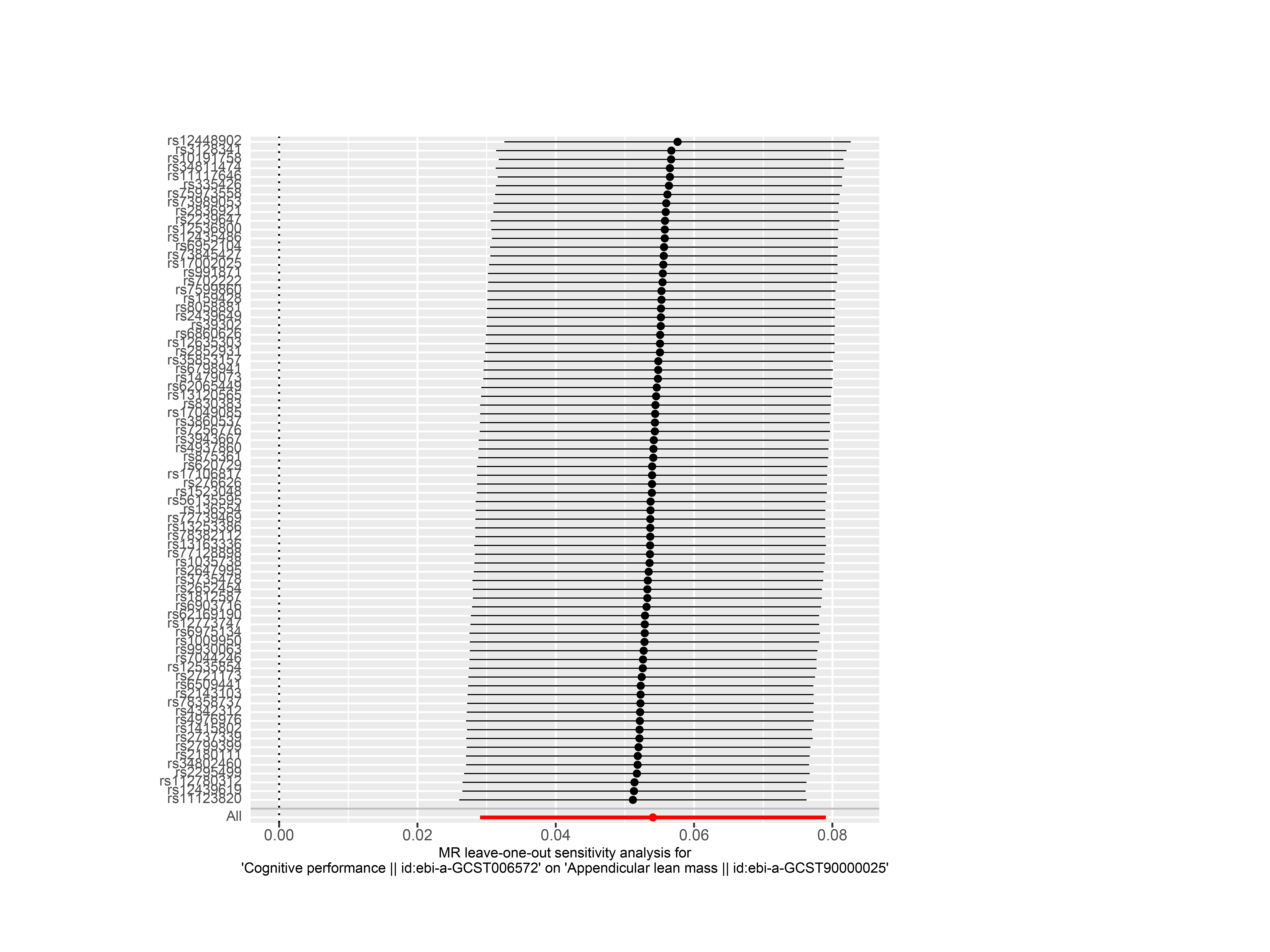

Supplement: S1 Data — (ZIP) [file pone.0309124.s002.zip › Data Sheet/Additional file 4 Leave-one-out sensitivity analysis/H11 Cognitive performance on ALM.tif]

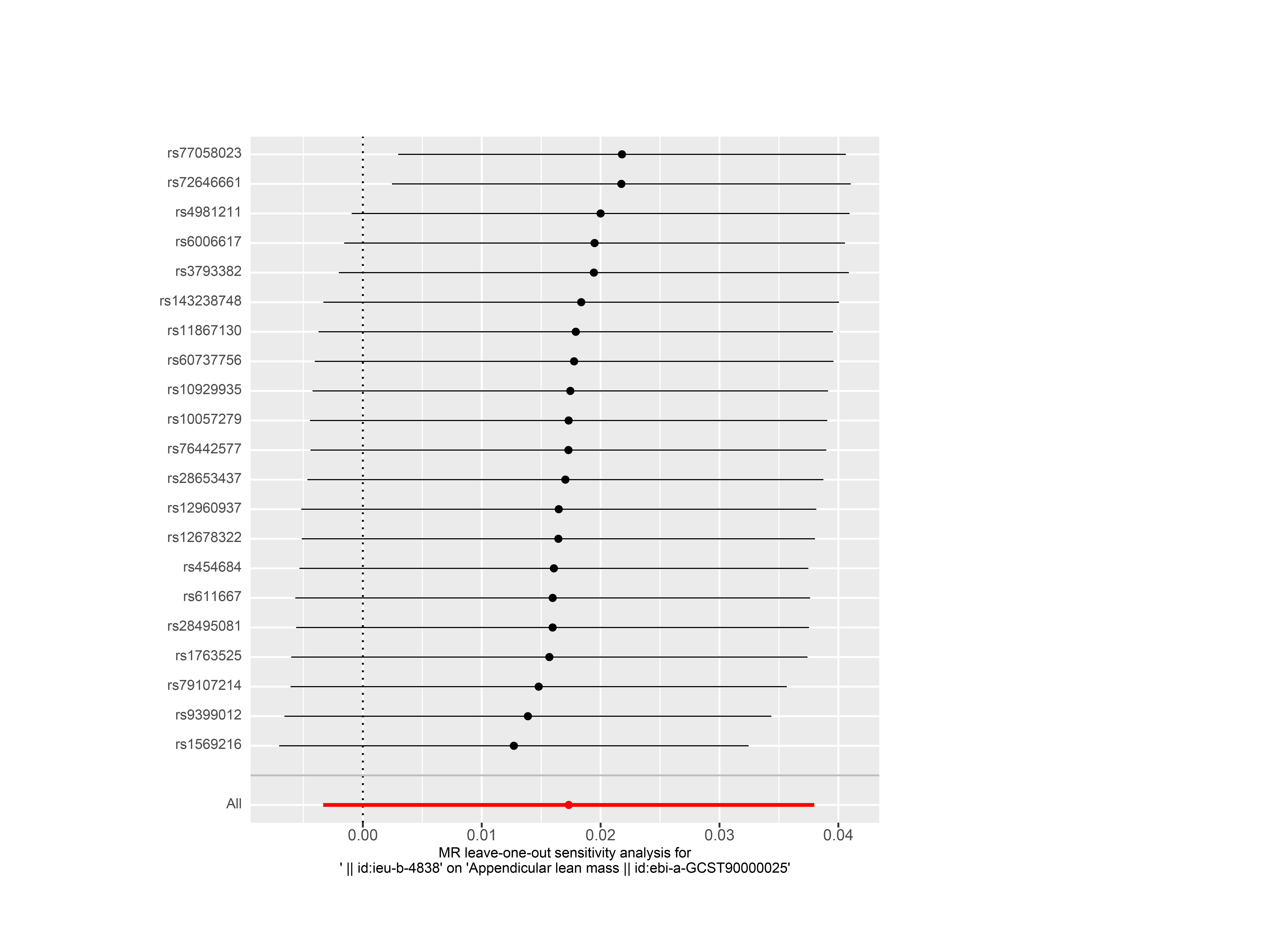

Supplement: S1 Data — (ZIP) [file pone.0309124.s002.zip › Data Sheet/Additional file 4 Leave-one-out sensitivity analysis/H12 Cognitive function on ALM.tif]

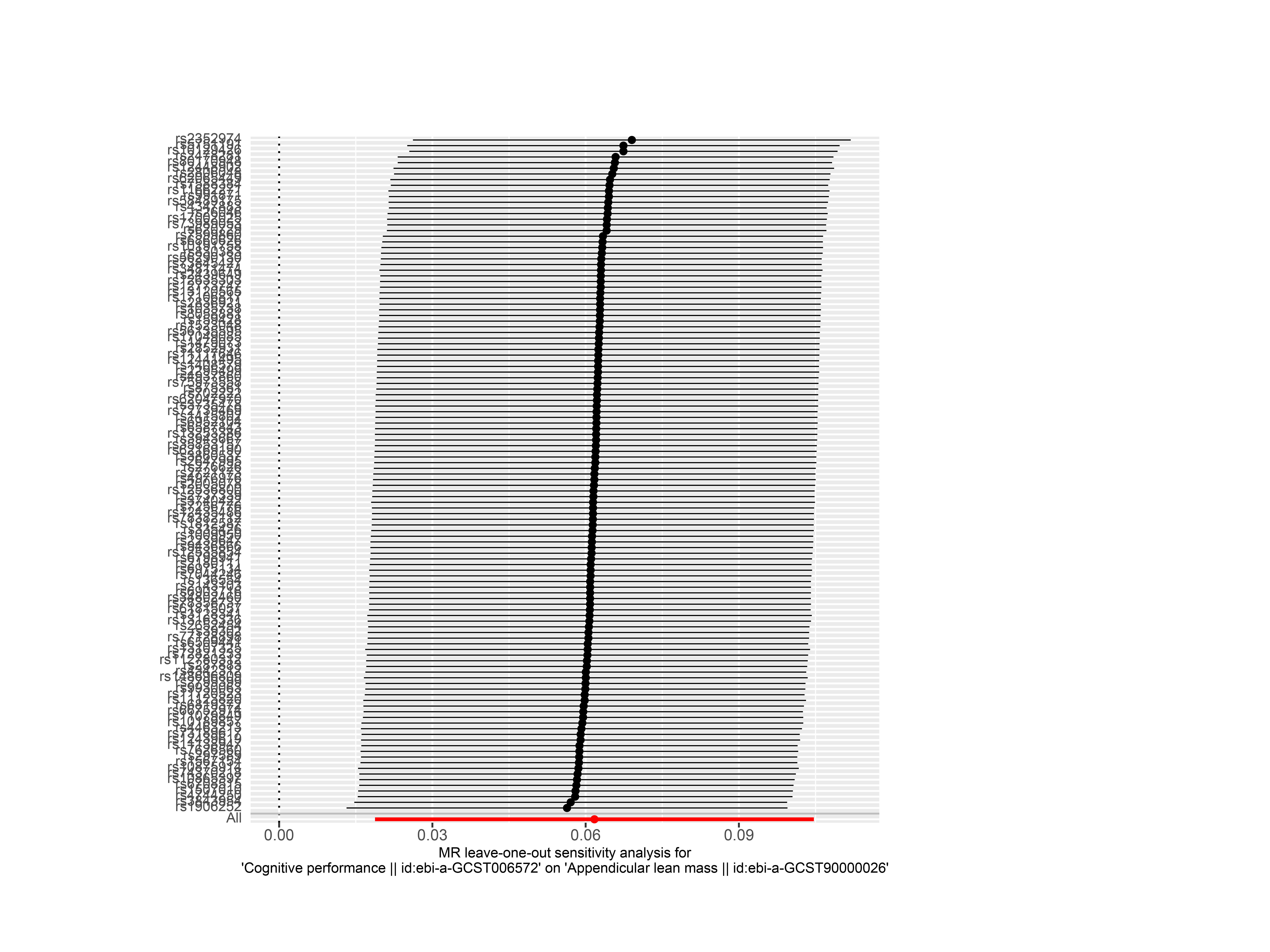

Supplement: S1 Data — (ZIP) [file pone.0309124.s002.zip › Data Sheet/Additional file 4 Leave-one-out sensitivity analysis/H13 Cognitive performance on ALM-M.tif]

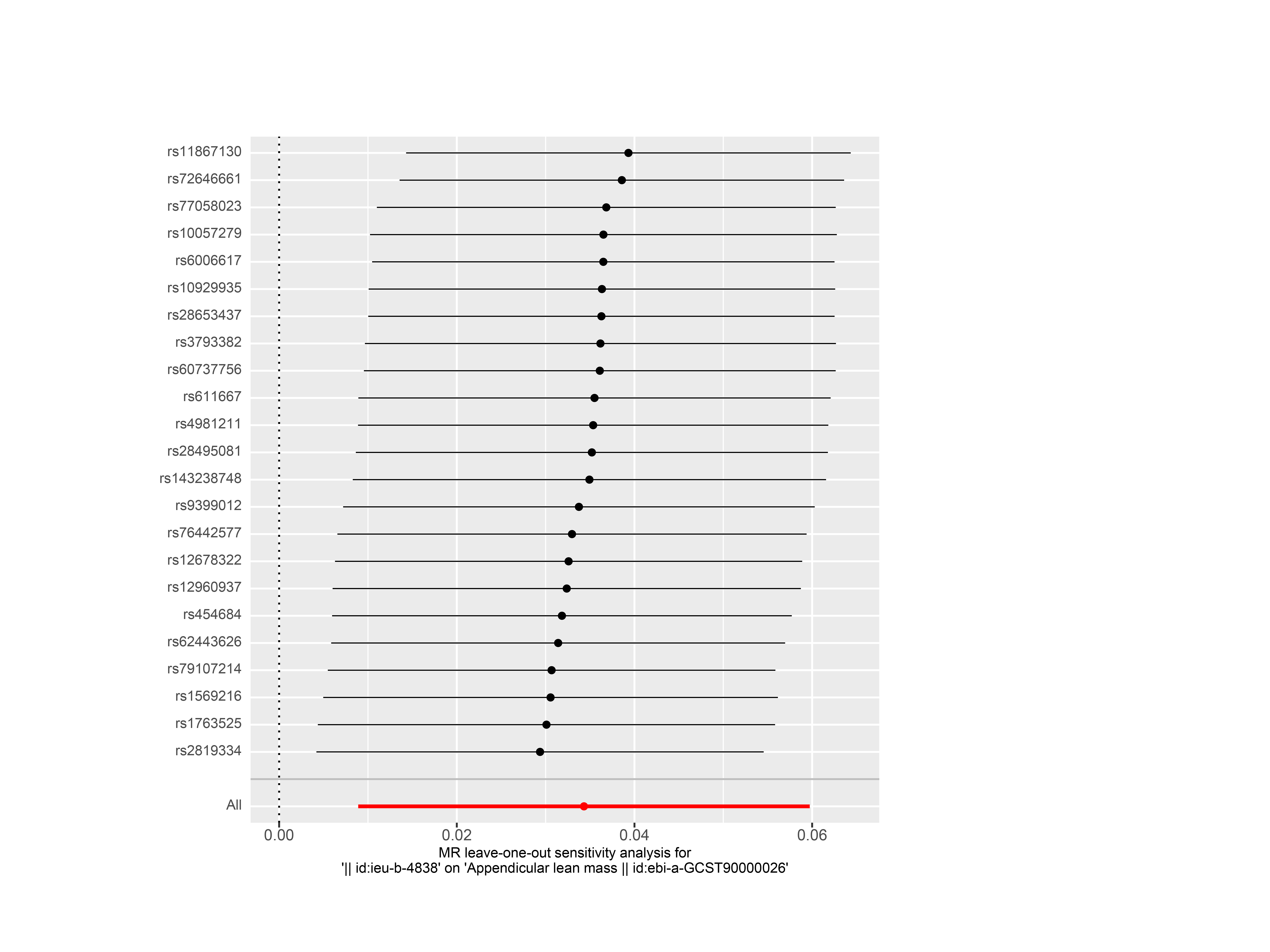

Supplement: S1 Data — (ZIP) [file pone.0309124.s002.zip › Data Sheet/Additional file 4 Leave-one-out sensitivity analysis/H14 Cognitive function on ALM-M.tif]

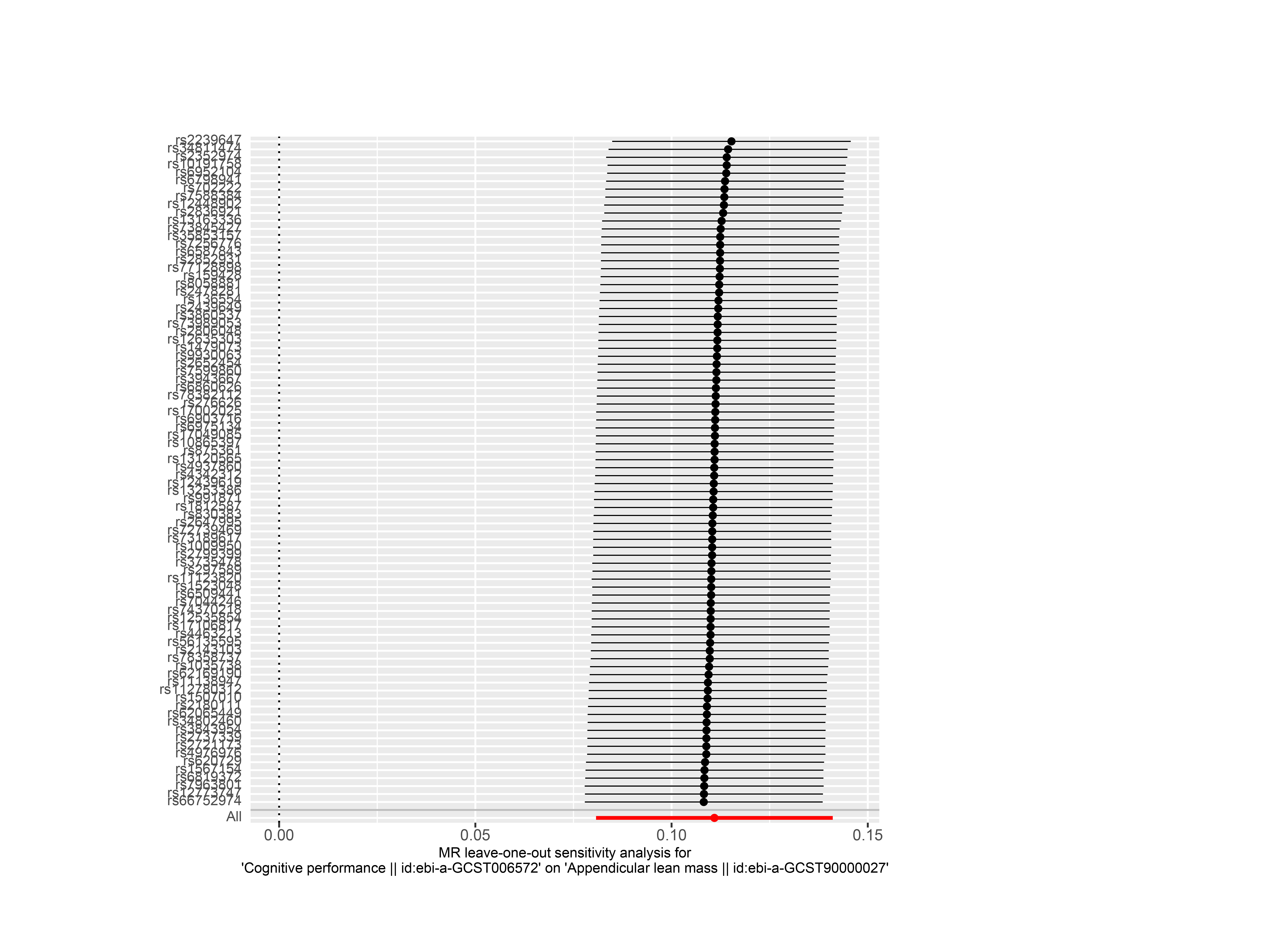

Supplement: S1 Data — (ZIP) [file pone.0309124.s002.zip › Data Sheet/Additional file 4 Leave-one-out sensitivity analysis/H15 Cognitive performance on ALM-F.tif]

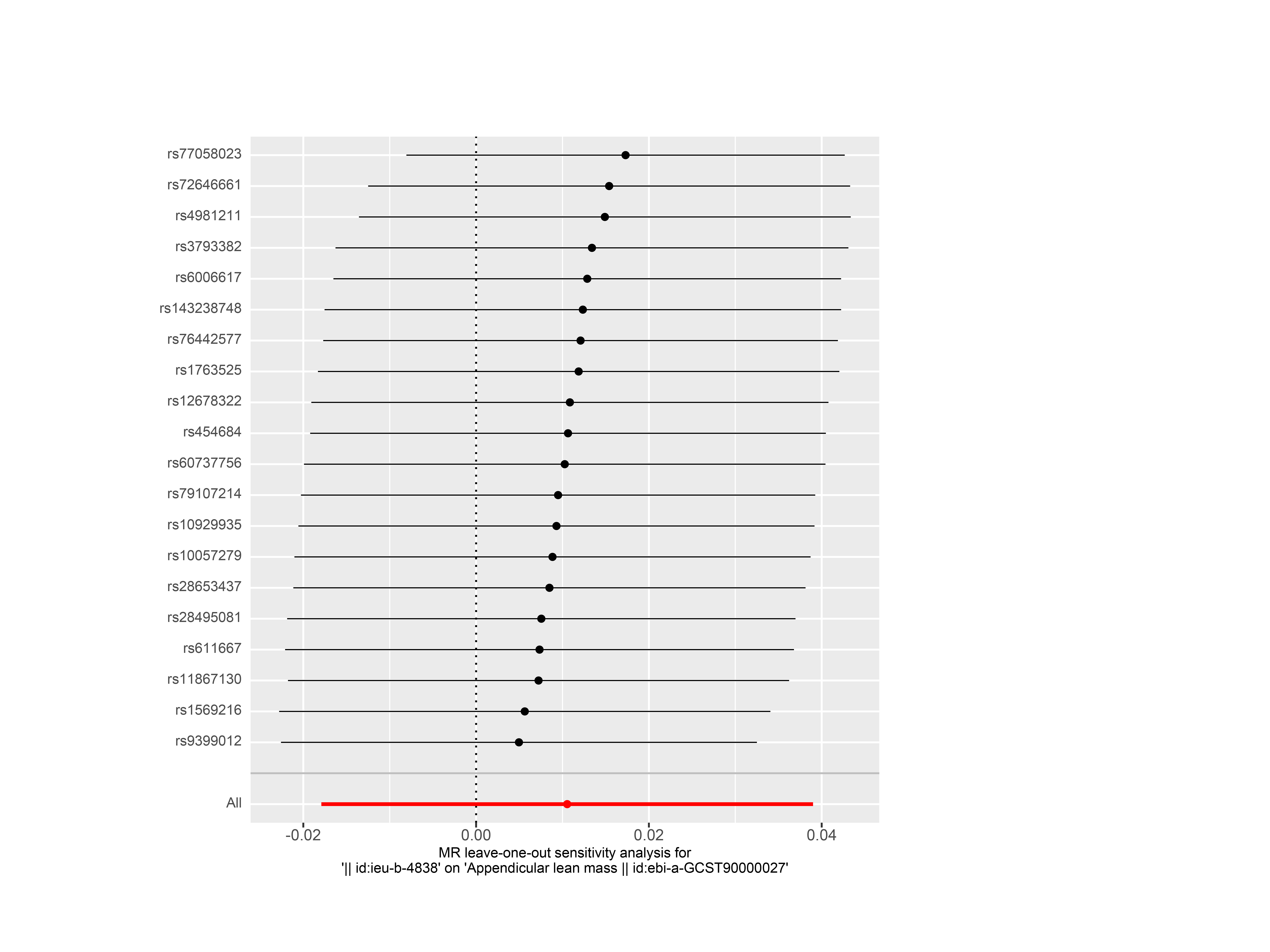

Supplement: S1 Data — (ZIP) [file pone.0309124.s002.zip › Data Sheet/Additional file 4 Leave-one-out sensitivity analysis/H16 Cognitive function on ALM-F.tif]

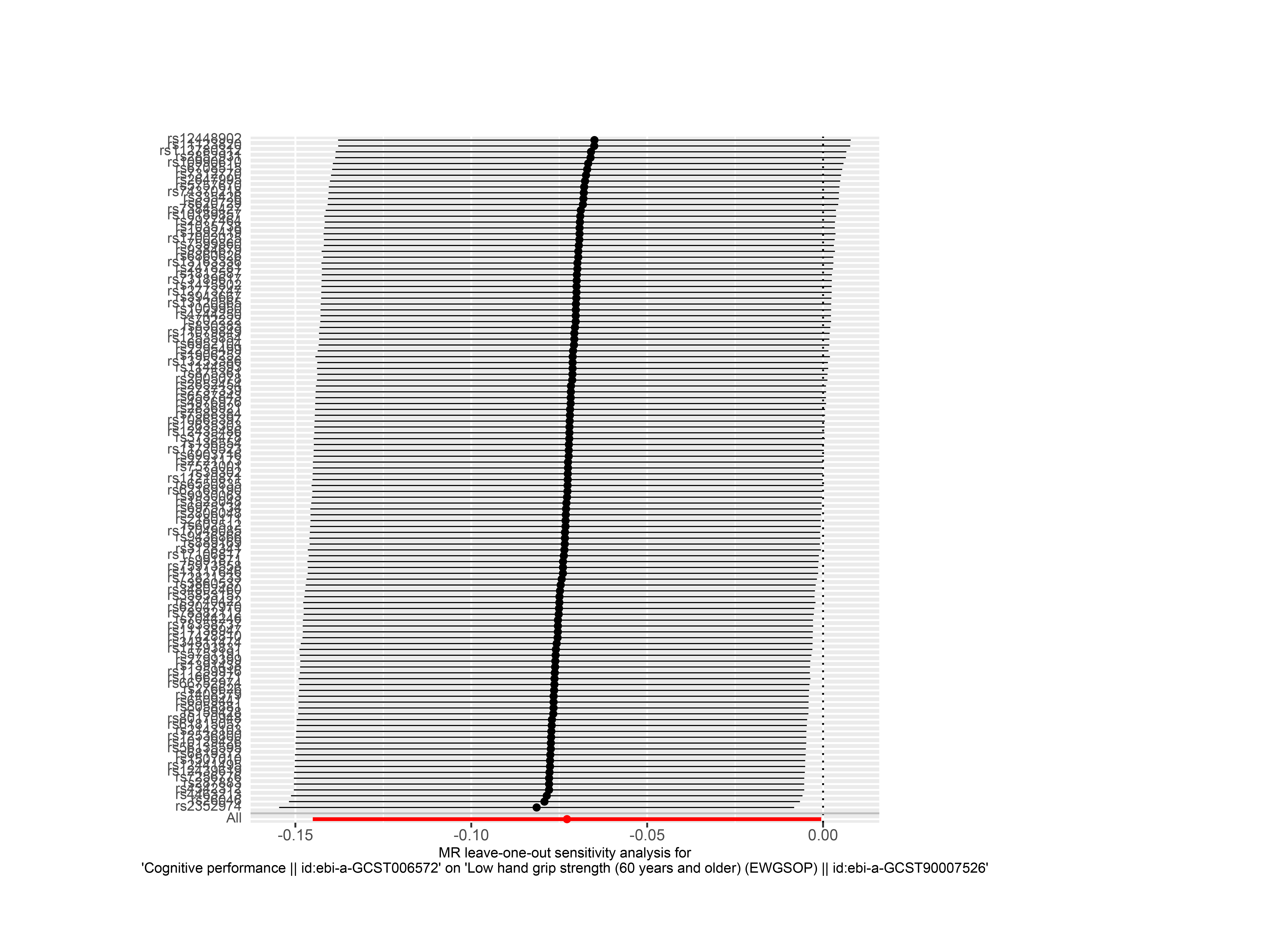

Supplement: S1 Data — (ZIP) [file pone.0309124.s002.zip › Data Sheet/Additional file 4 Leave-one-out sensitivity analysis/H17 Cognitive performance on low hand grip strength.tif]

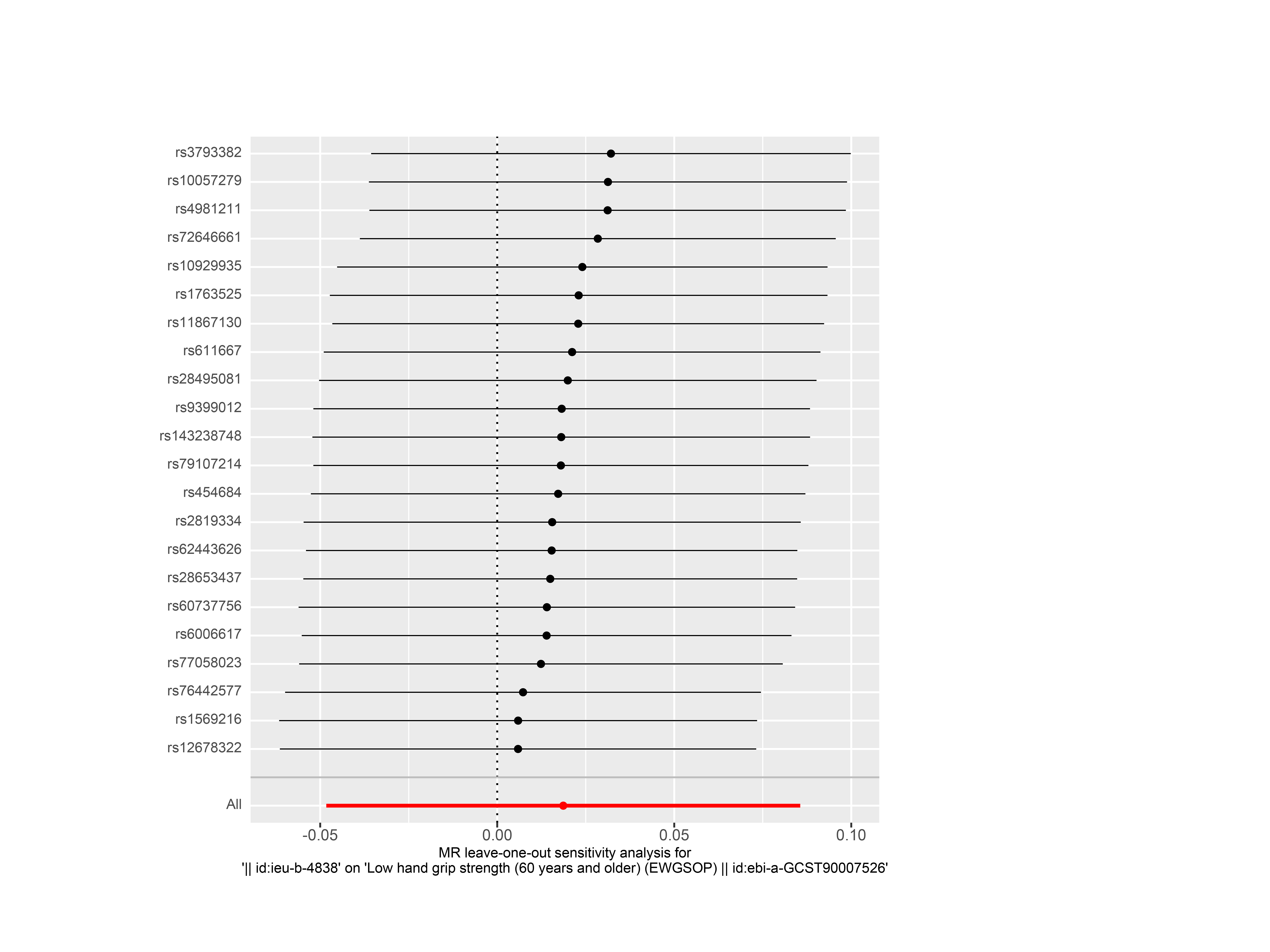

Supplement: S1 Data — (ZIP) [file pone.0309124.s002.zip › Data Sheet/Additional file 4 Leave-one-out sensitivity analysis/H18 Cognitive function on low hand grip strength.tif]

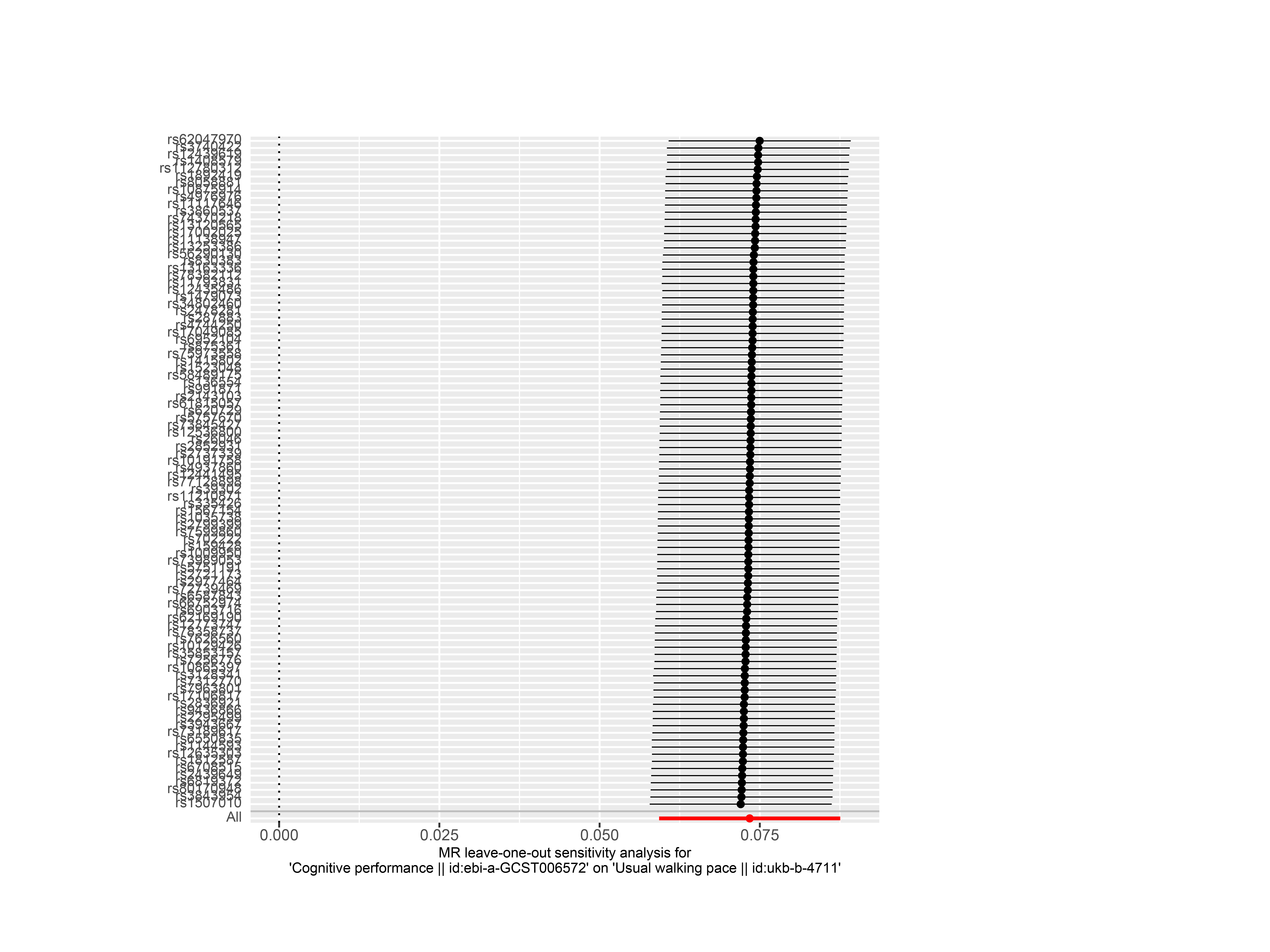

Supplement: S1 Data — (ZIP) [file pone.0309124.s002.zip › Data Sheet/Additional file 4 Leave-one-out sensitivity analysis/H19 Cognitive performance on walking pace.tif]

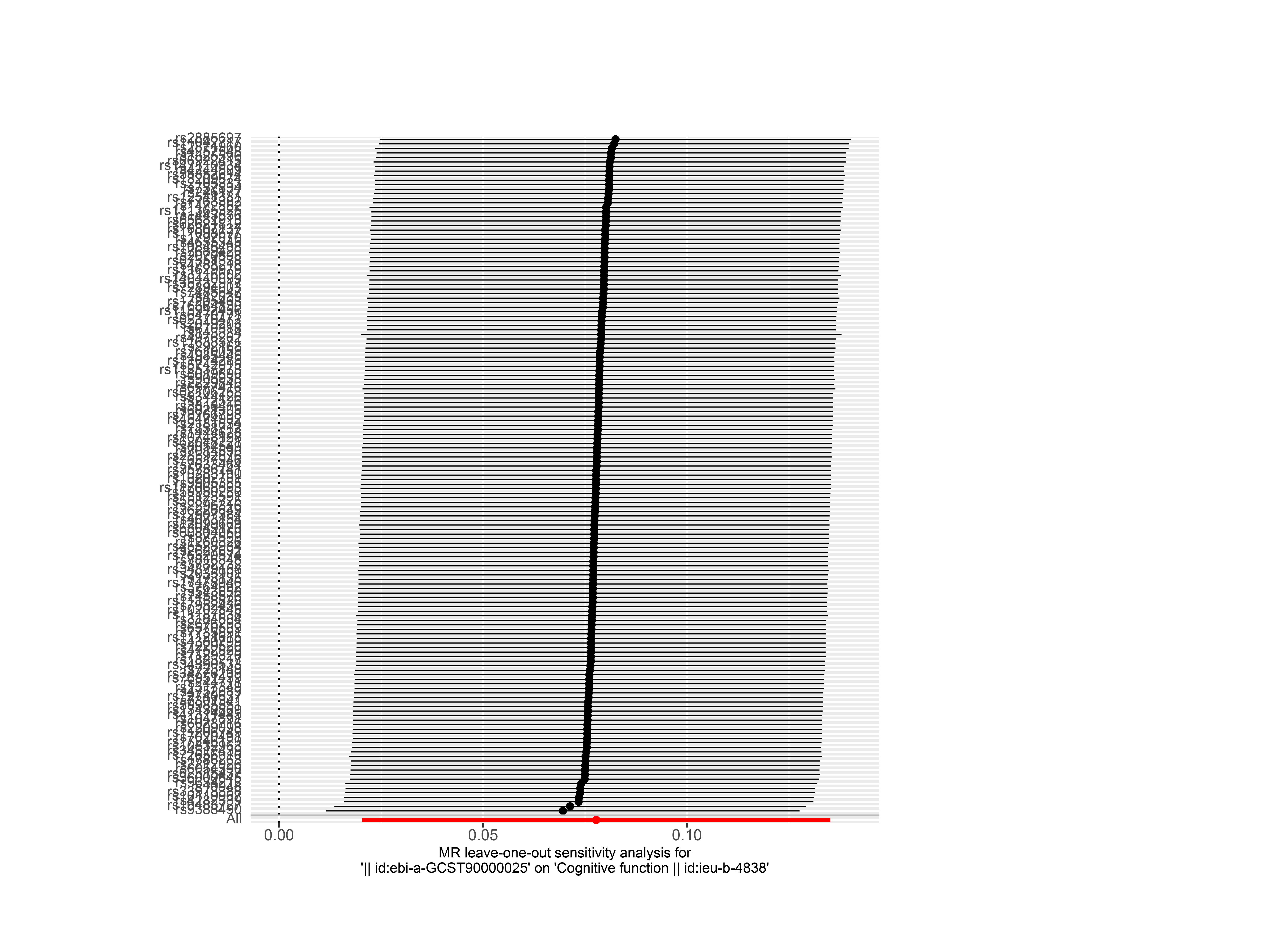

Supplement: S1 Data — (ZIP) [file pone.0309124.s002.zip › Data Sheet/Additional file 4 Leave-one-out sensitivity analysis/H2 ALM on cognitive function.tif]

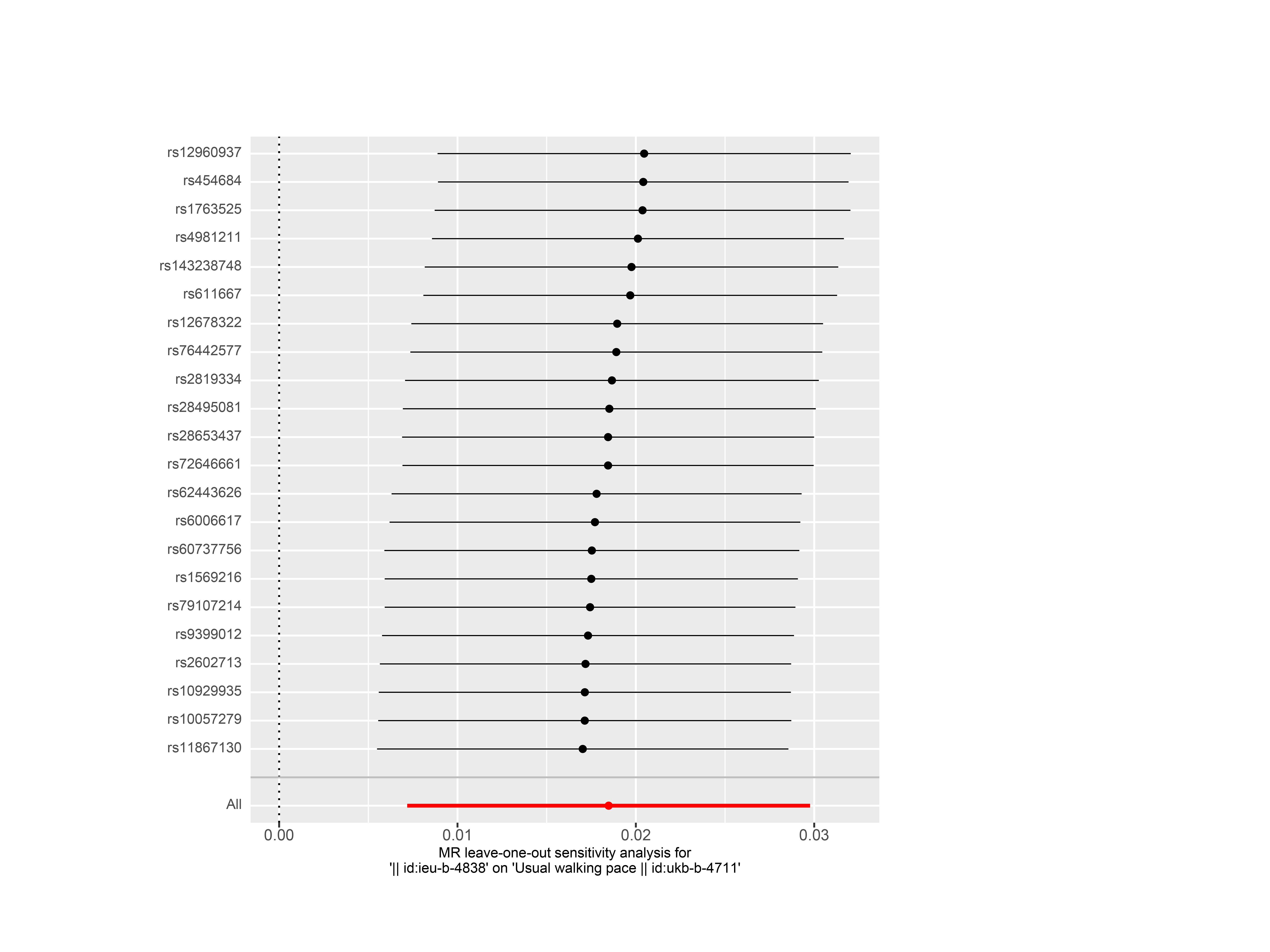

Supplement: S1 Data — (ZIP) [file pone.0309124.s002.zip › Data Sheet/Additional file 4 Leave-one-out sensitivity analysis/H20 Cognitive function on walking pace.tif]

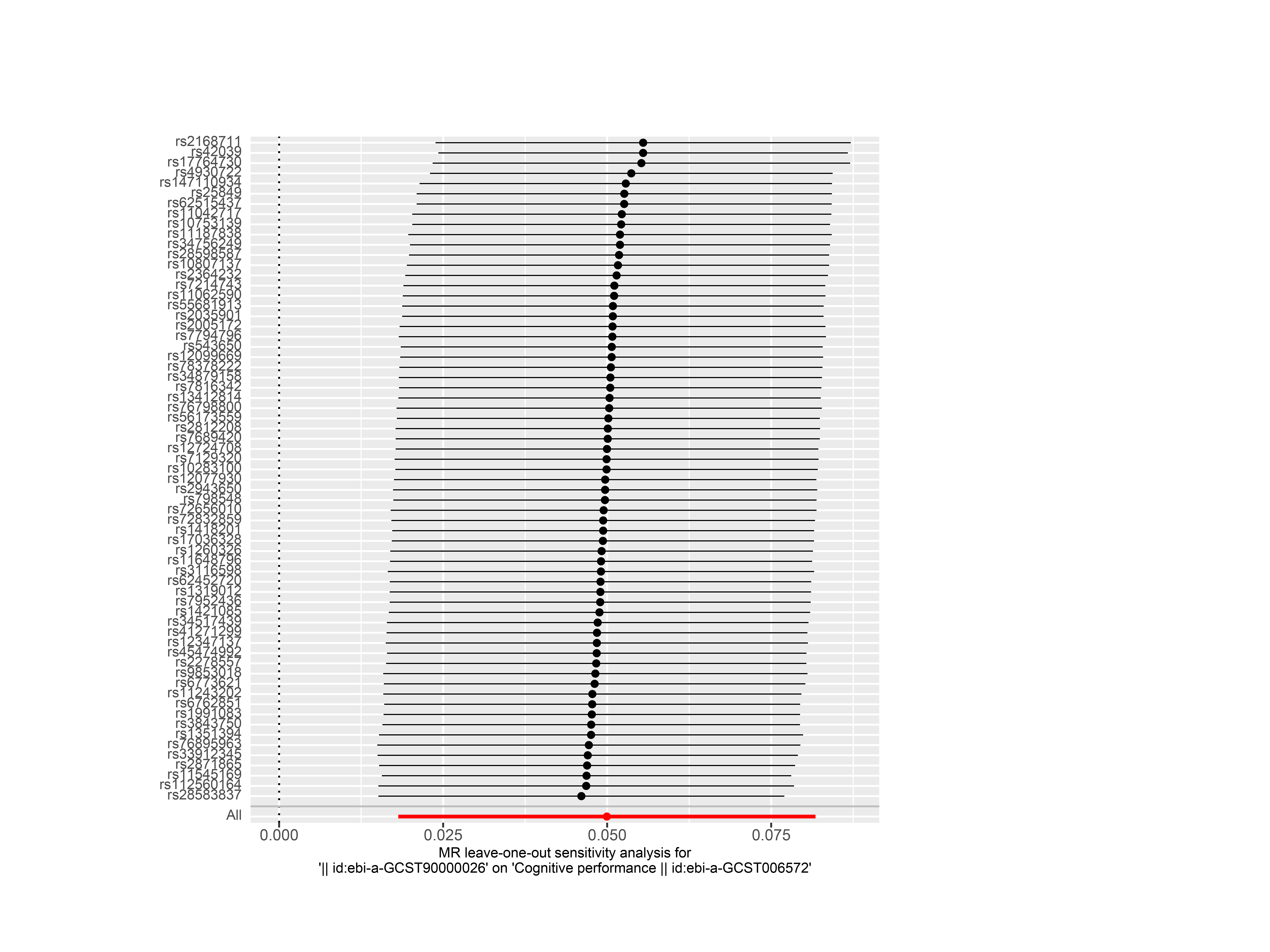

Supplement: S1 Data — (ZIP) [file pone.0309124.s002.zip › Data Sheet/Additional file 4 Leave-one-out sensitivity analysis/H3 ALM-M on cognitive performance.tif]

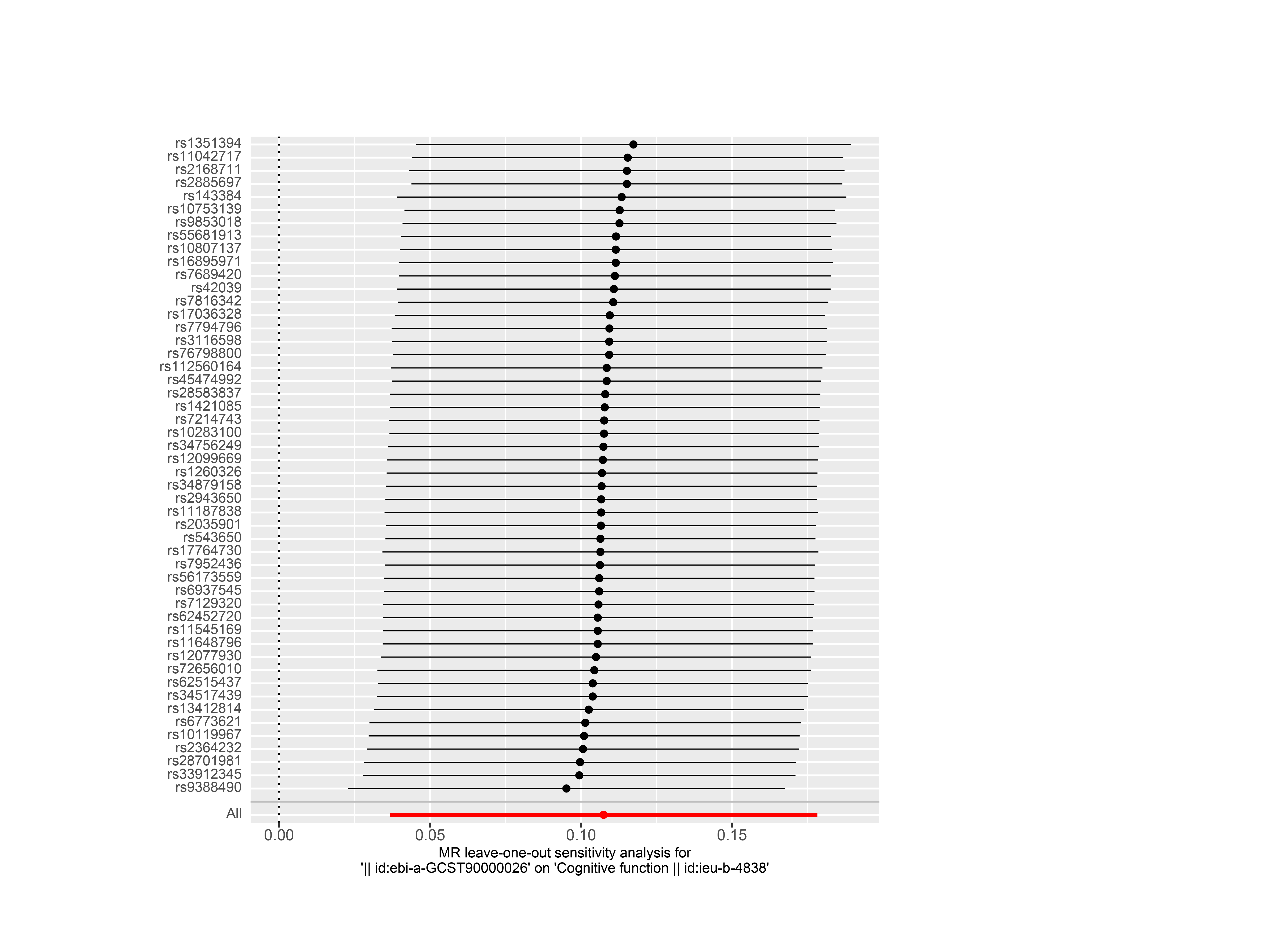

Supplement: S1 Data — (ZIP) [file pone.0309124.s002.zip › Data Sheet/Additional file 4 Leave-one-out sensitivity analysis/H4 ALM-M on cognitive function.tif]

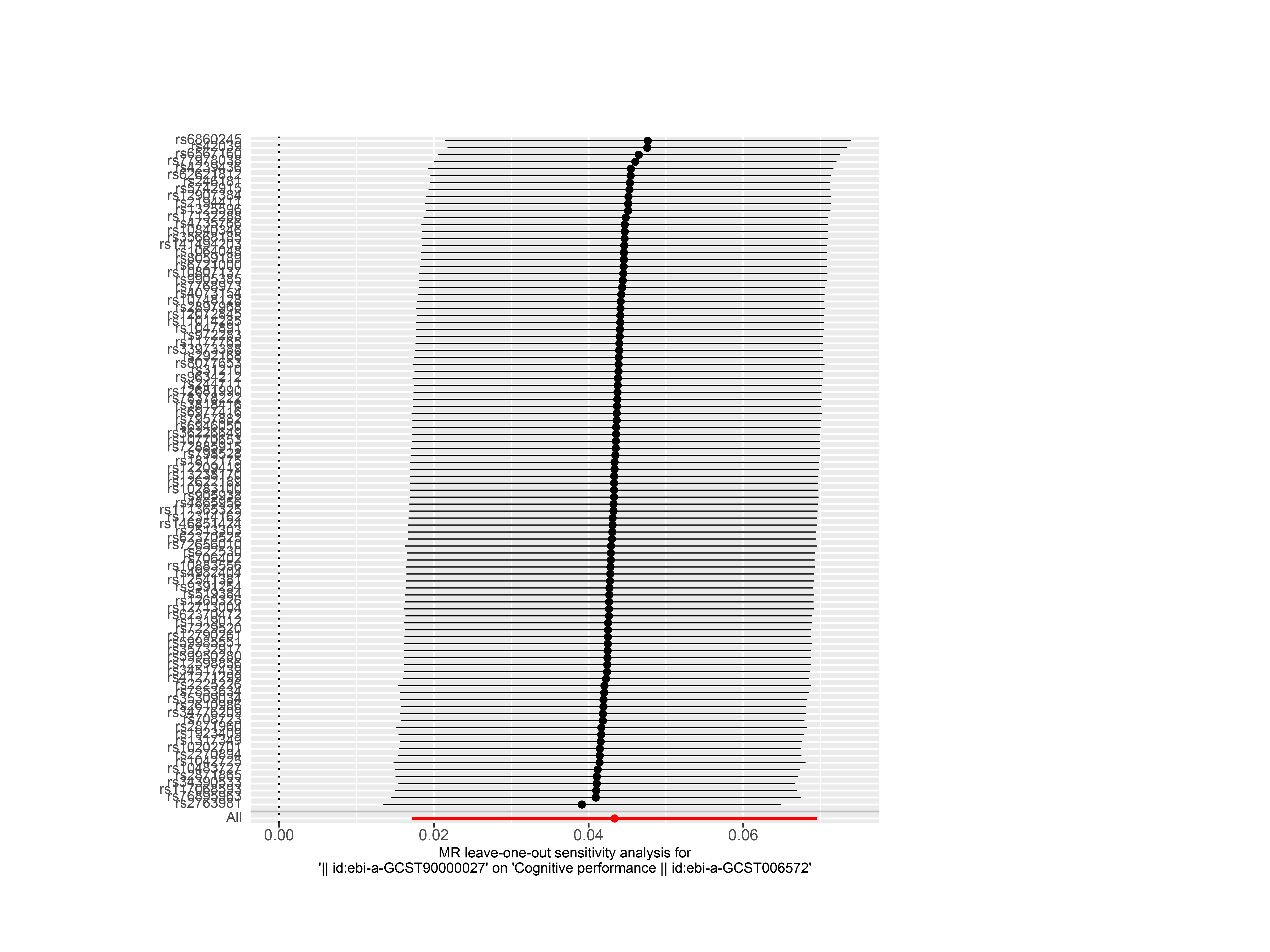

Supplement: S1 Data — (ZIP) [file pone.0309124.s002.zip › Data Sheet/Additional file 4 Leave-one-out sensitivity analysis/H5 ALM-F on cognitive performance.tif]

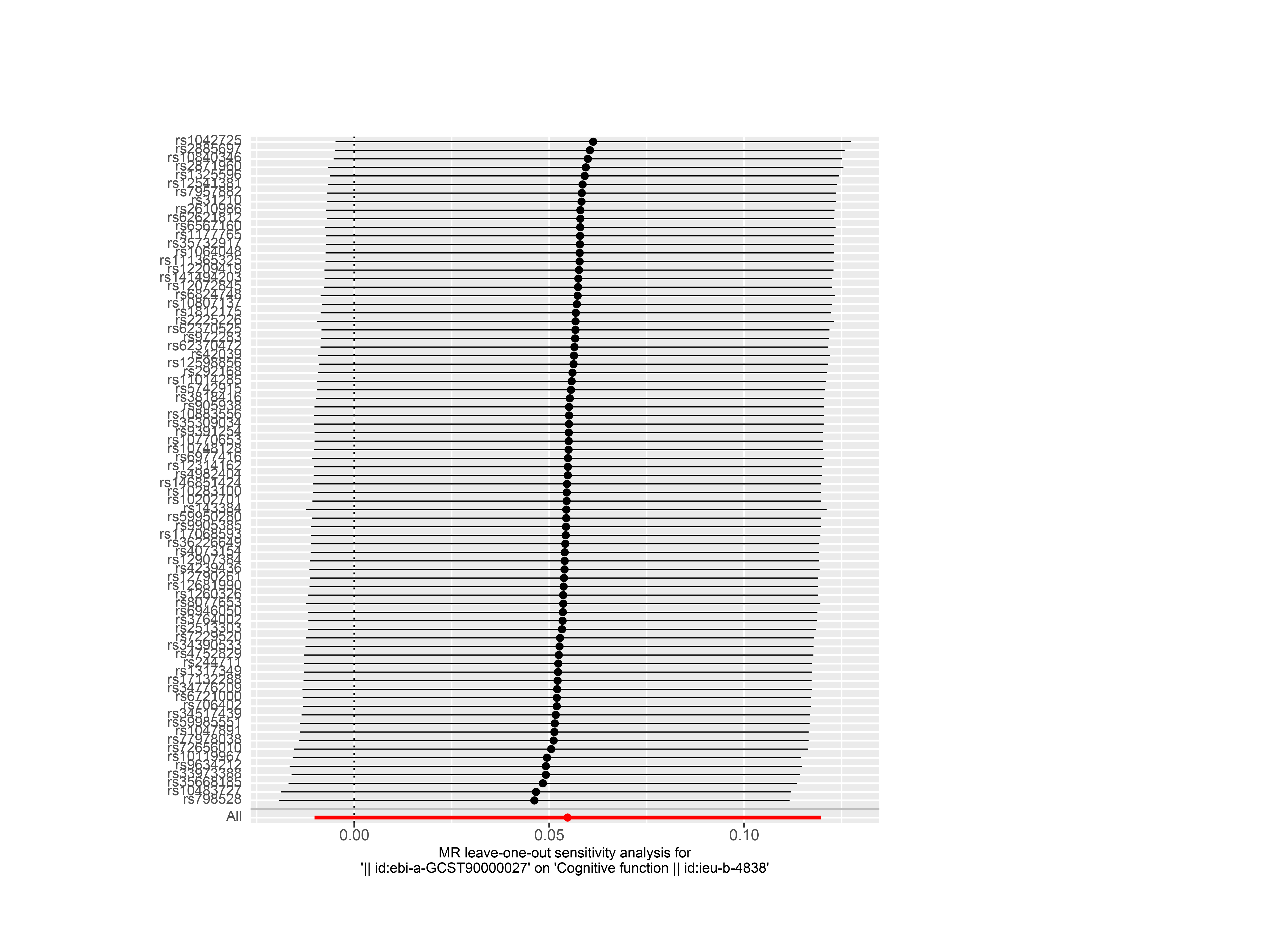

Supplement: S1 Data — (ZIP) [file pone.0309124.s002.zip › Data Sheet/Additional file 4 Leave-one-out sensitivity analysis/H6 ALM-F on cognitive function.tif]

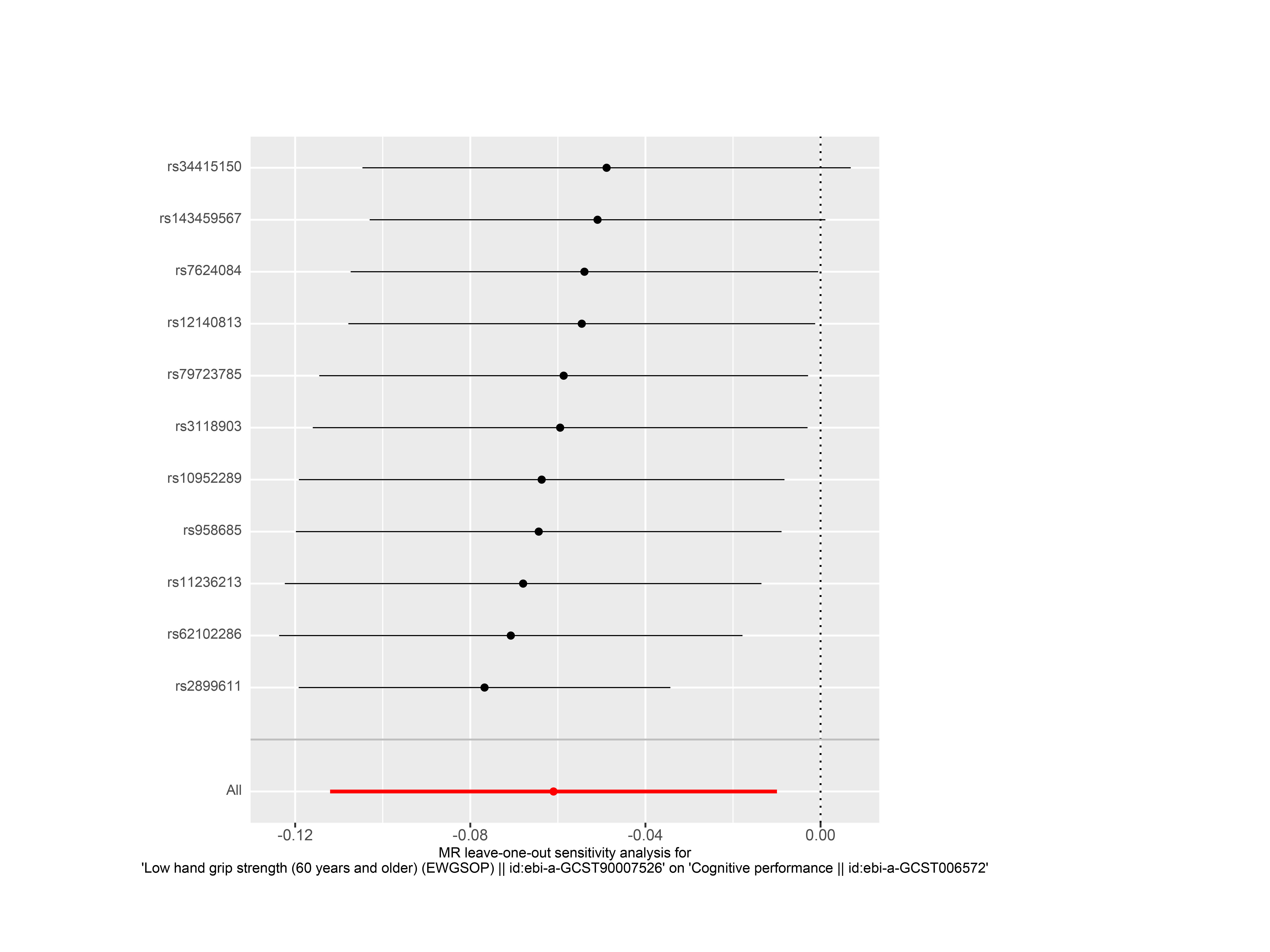

Supplement: S1 Data — (ZIP) [file pone.0309124.s002.zip › Data Sheet/Additional file 4 Leave-one-out sensitivity analysis/H7 Low hand grip strength on cognitive performance.tif]

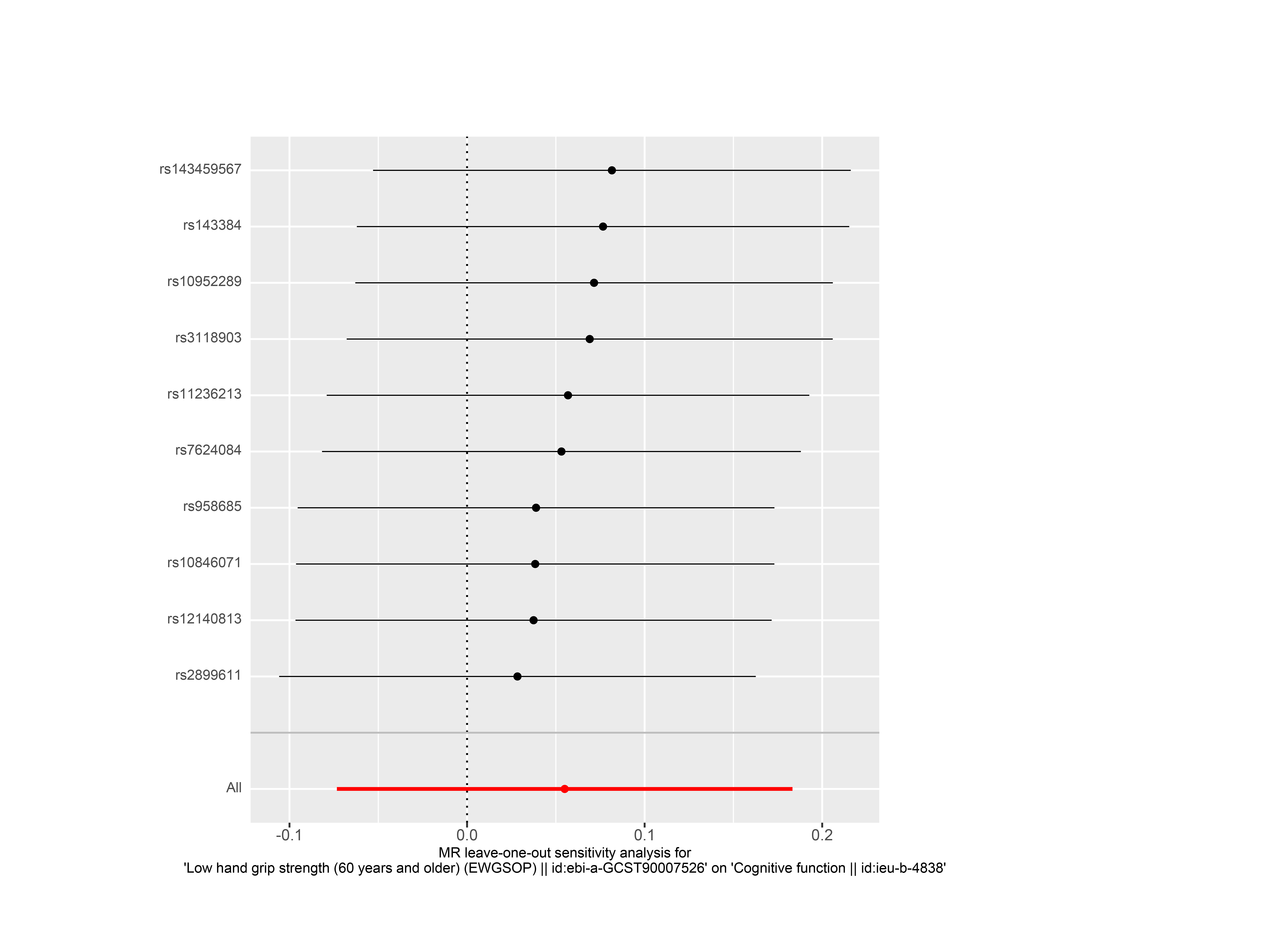

Supplement: S1 Data — (ZIP) [file pone.0309124.s002.zip › Data Sheet/Additional file 4 Leave-one-out sensitivity analysis/H8 Low hand grip strength on cognitive function.tif]

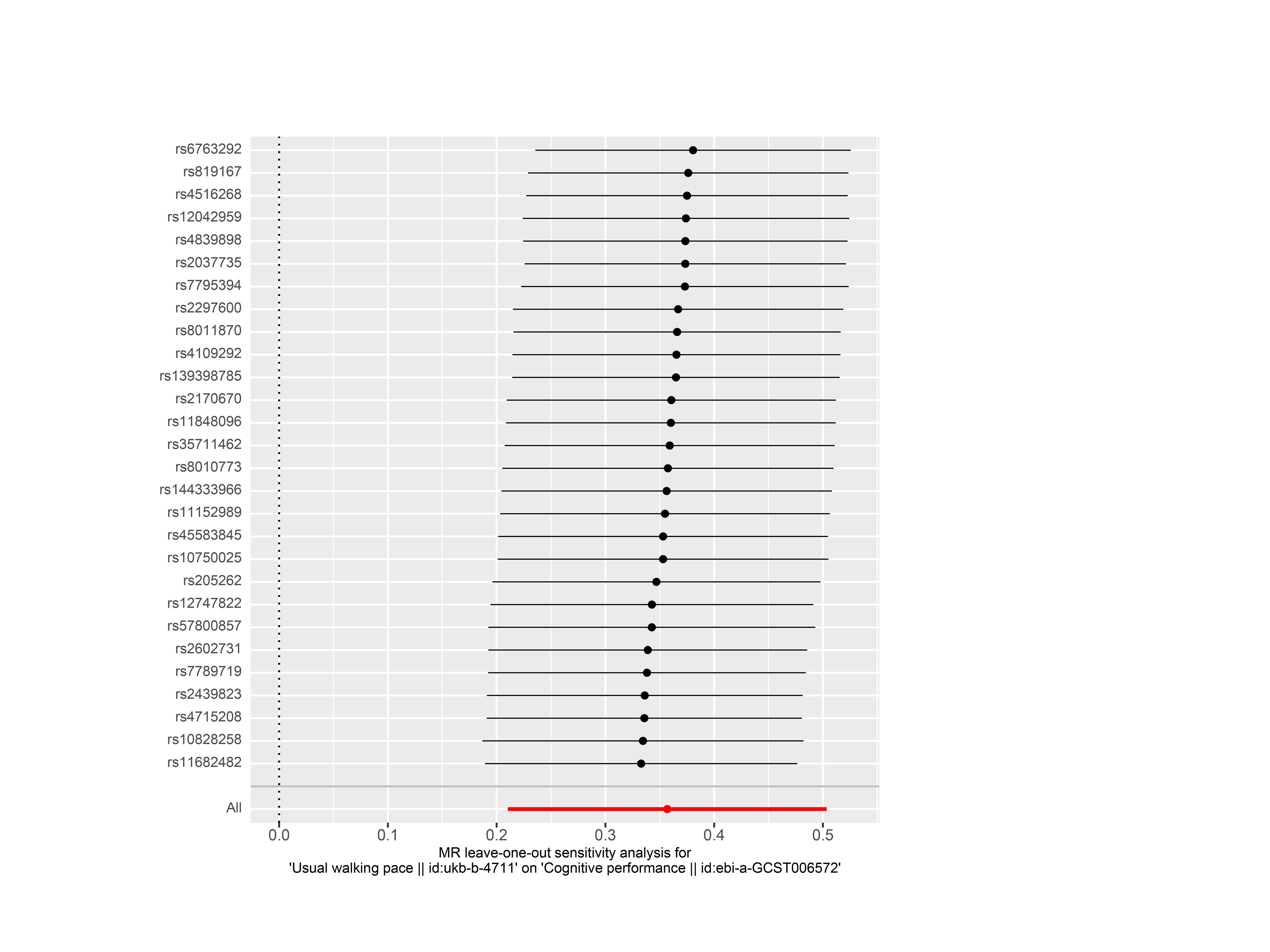

Supplement: S1 Data — (ZIP) [file pone.0309124.s002.zip › Data Sheet/Additional file 4 Leave-one-out sensitivity analysis/H9 Walking pace on cognitive performance.tif]

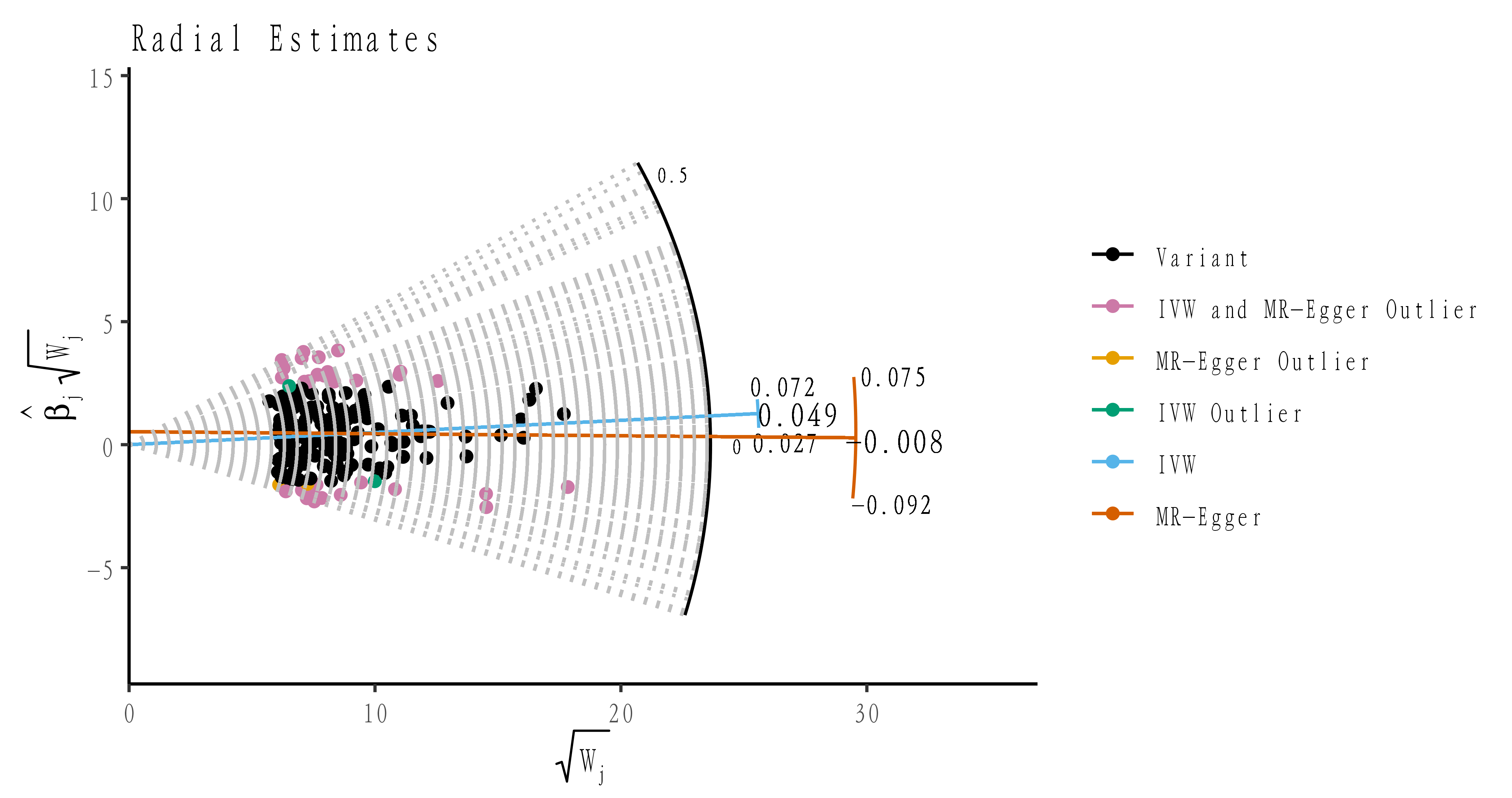

Supplement: S1 Data — (ZIP) [file pone.0309124.s002.zip › Data Sheet/Additional file 5 RadialMR plot figure/K1 ALM on cognitive performance.tif]

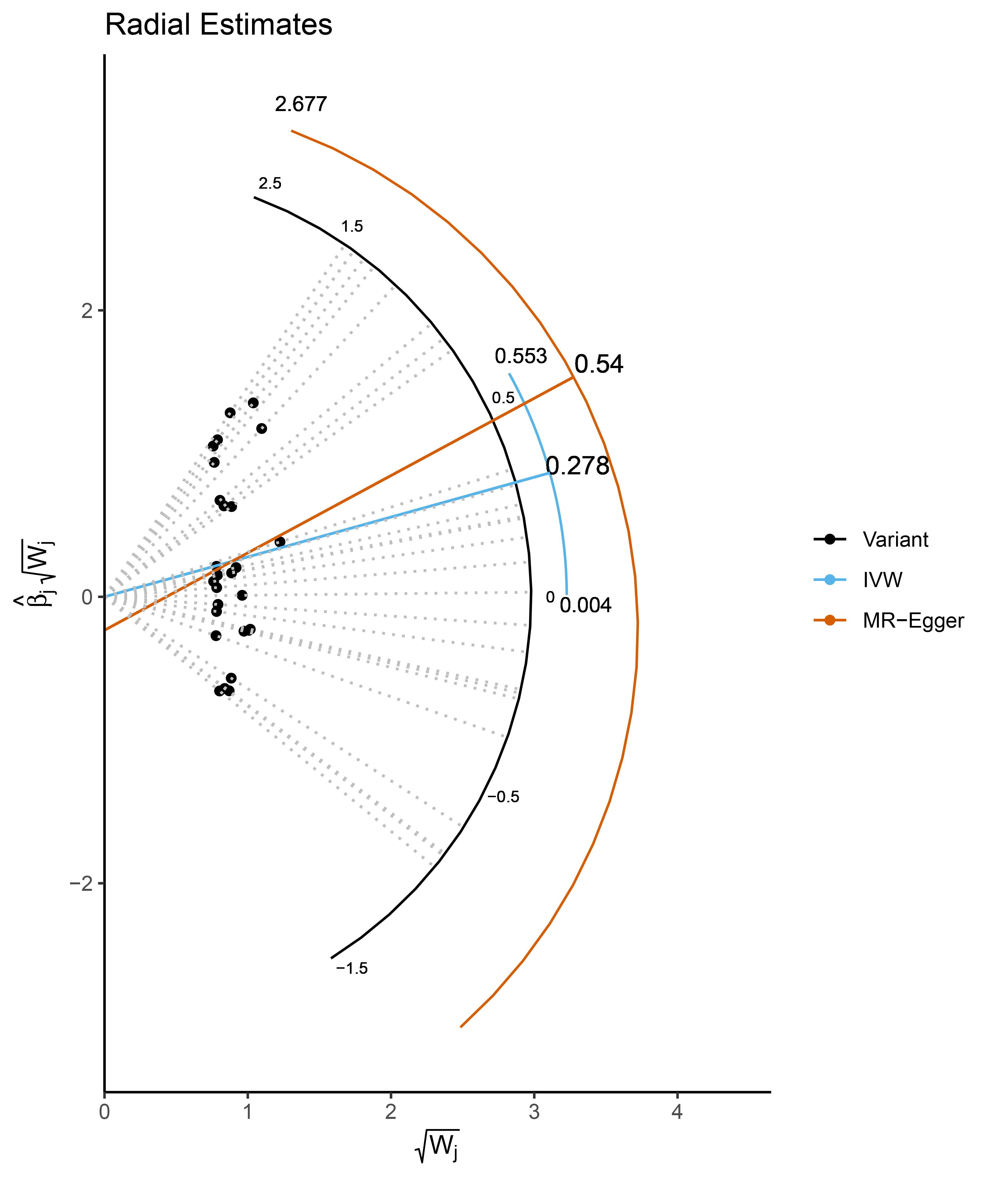

Supplement: S1 Data — (ZIP) [file pone.0309124.s002.zip › Data Sheet/Additional file 5 RadialMR plot figure/K10 Walking pace on cognitive function.tif]

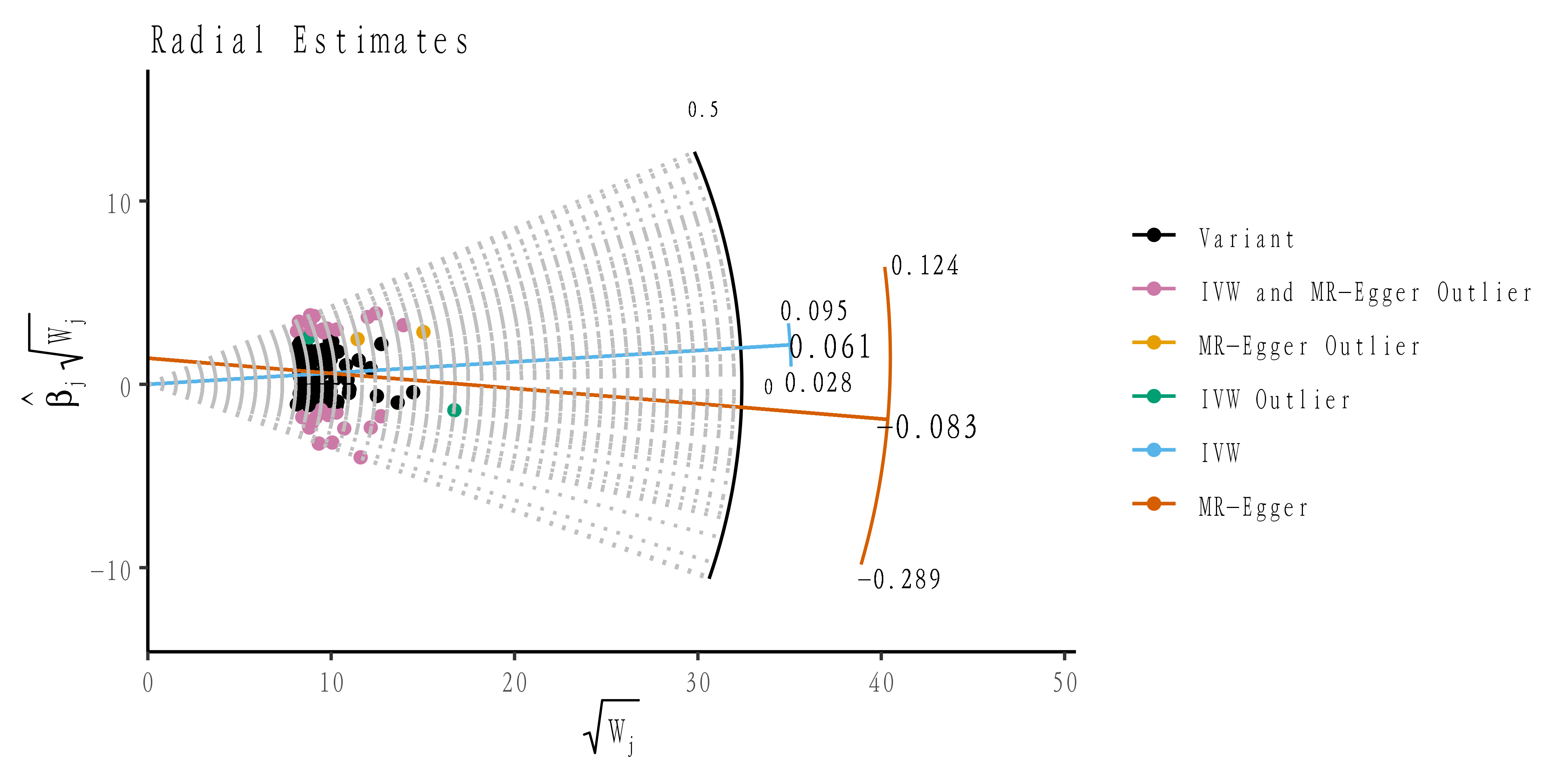

Supplement: S1 Data — (ZIP) [file pone.0309124.s002.zip › Data Sheet/Additional file 5 RadialMR plot figure/K11 Cognitive performance on ALM.tif]

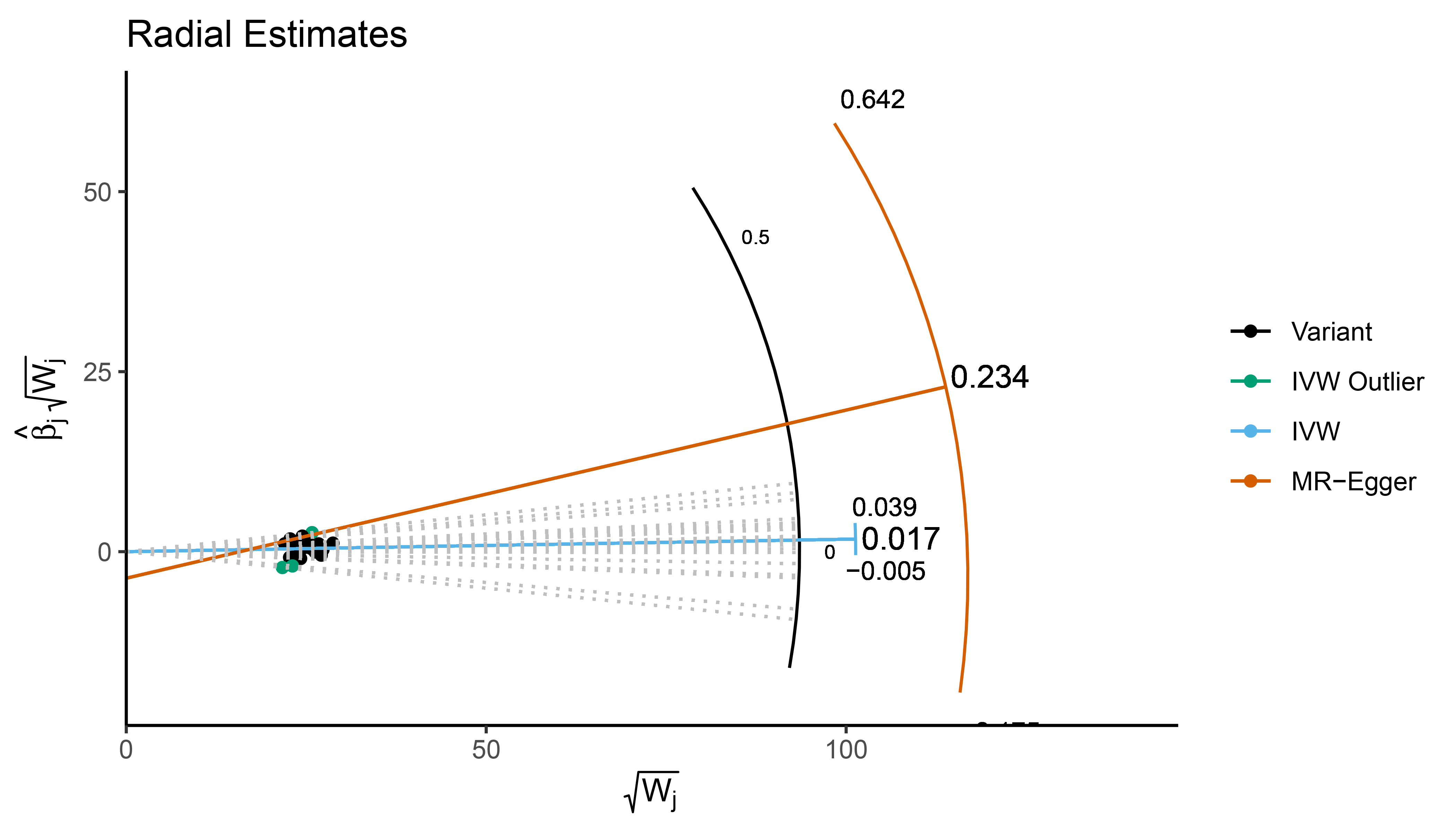

Supplement: S1 Data — (ZIP) [file pone.0309124.s002.zip › Data Sheet/Additional file 5 RadialMR plot figure/K12 Cognitive function on ALM.tif]

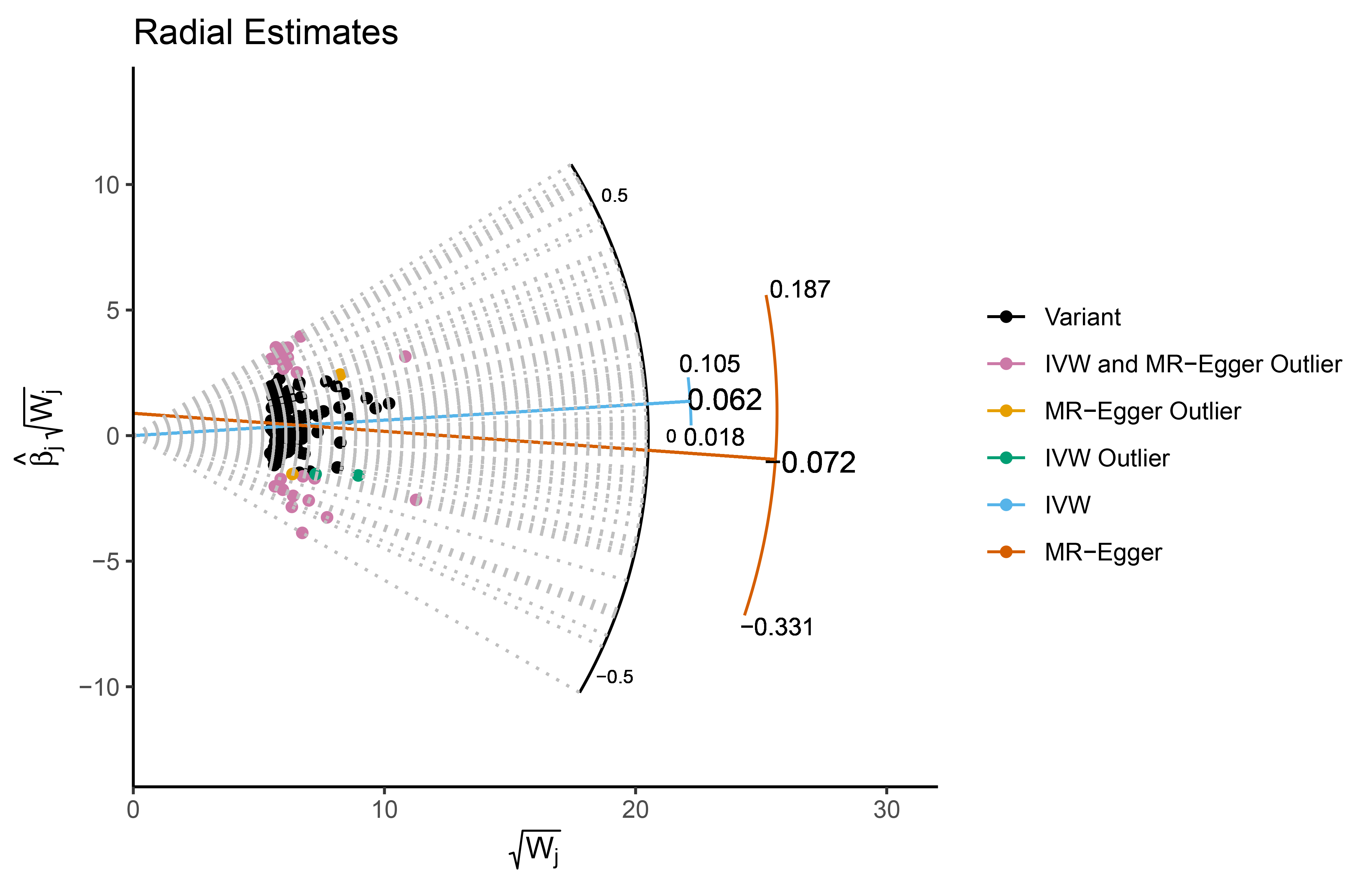

Supplement: S1 Data — (ZIP) [file pone.0309124.s002.zip › Data Sheet/Additional file 5 RadialMR plot figure/K13 Cognitive performance on ALM-M.tif]

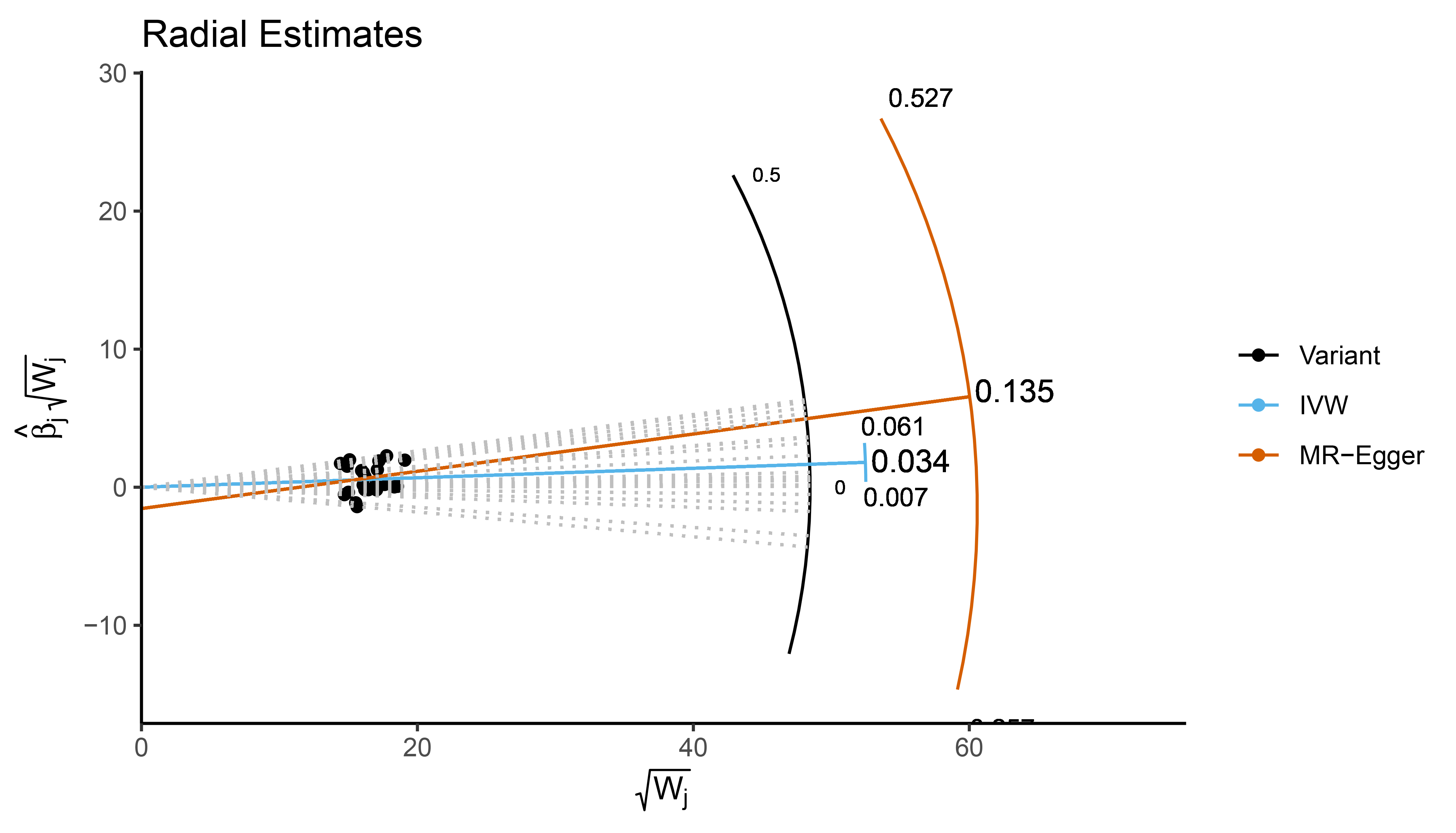

Supplement: S1 Data — (ZIP) [file pone.0309124.s002.zip › Data Sheet/Additional file 5 RadialMR plot figure/K14 Cognitive function on ALM-M.tif]

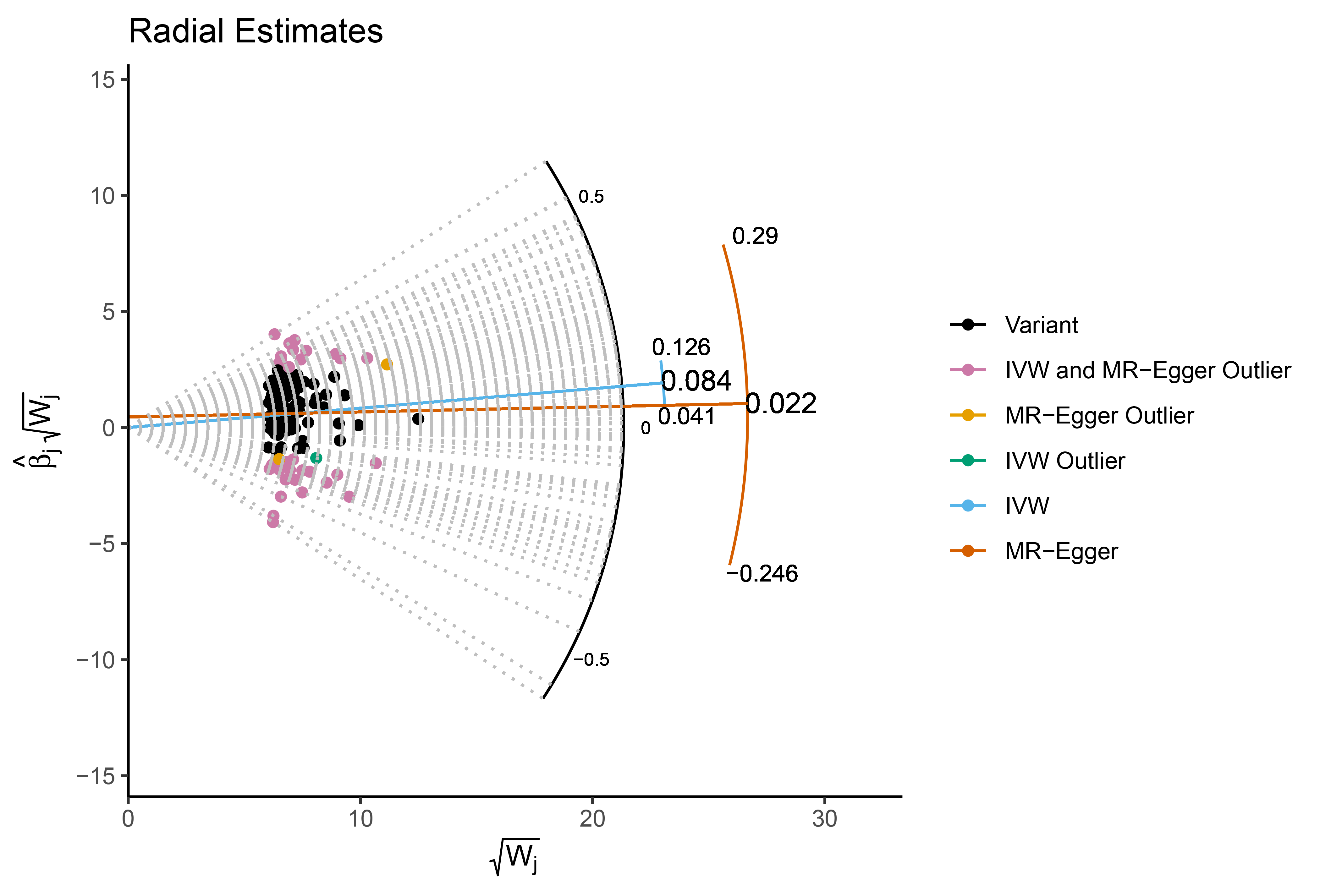

Supplement: S1 Data — (ZIP) [file pone.0309124.s002.zip › Data Sheet/Additional file 5 RadialMR plot figure/K15 Cognitive performance on ALM-F.tif]

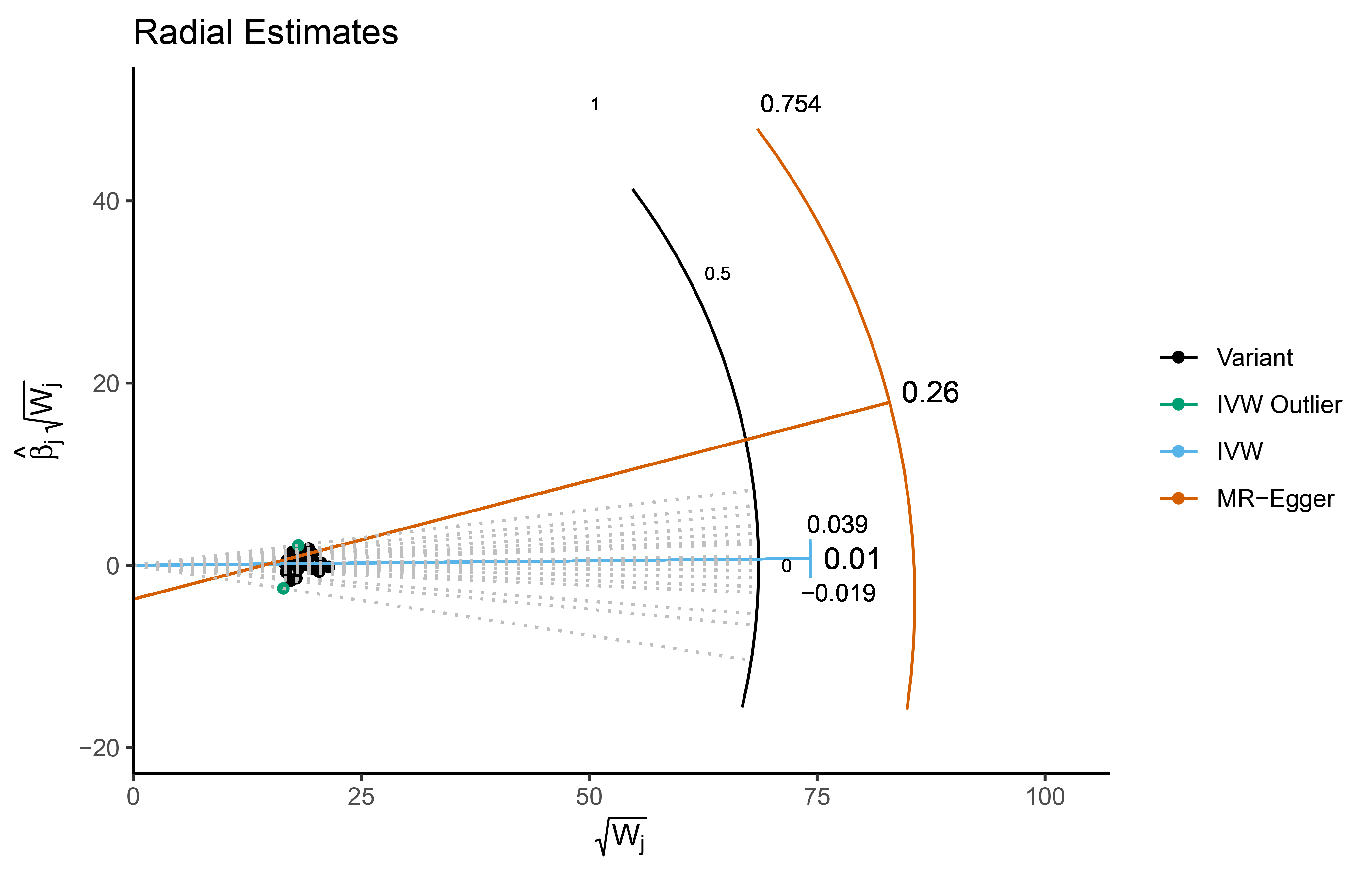

Supplement: S1 Data — (ZIP) [file pone.0309124.s002.zip › Data Sheet/Additional file 5 RadialMR plot figure/K16 Cognitive function on ALM-F.tif]

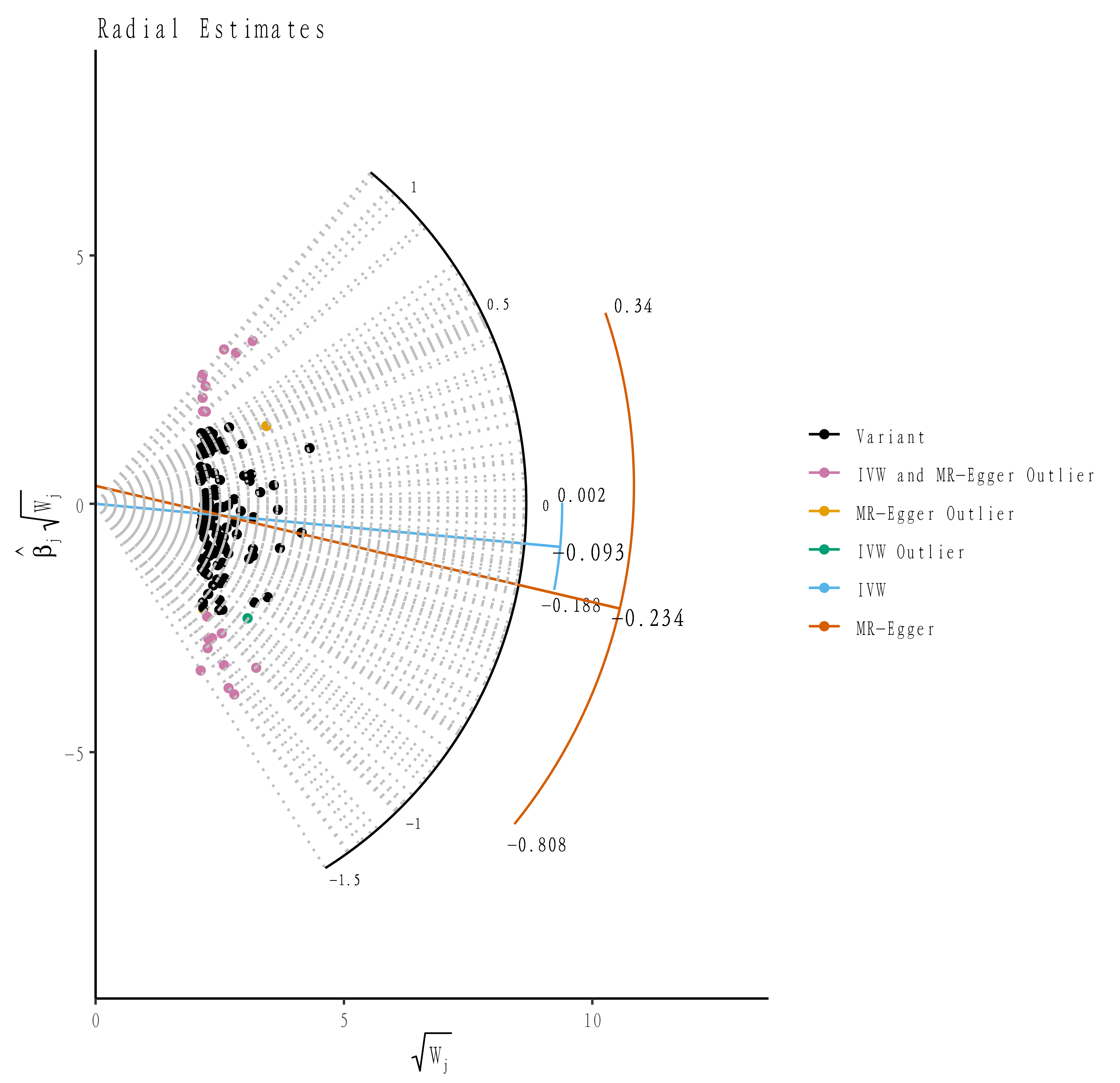

Supplement: S1 Data — (ZIP) [file pone.0309124.s002.zip › Data Sheet/Additional file 5 RadialMR plot figure/K17 Cognitive performance on low hand grip strength.tif]

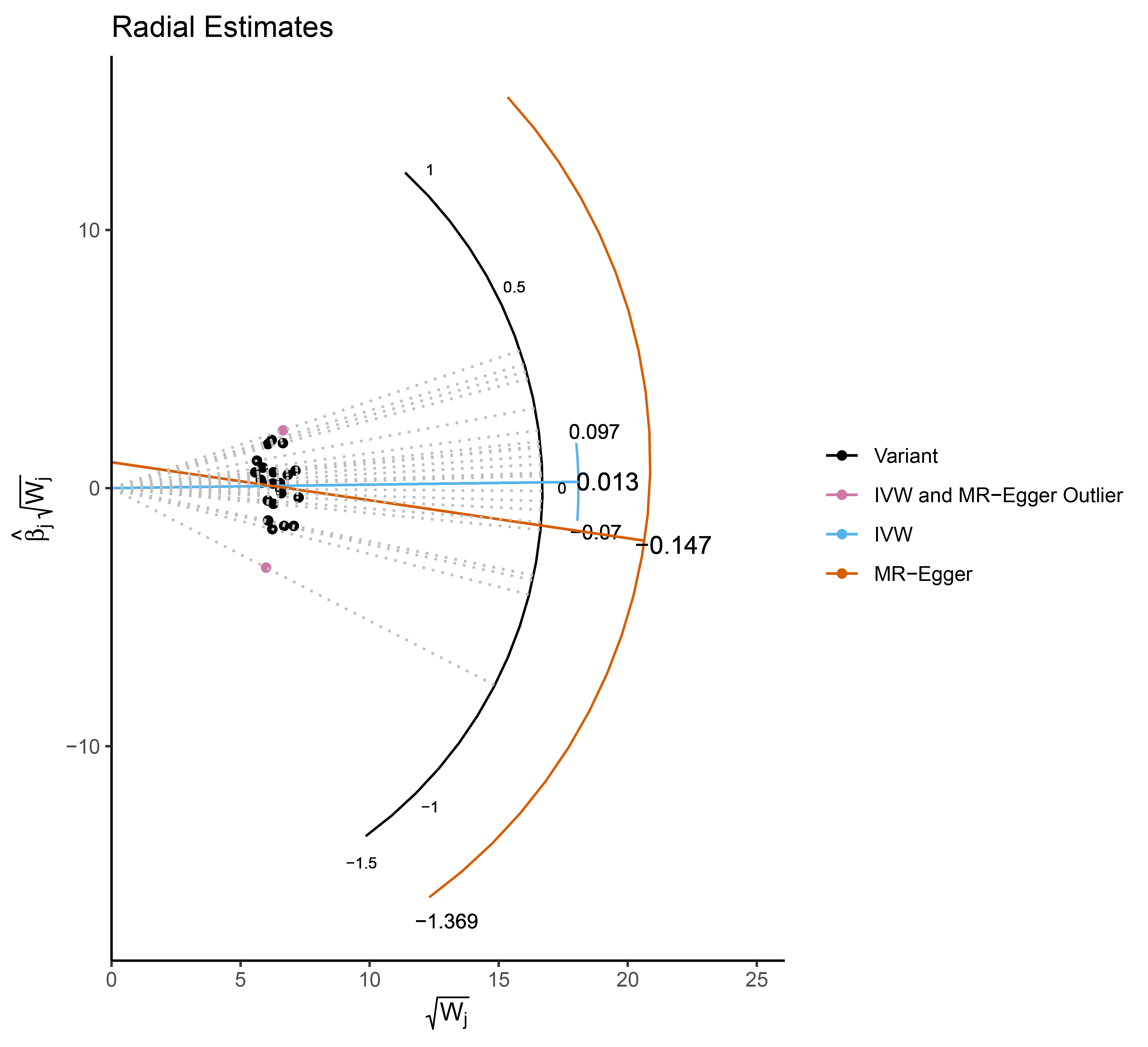

Supplement: S1 Data — (ZIP) [file pone.0309124.s002.zip › Data Sheet/Additional file 5 RadialMR plot figure/K18 Cognitive function on low hand grip strength.tif]

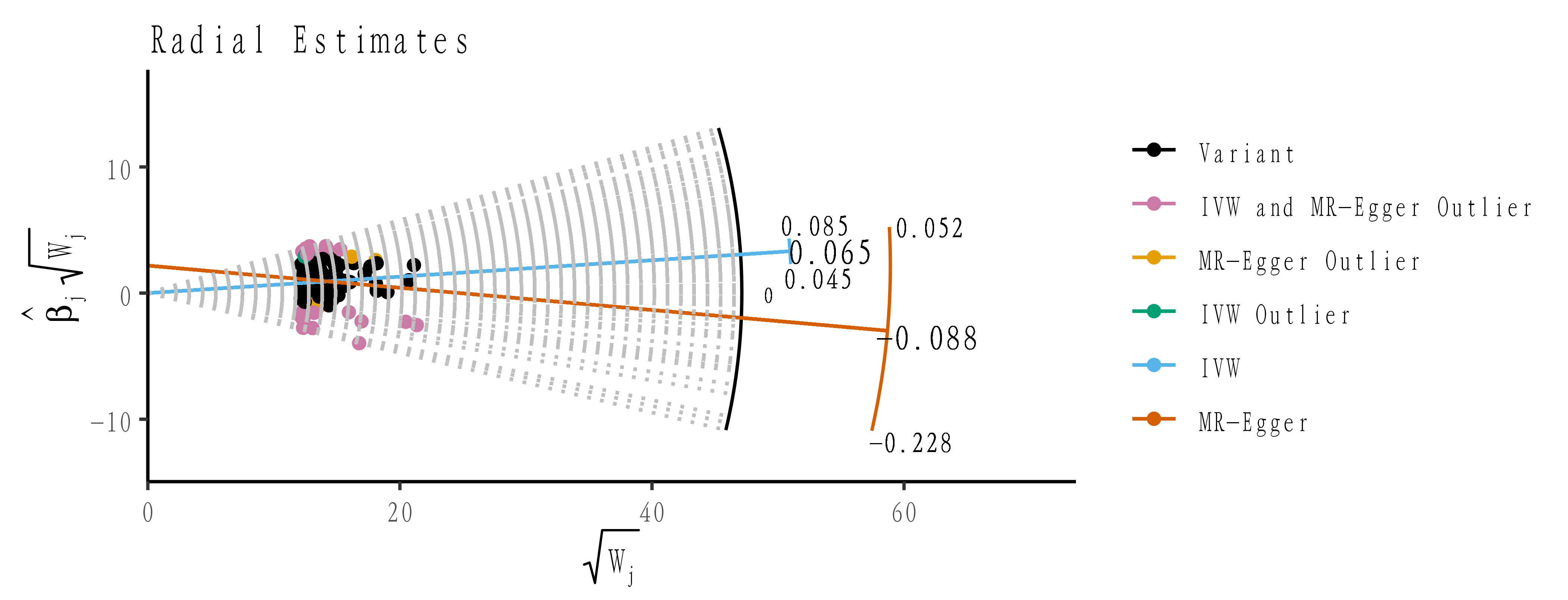

Supplement: S1 Data — (ZIP) [file pone.0309124.s002.zip › Data Sheet/Additional file 5 RadialMR plot figure/K19 Cognitive performance on walking pace.tif]

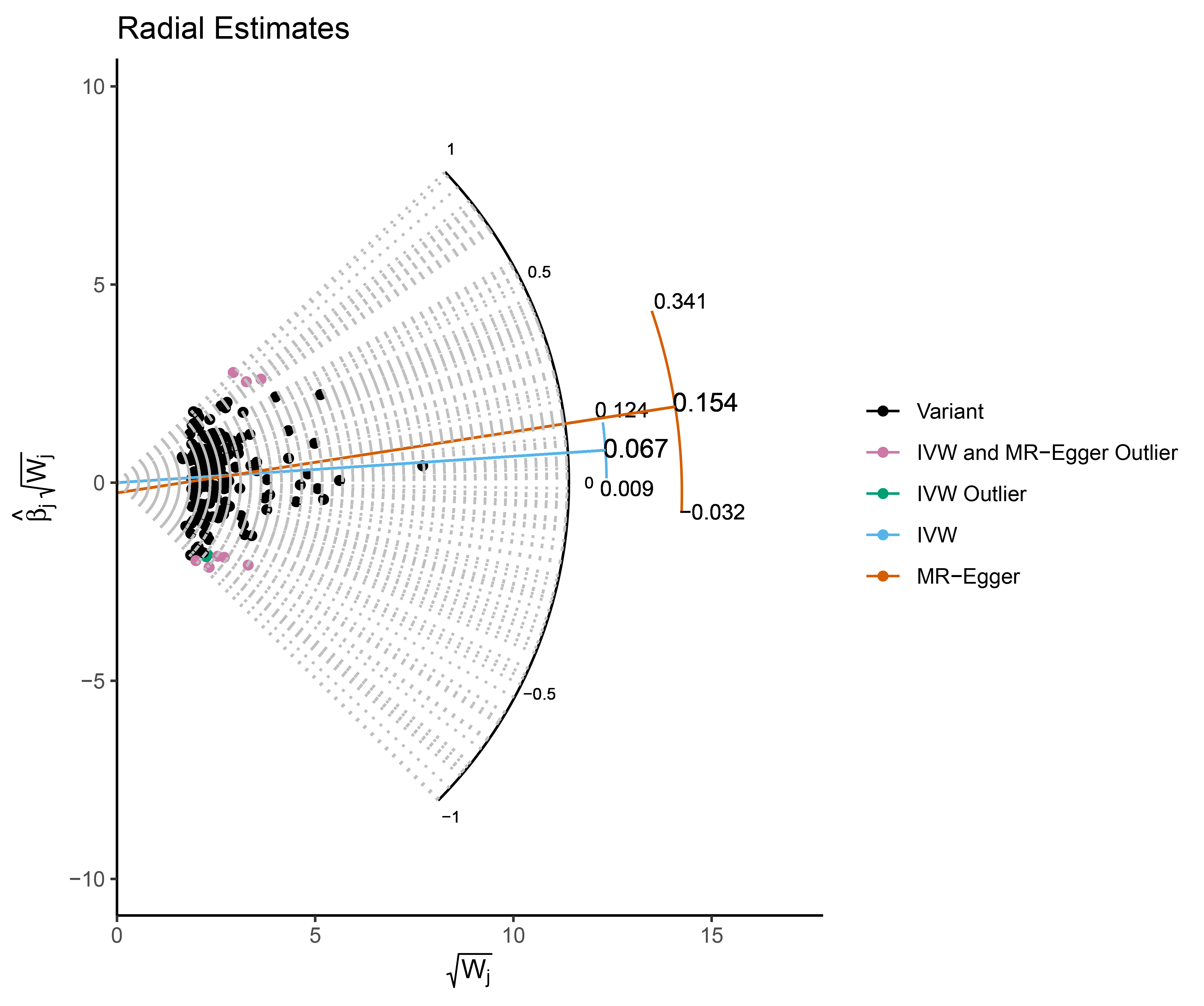

Supplement: S1 Data — (ZIP) [file pone.0309124.s002.zip › Data Sheet/Additional file 5 RadialMR plot figure/K2 ALM on cognitive function.tif]

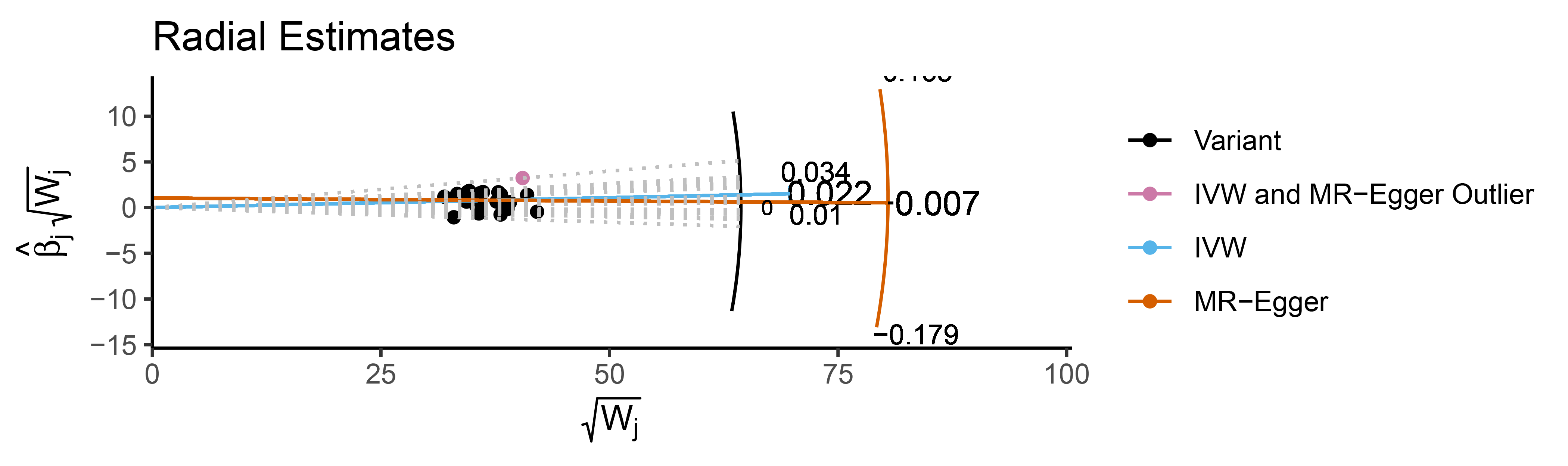

Supplement: S1 Data — (ZIP) [file pone.0309124.s002.zip › Data Sheet/Additional file 5 RadialMR plot figure/K20 Cognitive function on walking pace.tif]

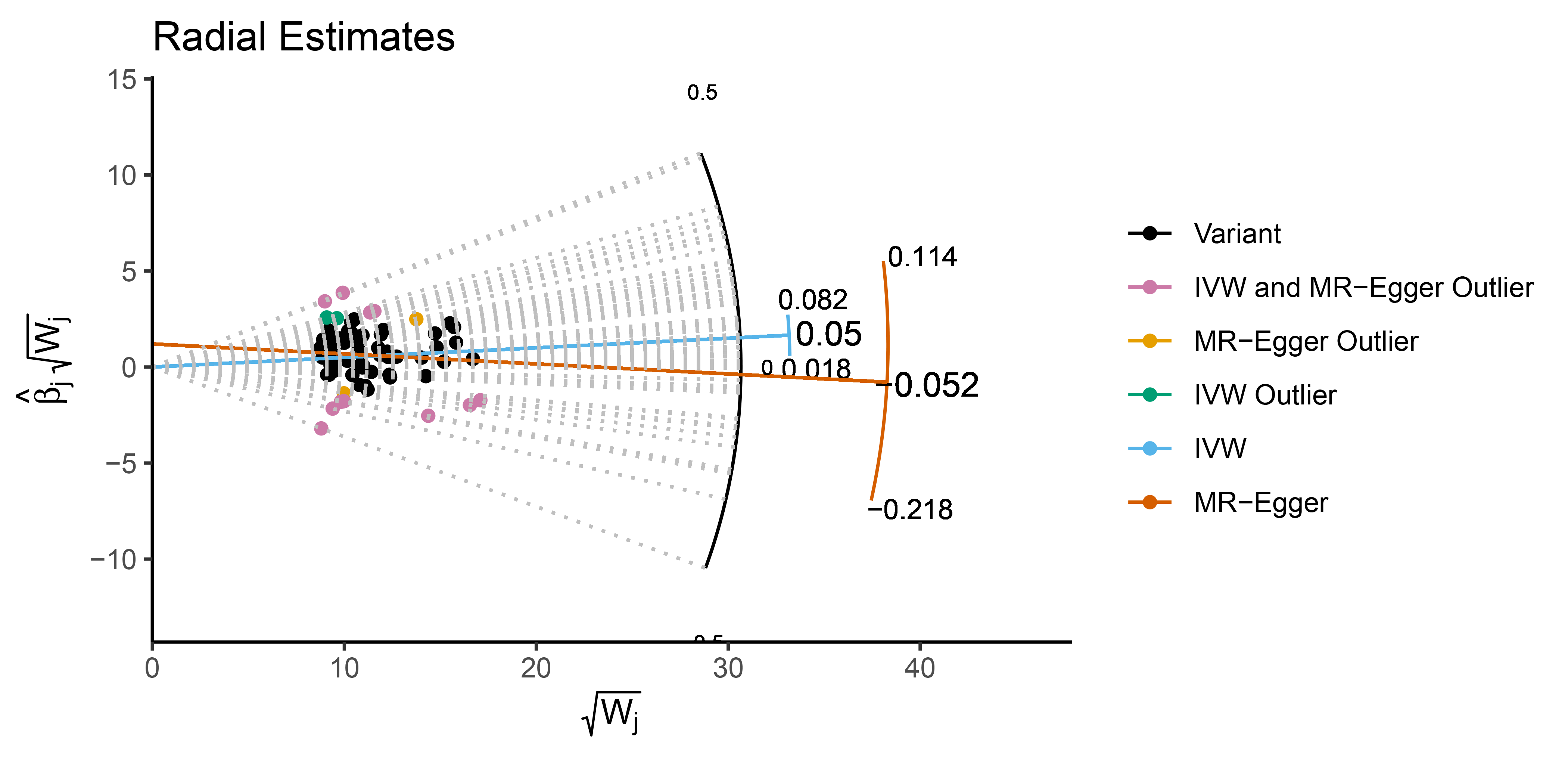

Supplement: S1 Data — (ZIP) [file pone.0309124.s002.zip › Data Sheet/Additional file 5 RadialMR plot figure/K3 ALM-M on cognitive performance.tif]

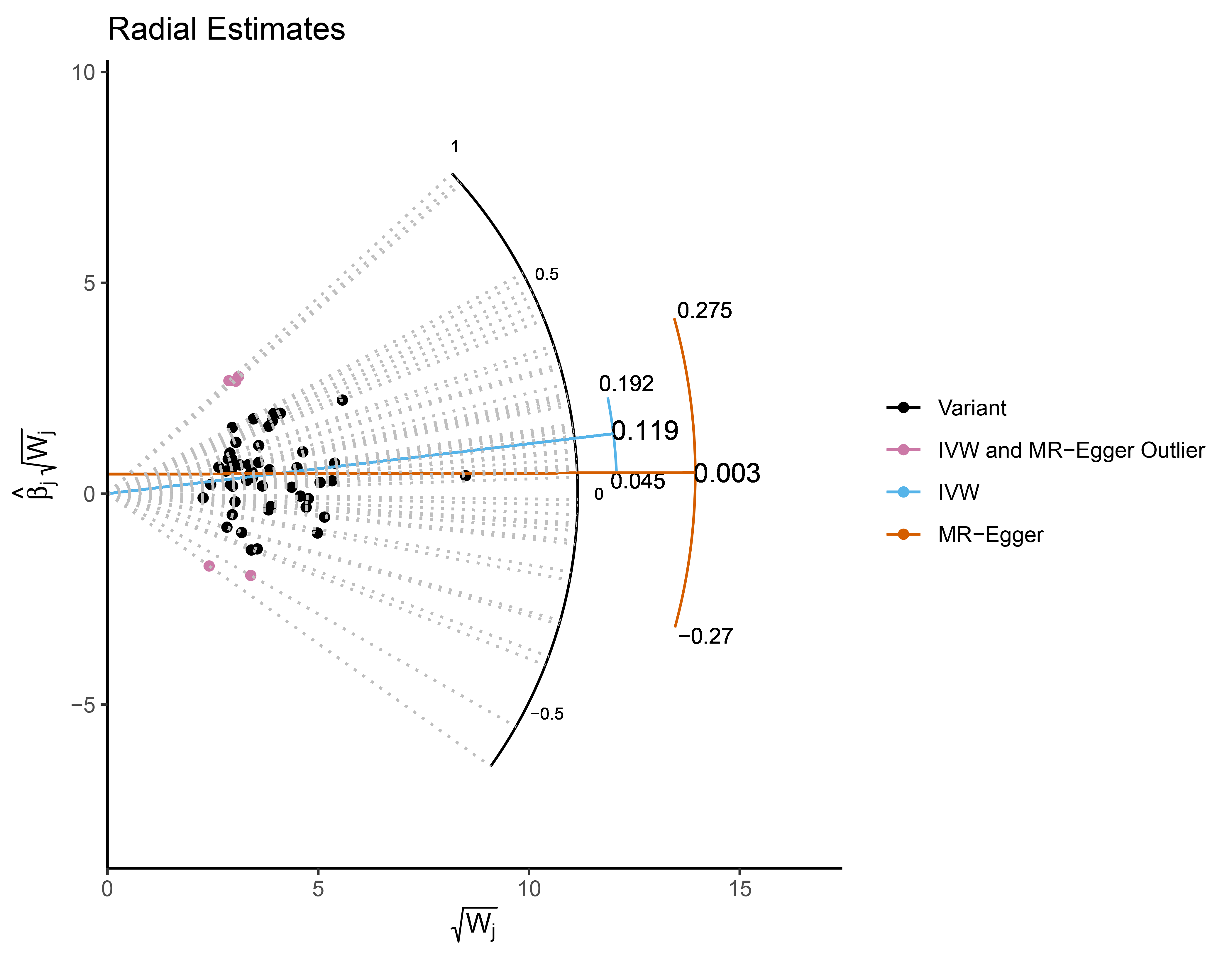

Supplement: S1 Data — (ZIP) [file pone.0309124.s002.zip › Data Sheet/Additional file 5 RadialMR plot figure/K4 ALM-M on cognitive function.tif]

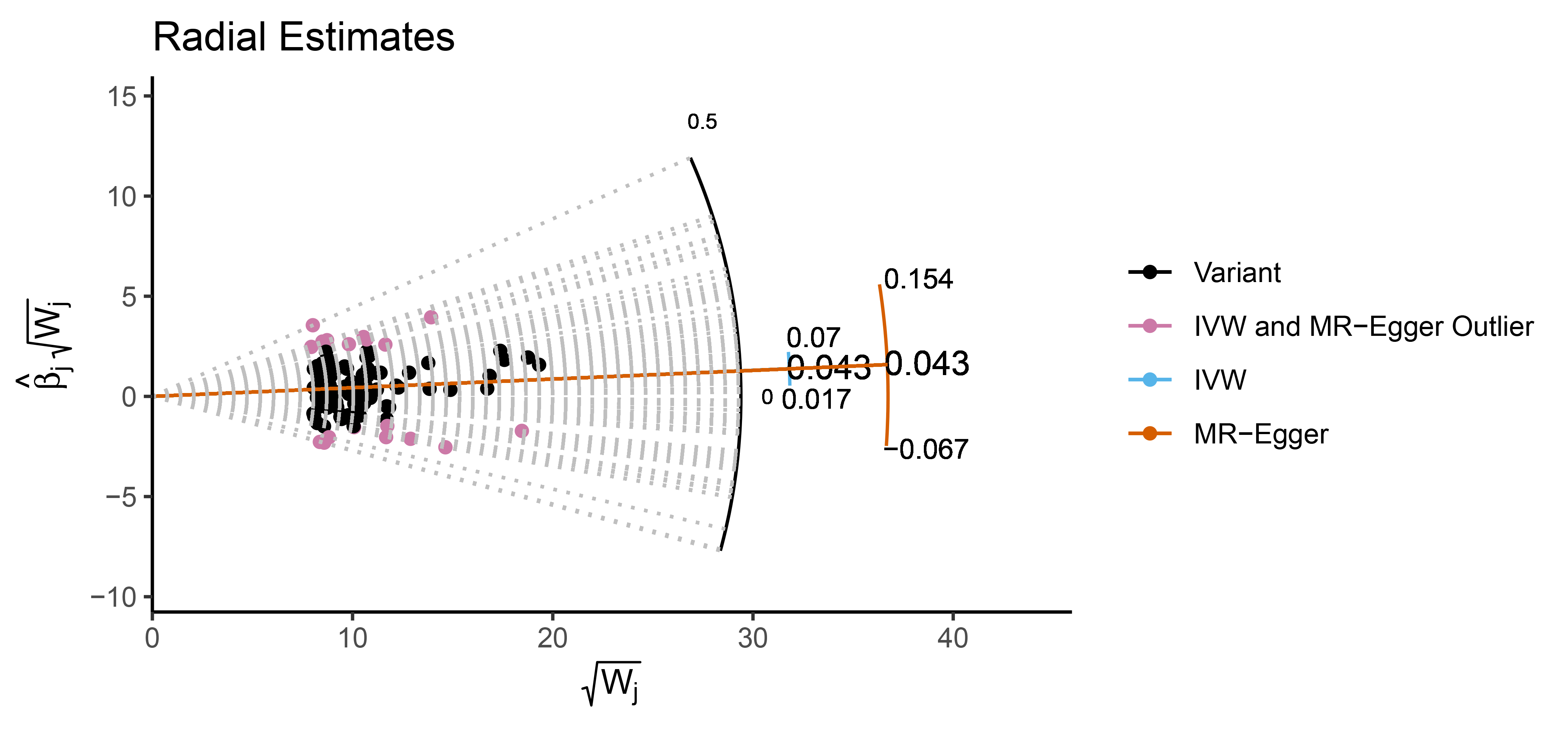

Supplement: S1 Data — (ZIP) [file pone.0309124.s002.zip › Data Sheet/Additional file 5 RadialMR plot figure/K5 ALM-F on cognitive performance.tif]

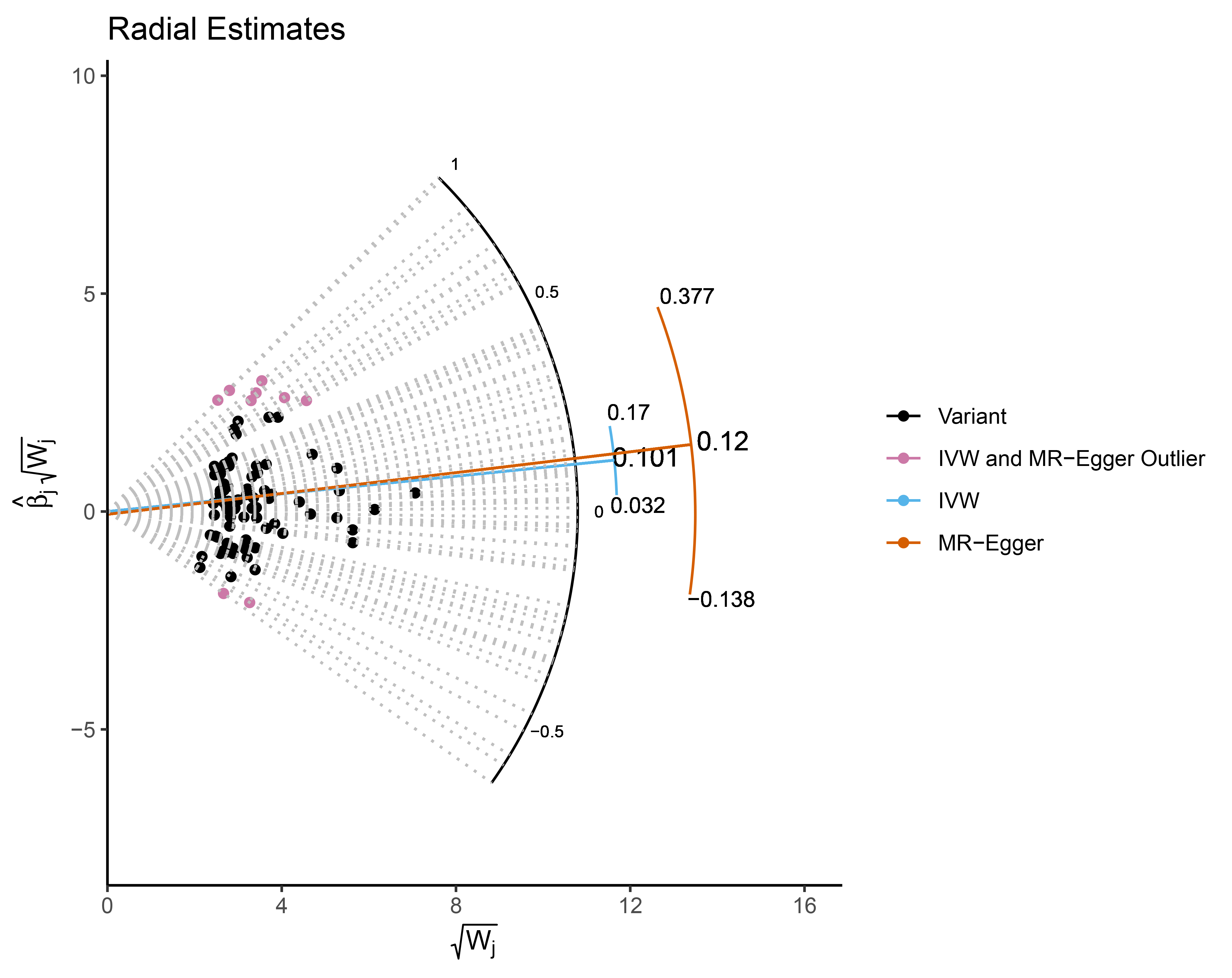

Supplement: S1 Data — (ZIP) [file pone.0309124.s002.zip › Data Sheet/Additional file 5 RadialMR plot figure/K6 ALM-F on cognitive function.tif]

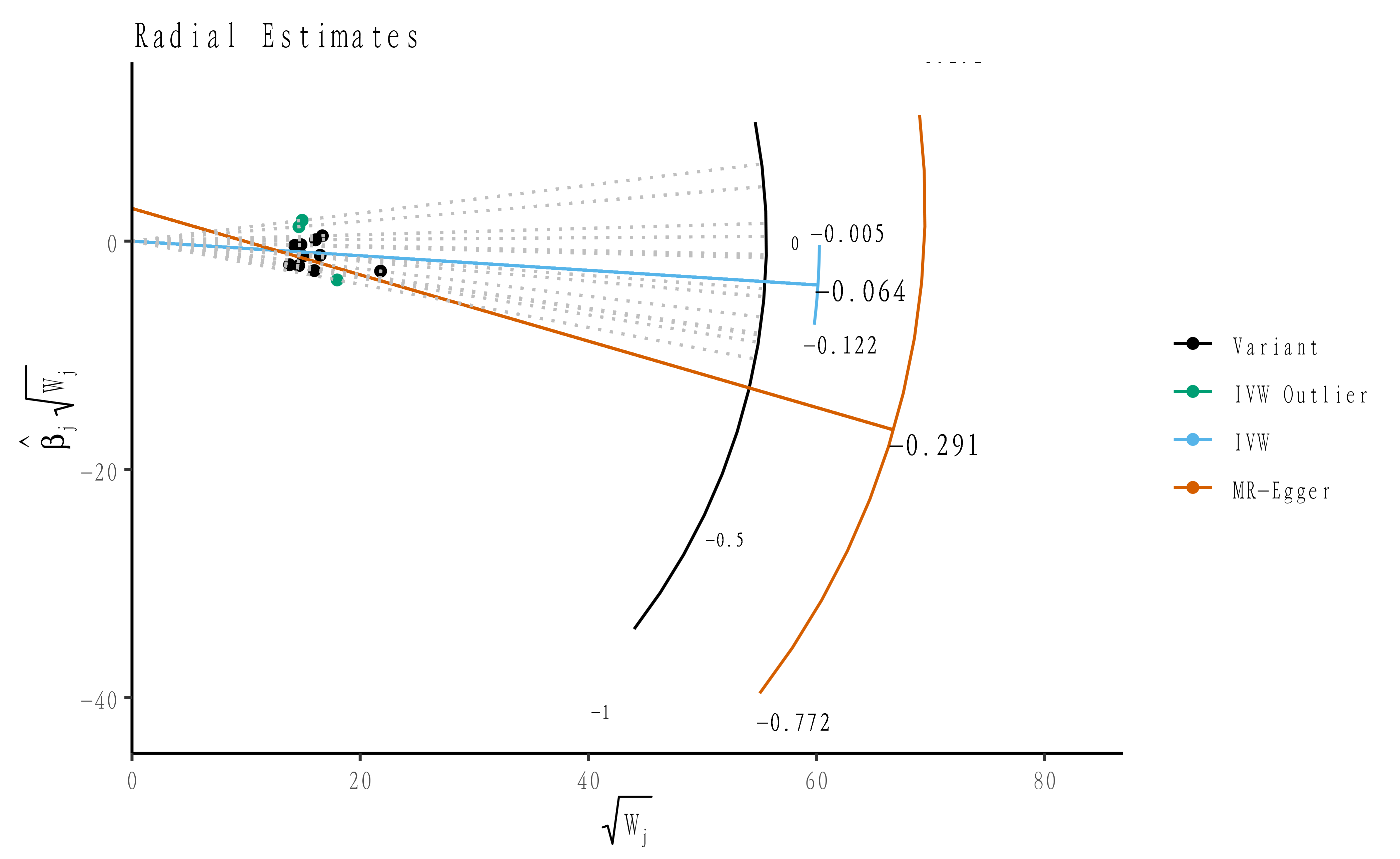

Supplement: S1 Data — (ZIP) [file pone.0309124.s002.zip › Data Sheet/Additional file 5 RadialMR plot figure/K7 Low hand grip strength on cognitive performance.tif]

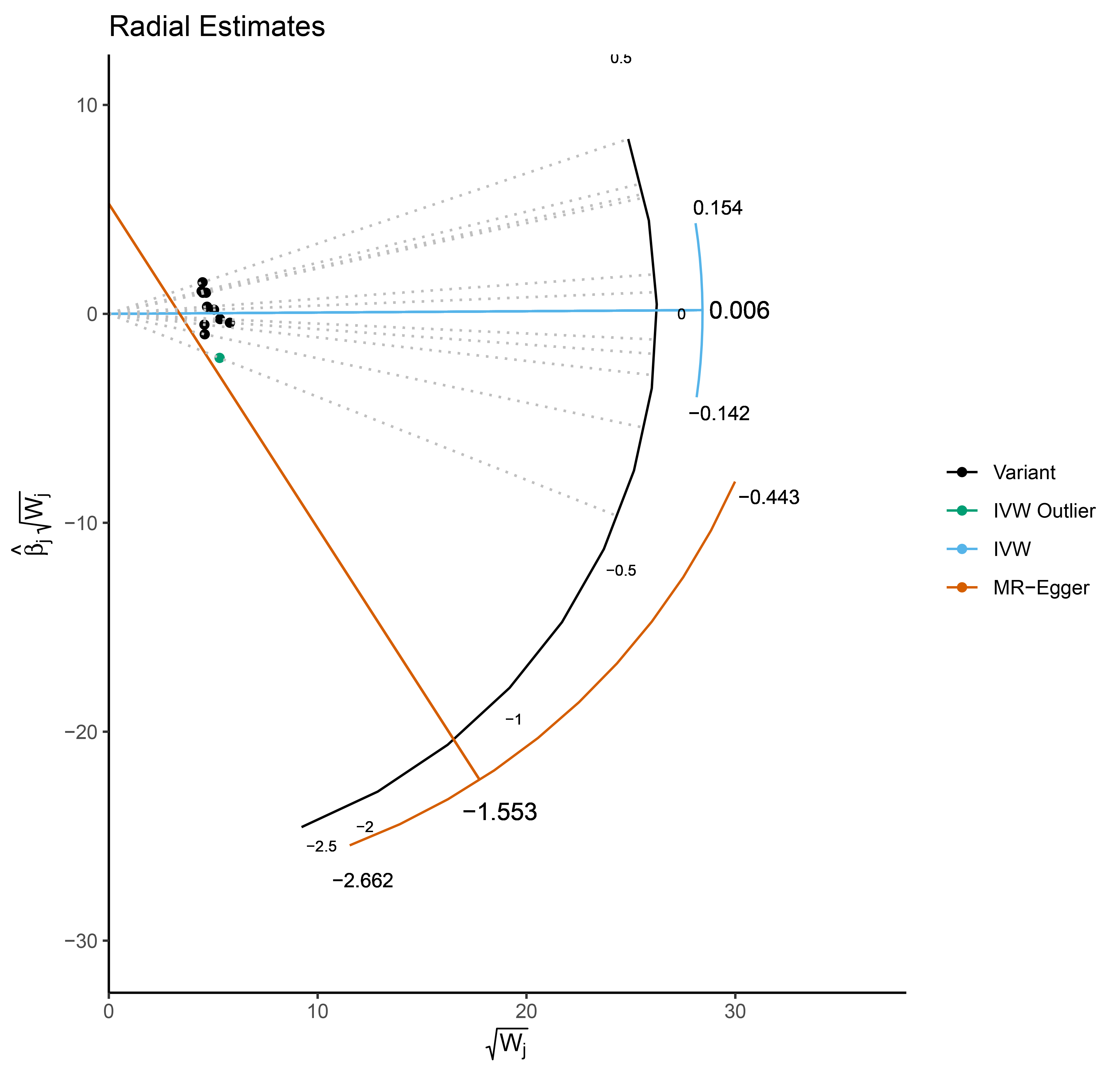

Supplement: S1 Data — (ZIP) [file pone.0309124.s002.zip › Data Sheet/Additional file 5 RadialMR plot figure/K8 Low hand grip strength on cognitive function.tif]

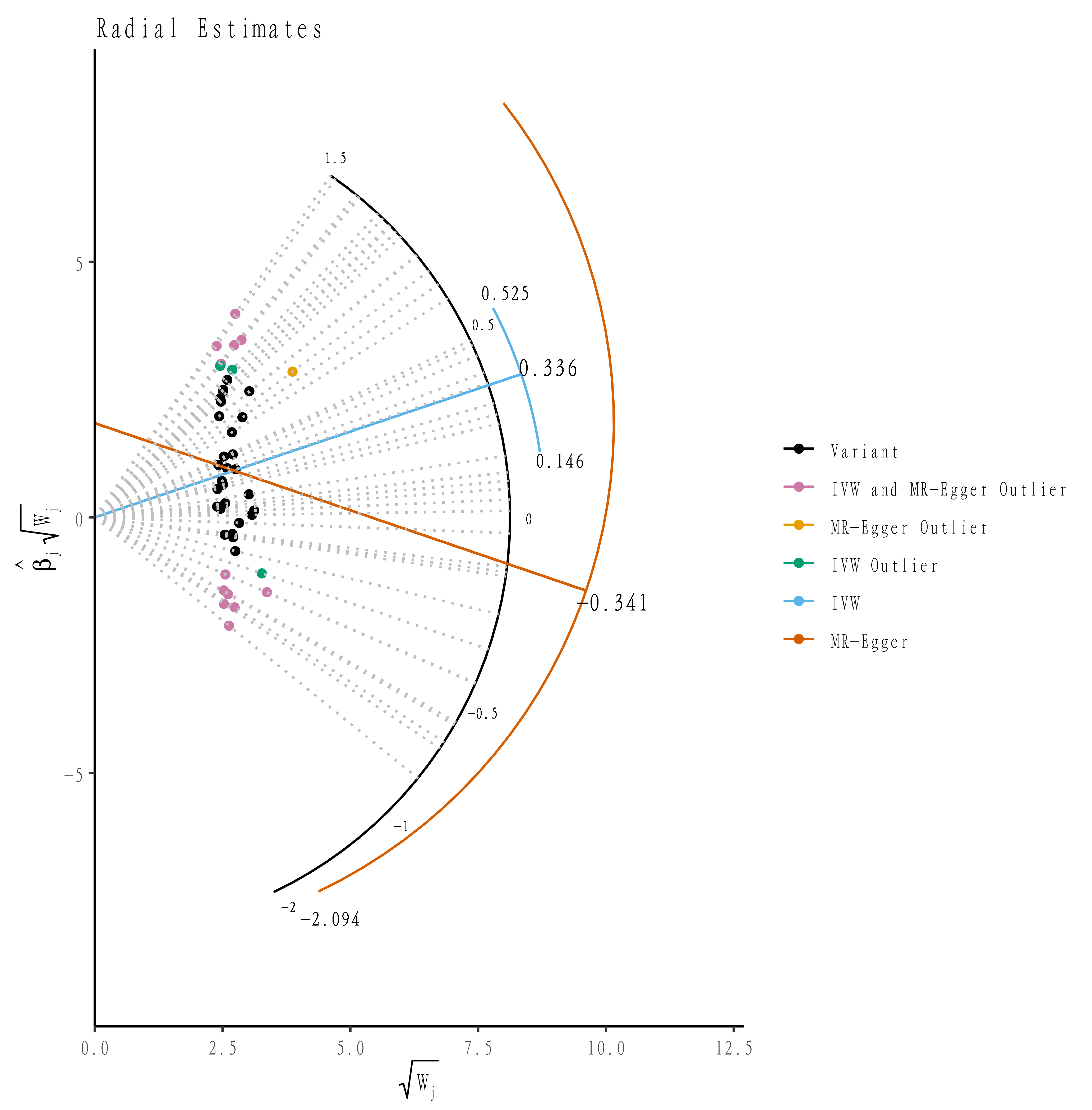

Supplement: S1 Data — (ZIP) [file pone.0309124.s002.zip › Data Sheet/Additional file 5 RadialMR plot figure/K9 Walking pace on cognitive performance.tif]
