# Supplementary material for: A causal relationship between sarcopenia and cognitive impairment: A Mendelian randomization study
Source: PLoS One. 2024 Sep 6;19(9):e0309124. doi: 10.1371/journal.pone.0309124 (PMC11379137; doi:10.1371/journal.pone.0309124)
Supplement: Attachment — Submitted filename: Response to Reviewers.docx [file pone.0309124.s003.docx]

**Response to reviewer’s comments**

**Reviewer #1**

Dear reviewer,

We feel great thanks for your professional review work on our article. As you are concerned, there are several problems that need to be addressed. According to your nice suggestions, we have made extensive corrections to our previous draft, the detailed corrections are listed below.

**Reviewer 1#**

First, the authors mention that they used multivariate Mendelian randomization, but I don't seem to find a specific description of what potential confounders were controlled for by MVMR in the paper.

**The author’s answer:**

As the three muscle traits associated with sarcopenia were interrelated, we performed MVMR1 to determine whether they were causally related to cognitive function, independent of other traits. We observed that IVW ultimately included 524 SNPs, and multivariate analyses showed significant causal associations between ALM (*β* = 0.077; 95% CI: 0.044–0.109, *P* = 0.000), walking pace (*β* = 0.579; 95% CI: 0.383–0.775, *P* = 0.000), and cognitive function. When smoking was included in MVMR2, IVW ultimately included 480 SNPs, and multivariate analyses showed that ALM causality remained (*β* = 0.069; 95% CI: 0.033–0.106, *P* = 0.000) and also walking pace (*β* = 0.589; 95% CI: 0.372–0.806, *P* = 0.000) **(Figure 2)**.

**Figure 2. Sarcopenia causality and cognitive impairment in multivariable models**

**Reviewer 2#**

Second, I recommend that the authors use linkage disequilibrium score regression to explore genetic correlations between phenotypes. This is the theoretical basis of the study.

**The author’s answer:**

LDSC was used to estimate genetic correlations between traits based on GWAS summary statistics. Genetic correlations between sarcopenia-related traits and cognitive function were assessed using cross-trait LDSC (1-3)

These analyses were performed to assess genetic associations between sarcopenia-related muscle characteristics and cognitive function. Analyses showed a causal relationship between ALM and walking pace and cognitive function, and a suggestive association between low grip strength and cognitive function (**Table 1**).

Table 1.The genetic correlations between sarcopenia and cognitive impairment

| trait | cognitive performance | | | cognitive function | | |
| --- | --- | --- | --- | --- | --- | --- |
|  | rg | SE | P-Value | rg | SE | P-Value |
| ALM | 0.153 | 0.017 | **0.000** | 0.216 | 0.037 | **0.000** |
| ALM-M | 0.129 | 0.019 | **0.000** | 0.187 | 0.040 | **0.000** |
| ALM-F | 0.168 | 0.019 | **0.000** | 0.239 | 0.040 | **0.000** |
| low hand grip strength  walking pace | -0.066  0.315 | 0.030  0.023 | 0.030  **0.000** | -0.122  0.410 | 0.061  0.047 | 0.046  **0.000** |

Reference: 1.	Bulik-Sullivan BK, Loh P-R, Finucane HK, Ripke S, Yang J, Patterson N, et al. LD Score regression distinguishes confounding from polygenicity in genome-wide association studies. Nat Genet. 2015;47(3):291-5.

2.	Bulik-Sullivan B, Finucane HK, Anttila V, Gusev A, Day FR, Loh P-R, et al. An atlas of genetic correlations across human diseases and traits. Nat Genet. 2015;47(11):1236-41.

3.	Gazal S, Finucane HK, Furlotte NA, Loh P-R, Palamara PF, Liu X, et al. Linkage disequilibrium-dependent architecture of human complex traits shows action of negative selection. Nat Genet. 2017;49(10):1421-7.

**Reviewer 3#**

Furthermore, there are significant sex differences in sarcopenia related phenotypes, which may also be associated with cognitive function. Therefore, gender stratification analysis is necessary. As far as I know, gender-stratified GWAS full summary data is currently available.

**The author’s answer:**

Genetic associations for ALM were derived from a GWAS of 450,243 UK Biobank (UKB) participants. Of these, 205,513 and 244,730 were of male and female European ancestry, respectively (4).This analysis has been supplemented here. No gender-stratified GWAS data were found for the remaining low grip strength and walking pace.

Reference:4.	Pei Y-F, Liu Y-Z, Yang X-L, Zhang H, Feng G-J, Wei X-T, et al. The genetic architecture of appendicular lean mass characterized by association analysis in the UK Biobank study. Commun Biol. 2020;3(1):608.

**Reviewer 4#**

Can the authors validate their results with data from other sources?

**The author’s answer:**

Cognitive functions used for the replication cohort were obtained from the Within family GWAS consortium and included 22,593 Europeans. Furthermore, GWAS meta analyses, thus obtained the final estimate of GWAS meta analysis. Bidirectional two-sample MR demonstrated that sarcopenia-related muscle characteristics and cognitive performance were positive causal genetic risk factors for each other, while a multivariable MR study demonstrated that low ALM and a slow walking pace were causally involved in reduced cognitive performance.

**Figure 1. Sarcopenia causality and cognitive impairment in univariable and meta-analysis models**

**Reviewer 5#**

Finally, I suggest that the author revise the article through a native speaker and correct any errors. I also suggested that the author rewrite the introduction, which I felt was lacking in the context. Among them, the introduction of why this study was conducted was not convincing.

**The author’s answer:**

Below are credentials for revising the article and correcting any errors by native speakers.

Founded in 2003 in the USA, BioMed Proofreading® is a leading English language copyediting company that focuses on copyediting, paraphrasing/rephrasing and translation of academic manuscripts in life sciences.

MR The use of genetic variants that are strongly associated with exposure to determine the causality of exposure and outcome reflects the effective effect of exposure on long-term effective effects on outcome. Genetic variants are randomly assigned at conception; they do not change with disease onset and course, they minimize the influence of external confounders and various biases on outcomes, and they are somewhat immune to small sample limitations (5, 6). Therefore, we investigated genetic correlations and causality between genetically predicted sarcopenia-related muscle traits and cognitive impairment using LDSC and MR. By clarifying the causal relationships between sarcopenia and cognitive impairment, we can guide and develop public health policy to reduce the risk of individuals developing both disorders.

Reference:5.	Carter AR, Sanderson E, Hammerton G, Richmond RC, Davey Smith G, Heron J, et al. Mendelian randomisation for mediation analysis: current methods and challenges for implementation. Eur J Epidemiol. 2021;36(5):465-78.

6.	Gao Q, Hu K, Yan C, Zhao B, Mei F, Chen F, et al. Associated Factors of Sarcopenia in Community-Dwelling Older Adults: A Systematic Review and Meta-Analysis. Nutrients. 2021;13(12).

We tried our best to improve the manuscript and made some changes marked in red in revised paper which will not influence the content and framework of the paper. We appreciate for Editors/Reviewers’ warm work earnestly, and hope the correction will meet with approval. Once again, thank you very much for your comments and suggestions.

Your sincerely,

Qin zhi Wang

wangqinzhi2024@163.com
